# A Type II CDK6 Degrader Enables Cellular Targeting beyond the Limits of Type II Inhibition

**DOI:** 10.1021/jacs.6c10277

**Published:** 2026-06-26

**Authors:** Ji Hyeon Kim, Caitlin E. Mills, Zuzanna Kozicka, Daniel C. Scott, Cyrus Jin, Zixuan Jiang, Brendan G. Dwyer, Qixiang Geng, Sean T. Toenjes, Woong Sub Byun, Dina ElHarouni, Hongyu Li, Keith L. Ligon, Abby M. Thornhill, Hannah M. Jones, Bryan A. Romero, Stephen M. Hinshaw, Benjamin L. Ebert, Brenda A. Schulman, Katherine A. Donovan, Eric S. Fischer, Nathanael S. Gray

**Affiliations:** † Department of Chemical and Systems Biology, ChEM-H, and Stanford Cancer Institute, Stanford School of Medicine, 10624 Stanford University, Stanford, California 94305, United States; ‡ Laboratory of Systems Pharmacology, Department of Systems Biology, Harvard Medical School, Boston, Massachusetts 02115, United States; § Department of Medical Oncology, Dana-Farber Cancer Institute, Boston, Massachusetts 02115, United States; ∥ Broad Institute of MIT and Harvard, Cambridge, Massachusetts 02142, United States; ⊥ ISIC, École Polytechnique Fédérale de Lausanne (EPFL), Lausanne CH-1015, Switzerland; # Department of Structural Biology, 5417 St. Jude Children’s Research Hospital, Memphis, Tennessee 38105, United States; ∇ Department of Cancer Biology, 1855 Dana-Farber Cancer Institute, Boston, Massachusetts 02115, United States; ○ Department of Biological Chemistry and Molecular Pharmacology, Harvard Medical School, Boston, Massachusetts 02115, United States; ◆ Department of Chemistry, Stanford School of Humanities and Sciences, Stanford University, Stanford, California 94305, United States; ¶ College of Pharmacy, Dongguk University-Seoul, Goyang 10326, Republic of Korea; 11 Department of Pathology, 1855 Dana-Farber Cancer Institute, Boston, Massachusetts 02115, United States; 12 Department of Molecular Machines and Signaling, Max Planck Institute of Biochemistry, Martinsried 82152, Germany

## Abstract

PROTACs are commonly developed by linking E3 ligase-recruiting ligands to established inhibitors of a protein target, often resulting in degraders that retain enzymatic inhibition. Type II inhibition of cyclin-dependent kinases (CDKs) has been challenging, as reported compounds generally exhibit weak biochemical potency and limited cellular activity. Consistent with these limitations, most reported CDK degraders have been derived from type I ATP-competitive inhibitors. Here, we explored whether targeted protein degradation could enable functional CDK targeting from a type II kinase scaffold. Using the multikinase inhibitor regorafenib as a starting scaffold, we generated a focused library of CRL4^CRBN^-recruiting bifunctional molecules and profiled their degradation activity using quantitative mass spectrometry-based proteomics. This analysis unexpectedly revealed CDK5 and CDK6, kinases not inhibited by the parent scaffold, as degradation targets. Optimization of this series led to **JHK-02–108–2**, a selective CDK6 degrader that does not display a hook effect and promotes potent CDK6 degradation despite weak CDK6 binding and negligible CDK6 inhibition. In cellular models of acute myeloid leukemia (AML) and glioblastoma, **JHK-02–108–2** induced sustained G1 arrest and reduced phosphorylation of the retinoblastoma protein. Interestingly, subtle modifications in PROTAC architecture redirected degradation selectivity, yielding **JHK-02–102–1** as a selective type II CDK5 degrader derived from the same scaffold. Together, these findings establish the first type II inhibitor-derived selective CDK6 degrader and demonstrate that targeted protein degradation can enable functional CDK targeting from type II kinase scaffolds.

## Graphical abstract

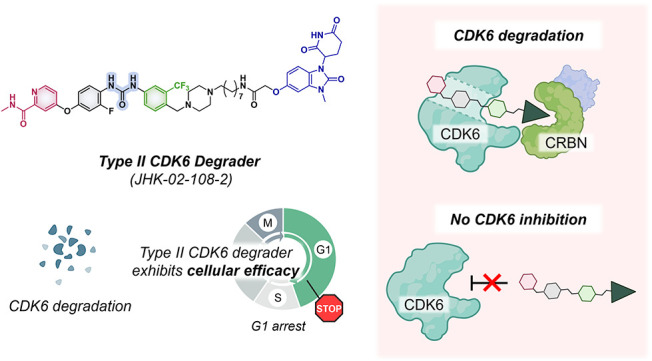

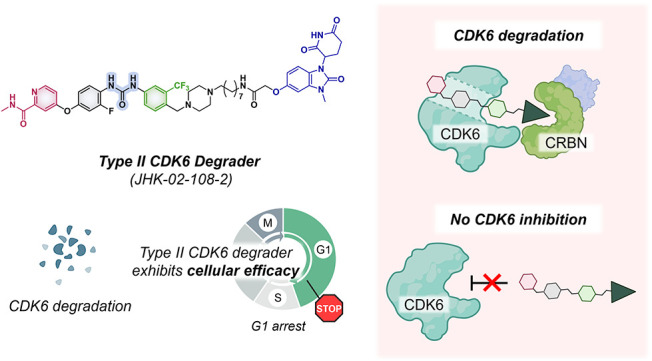

## Introduction

Targeted protein degradation (TPD) has emerged as a promising therapeutic strategy for the selective degradation of disease-causing proteins, with proteolysis-targeting chimeras (PROTACs) representing a leading technology in this field.^1,2^ PROTACs are heterobifunctional small molecules that simultaneously bind a target protein and an E3 ubiquitin ligase, promoting induced proximity and subsequent ubiquitination and proteasomal degradation of the target.^3^ Unlike traditional inhibitors, PROTACs can be constructed from ligands that lack functional activity and can target nonenzymatic “scaffolding” functions of proteins.^4,5^ Despite advances in the field, most PROTACs remain targeted toward proteins with known inhibitors, as design commonly begins by linking an established binder to an E3-recruiting motif.^6^ While effective for rational design, this binder-centric strategy inherently confines discovery to previously liganded proteins. Recently, “degrader-first” studies have offered unbiased routes to identify degradation substrates of small molecules with no established targets, as exemplified by Racioppo et al. and Forrest et al.^7,8^ These approaches highlight the feasibility of discovering degradable proteins beyond the scope of traditional inhibitor repurposing.

Type II inhibitors engage the inactive (DFGout) kinase conformation and extend into an adjacent hydrophobic pocket, affording unique selectivity relative to type I (DFGin) inhibitors.^9–13^ However, type II PROTAC scaffolds remain underexplored, partly due to their higher molecular weights and limited potency across many targets, including cyclin-dependent kinases (CDKs).^14–16^ For CDK targets, with the exception of CDK2, CDK5, and CDK8, type II CDK inhibitors have been reported but exhibited weak biochemical potency and poor cellular activity.^13,17,18^ Given these challenges, to our knowledge, no type II CDK degraders have been reported.

As summarized in Table S1 and Figure S1, the majority of reported CDK degraders are derived from type I kinase inhibitors and often retain the inhibitory activity of the parent scaffold. This overlap can complicate the attribution of cellular phenotypes to degradation versus inhibition. Although several broad-spectrum degraders have been shown to target CDKs, they lack the selectivity required to disentangle these effects (Table S2 and Figure S2).^19^

Here, we explored whether derivatization of the type II multikinase inhibitor regorafenib could enable selective CDK degradation despite weak intrinsic binding affinity. We evaluated how derivatization of regorafenib at two distinct exit vectors—the front of the hinge-binding region (“front” degraders) and the back of the hydrophobic pocket (“back” degraders)—affects degradation and enrichment profiles (Figure 1A). We hypothesized that a type II inhibitor-stabilized conformation, together with distinct linkage sites, could promote alternative ternary complexes with CRBN and thereby a degradation spectrum distinct from type I-derived PROTACs. Consistent with this hypothesis, the kinase degradation profiles were markedly distinct from the corresponding inhibition profiles (Figure S3), with degradation observed across multiple CDK family members. Moreover, the back-recruiter-derived PROTAC, **STT-03–123**, enriched and degraded a broader set of kinases than the front-recruiter design, **STT-03–122** (Figure 1B).

**Figure 1:**
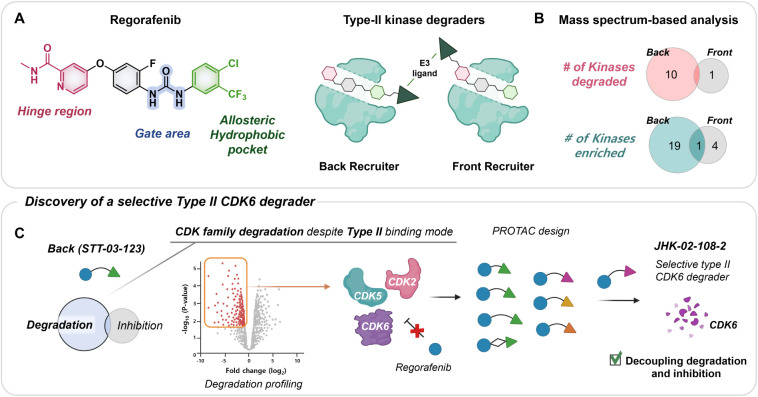
Concept and discovery of regorafenib-derived type II CDK6 degraders. (A) Regorafenib-based type-II kinase-binding PROTACs with two different exit vectors. (B) Venn diagram comparison of kinase degradation and enrichment profiles for **STT-03–123** (back recruiter) and **STT-03–122** (front recruiter), based on quantitative mass spectrometry analysis. (C) Discovery of unexpected CDK family degradation by type II degraders and subsequent optimization toward a selective CDK6 degrader.

Further chemical structure modification afforded **JHK-02–108–2**, a type II, inhibition-decoupled degrader that selectively targets CDK6 (Figure 1C). In addition, a related analog, **JHK-02–102–1**, functions as a selective CDK5 degrader, illustrating that modest changes in linker and CRBN engagement can redirect degradation within the CDK family. In contrast to the previously reported type I CDK6 degrader **BSJ-03–123**, which retains kinase inhibition, our type II CDK6 degrader decouples degradation from inhibition, enabling selective target degradation without measurable inhibitory activity toward CDK6.

## Results and Discussion

### Degradation Profile of Regorafenib-Based Type II “Back” Degrader

Regorafenib is an FDA-approved type II multikinase inhibitor used in the treatment of metastatic colorectal cancer, gastrointestinal stromal tumors (GIST), and hepatocellular carcinoma (HCC).^20^ Its type II binding mode involves engagement of the ATP-binding site and extension into the hydrophobic back pocket created by the DFG-out flip, enabling the activity against a broad spectrum of kinases, including VEGFR1–3, PDGFR, FGFR, KIT, RET, and BRAF.^21–24^ We synthesized a “front” CRBN-recruiting (**STT-03–122**) and a “back” CRBN-recruiting (**STT-03–123**) degrader, and we assessed their degradation and profiles using global proteomics (Figures 2A and S4). A large number of kinases were unexpectedly degraded by **STT-03–123**, which utilizes a “back” exit vector, a hydrophobic pocket that is not the intuitive solvent-exposed exit vector (Figure 2B). **STT-03–123** induced degradation of kinases previously reported as degradable targets,^19^ including MAP3K7, LCK, and IRAK1. Notably, it also led to the degradation of CDK1, CDK5, CDK6, and CDK18, which are not canonical regorafenib targets.^22^ In contrast, **STT-03–122**, featuring a “front” recruitment site, led to the degradation of only a limited set of kinases, such as MAPK9 (Figure S4). To broadly profile what induced protein–protein interactions are enabled by the heterobifunctional degraders, we used a lysate-based immunoprecipitation mass spectrometry (IP-MS) experiment in which immobilized Flag-CRBN-DDB1Δ*B*is used to identify proteins recruited to CRBN in the presence of **STT-03–123** in complex cell lysate.^25^ CDK2, CDK5, and LCK were among the most strongly enriched targets, and a large number of proteins, including both kinases and nonkinases, were also found to be recruited by the regorafenib scaffold (Figure S5). In contrast, **STT-03–122** enriched only five kinases, with MAPK9 being the only kinase both enriched and degraded by this compound (Figure S6).

**Figure 2:**
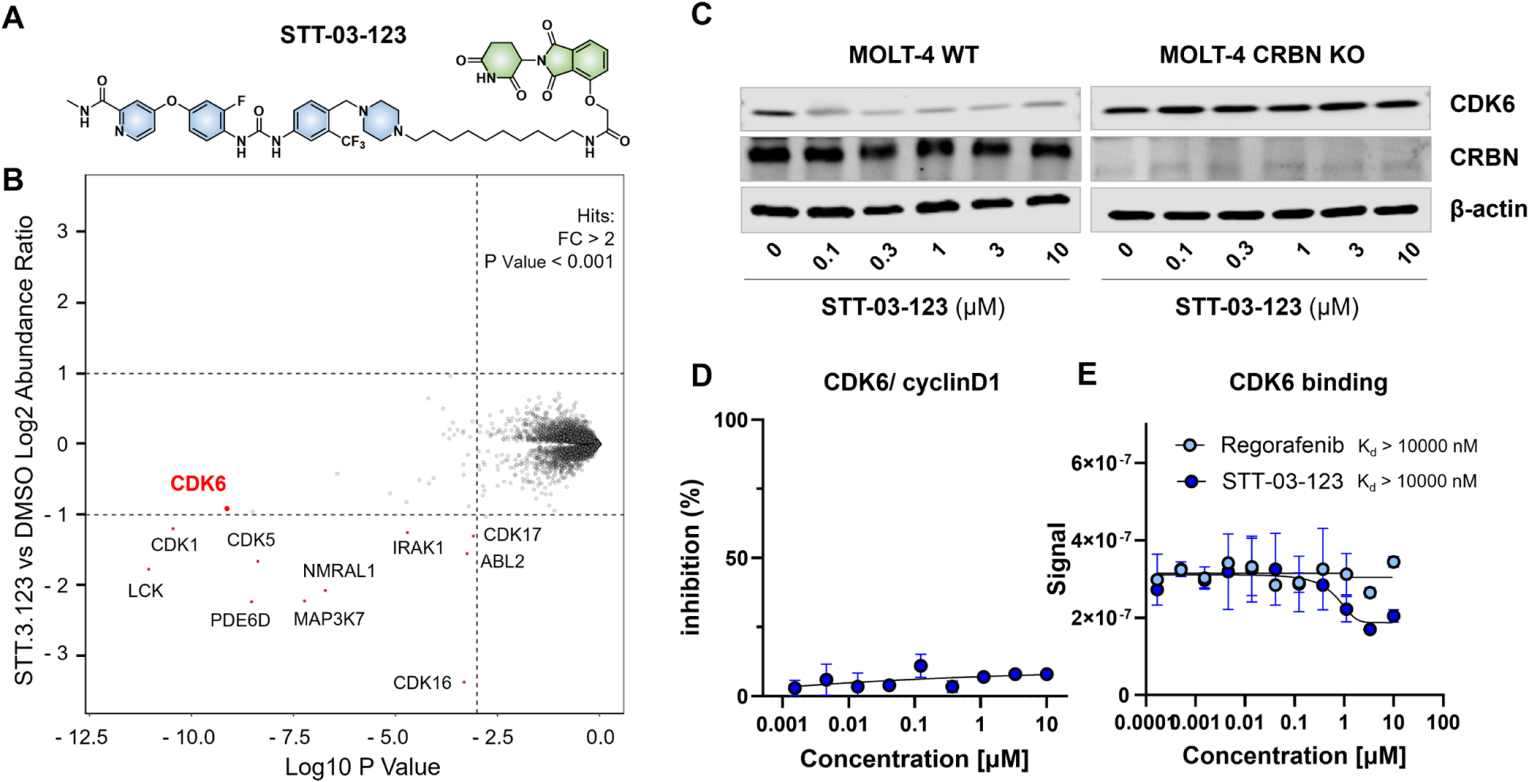
Regorafenib-scaffold “back” exit vector bivalently degraded diverse kinases beyond its inhibition target. (A) Chemical structure of **STT-03–123**. (B) Global proteomics analysis of MOLT-4 cells treated with **STT-03–123** for 5 h. (C) Immunoblot validating CDK6 degradation **STT-03–123** in MOLT-4 WT and MOLT-4 CRBN KO cells for 5 h. (D) Dose-dependent inhibition of CDK6/cyclin D1 activity by **STT-03–123**, measured using the Adapta universal kinase assay. (E) Dose-dependent binding curve of regorafenib and **STT-03–123** to CDK6 as determined by the KINOMEscan KdELECT assay. The binding affinity (K_d_) was calculated from an 11-point 3-fold serial dilution.

CDK6 was weakly enriched in the CRBN IP-MS, sitting slightly below our “hit” threshold of FC > 2; P-value <0.001. To orthogonally validate CDK6 as a hit and assess whether recruitment leads to productive degradation, we performed Western blot analysis to examine the degradation of CDK6 following treatment with **STT-03–123** (Figure 2C). We observed dose-dependent degradation of CDK6 protein, whereas no degradation of CDK6 was detected in CRBN knockout MOLT-4 cells, confirming that degradation is CRBN-dependent.

We next evaluated the kinase inhibition activity of the regorafenib-derived scaffold against CDK6/cyclin D1 complexes. As expected, the regorafenib-derived scaffold does not inhibit CDK6 even when incorporated into a bifunctional degrader (Figure 2D). Nevertheless, as members of the CDK family closely associate with regulatory subunits to exert their kinase activity, we reasoned that even in the absence of inhibitory activity, the kinase-binding moiety could still interact within the kinase-binding pocket. Therefore, we measured the binding affinity (K_d_) of the regorafenib and **STT-03–123** for CDK6 (Figure 2E). As expected, regorafenib showed no detectable binding to CDK6 up to 10 μM. Upon incorporation into a PROTAC (**STT-03–123**), weak but measurable binding to CDK6 was observed, with K_d_ values exceeding 10 μM. Although the regorafenib back-exit degrader neither inhibited CDK6 activity nor displayed strong binary binding, it effectively induced CDK6 degradation, implying productive ternary complex formation. Subsequent chemistry efforts focused on optimizing features of the scaffold to enhance degradation efficiency, selectivity, and cellular potency.

### Structure–Activity Relationship (SAR) Optimization of JHK-02–108–2 as a Selective CDK6 Heterobifunctional Degrader

Our goal was to understand the structure–activity relationship (SAR) for CDK6 degradation using regorafenib-based PROTACs, with the aim of developing selective degraders. Given the CDK degradation profile of **STT-03–123**, we investigated the SAR of other heterobifunctional degrader scaffolds by modifying diverse structures of CRBN binders and linkers (Figure 3A and Figure S7).^26,27^ In the initial round of screening, alternative CRBN binders (CRBN binder-B and -C) provided improved CDK5 degradation in **JHK-02–078–2** and **JHK-02–063–1** (Figure 3B). Meanwhile, enhanced CDK6 degradation was achieved with **JHK-02–078–2** and **JHK-02–078–3** using CRBN binder-C and -D. Replacing the linker from C10 to PEG3 abolished CDK6 degradation, and modifying the piperazine linker from a tertiary amine to an amide bond led to the loss of both CDK5 and CDK6 degradation (**JHK-02–080–1** and **JHK-02–080–2**). Furthermore, the introduction of a dimethyl group at the hinge-binding region of the regorafenib scaffold in **JHK-02–095** abolished the degradation of kinase targets, demonstrating the importance of catalytic site engagement for the recognition of kinase targets (Figure S8). Since the regorafenib scaffold has very low affinity toward CDK6 (Figure 2E), we evaluated whether the degradation of CDK6 is mediated by ternary complex formation with CRBN using a NanoBRET assay. **JHK-02–078–3** induced a concentration-dependent increase in BRET signal with CDK6, quantitatively comparable to that observed for **BSJ-03–123**,^28,29^ a known CRBN-based CDK6 degrader used as a positive control (Figure 3B and Figure S9).

**Figure 3:**
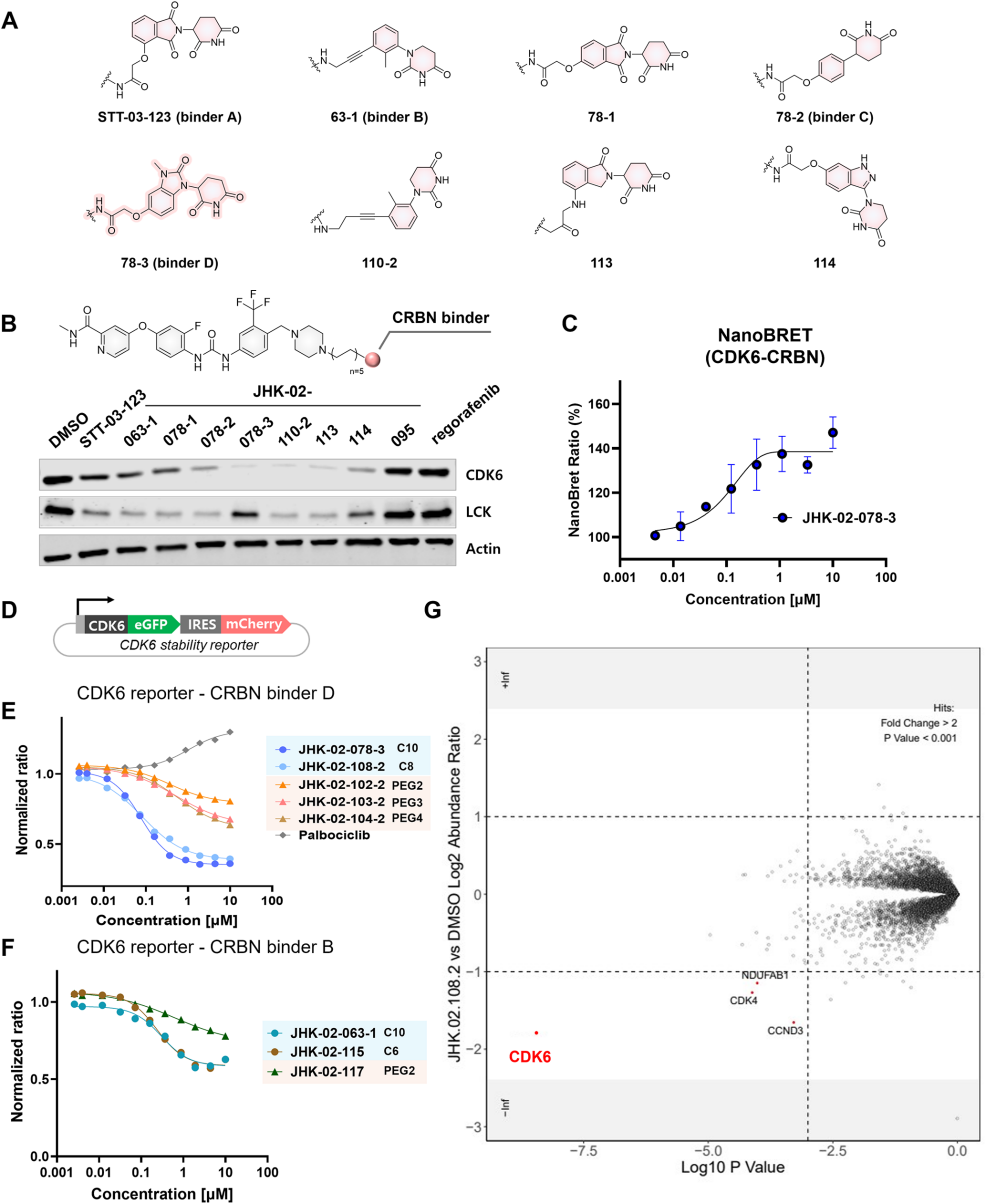
Structure–activity relationship (SAR) analysis of regorafenib-based PROTACs yields a selective type-II CDK6 degrader. (A) Chemical structures of different CRBN ligands employed in each compound. (B) Western blots of CDK5, CDK6, and LCK degradation in MOLT-4 cells treated with 1 μM of each compound for 5 h. (C) NanoBRET assay suggesting the induced interaction between HaloTag-CRBN and Nanoluciferase-CDK6 fusion proteins with **JHK-02–078–3**. (D) Schematic of the CDK6 stability reporter. IRES, an internal ribosome entry site. Concentration-dependent degradation of CDK6_eGFP_ evaluated by flow cytometry in K562-Cas9 cells treated with regorafenib-based bivalent degraders having (E) CRBN binder-D and (F) CRBN binder-B. (G) Volcano plots from global proteomics showing selective CDK6 degradation with 1 μM of **JHK-02–108–2** in MOLT-4 cells treated for 5 h.

We next focused on linker modifications to optimize target selectivity and enhance degradation potency toward CDK6 (Figure S10). To enable a side-by-side comparison of the degradation profiles of each compound, we constructed a fluorescent reporter of CDK6 protein levels. In this reporter system, CDK6 was fused to enhanced green fluorescent protein (eGFP) to generate a fluorescent CDK6–eGFP fusion protein, and degradation was quantified by normalizing CDK6–eGFP levels to mCherry expression, used as an internal control (Figure 3D).^30^ Since CDK6 degradation appeared favorable with CRBN binder-B and -D in preliminary screening (Figure 3B), we further investigated the linker SAR of heterobifunctional degraders incorporating these two binders. CDK6 degradation was strongly favored by carbon-based linkers compared to PEG linkers in the case of CRBN binder-D (Figure 3E). Building on the potent CDK6 degradation activity of **JHK-02–078–3** (binder D, C10 linker), we shortened the linker to C8 to generate **JHK-02–108–2**, which retained CDK6 degradation in the reporter assay while displaying improved selectivity in global proteomics, avoiding off-target degradation of CDK5, PDE6D, and other proteins observed with **JHK-02–078–3** (Figure 3G and Figure S11). With the CRBN binder B, carbon-based linkers still appeared to be preferred over PEG linkers; however, the overall CDK6 degradation efficiency was much lower than that observed with the CRBN binder D–based structures (Figure 3F). As a result, we developed a selective type-II CDK6 degrader, **JHK-02–108–2**, which was confirmed using global proteomics.

### Kinome-Wide Enrichment and Degradation Profiles of Type II Regorafenib-Derived PROTACs

To broadly characterize protein degradation and degrader-induced protein–protein interactions, we performed global proteomics and interactomics (CRBN IP-MS) analyses with nine CRBN-based regorafenib-derived PROTACs (Figure S11-S16, Figures 2B, 3G, and 7B). Significant changes were defined using a specified hit threshold of above 2-fold change and p-value of <0.001. We note that enrichment in CRBN IP-MS reflects degrader-induced target recruitment to CRBN, whereas degradation in global proteomics measures the subsequent proteasomal turnover of the target.^25^ Although not all recruited proteins are ultimately degraded, increased enrichment often correlates with productive ternary complex formation that precedes degradation. Among the CDK family members, CDK8 was consistently enriched across all nine PROTACs, yet no degradation was detected (Figure 4A). CDK5 emerged as the most frequently degraded target, being degraded by eight out of the nine degraders. **JHK-02–108–2** was the only degrader that did not induce CDK5 degradation (Figure 4B). In contrast, CDK2 degradation was observed in only three degraders, although both CDK5 and CDK2 were enriched in eight out of the nine compounds. Non-CDK kinases, including FER, AURKA, AURKB, LCK, MAP3K7, and MAP4K5, were also prominently enriched (Figure 4A). Among these, MAP3K7 and LCK were degraded by four degraders.

**Figure 4:**
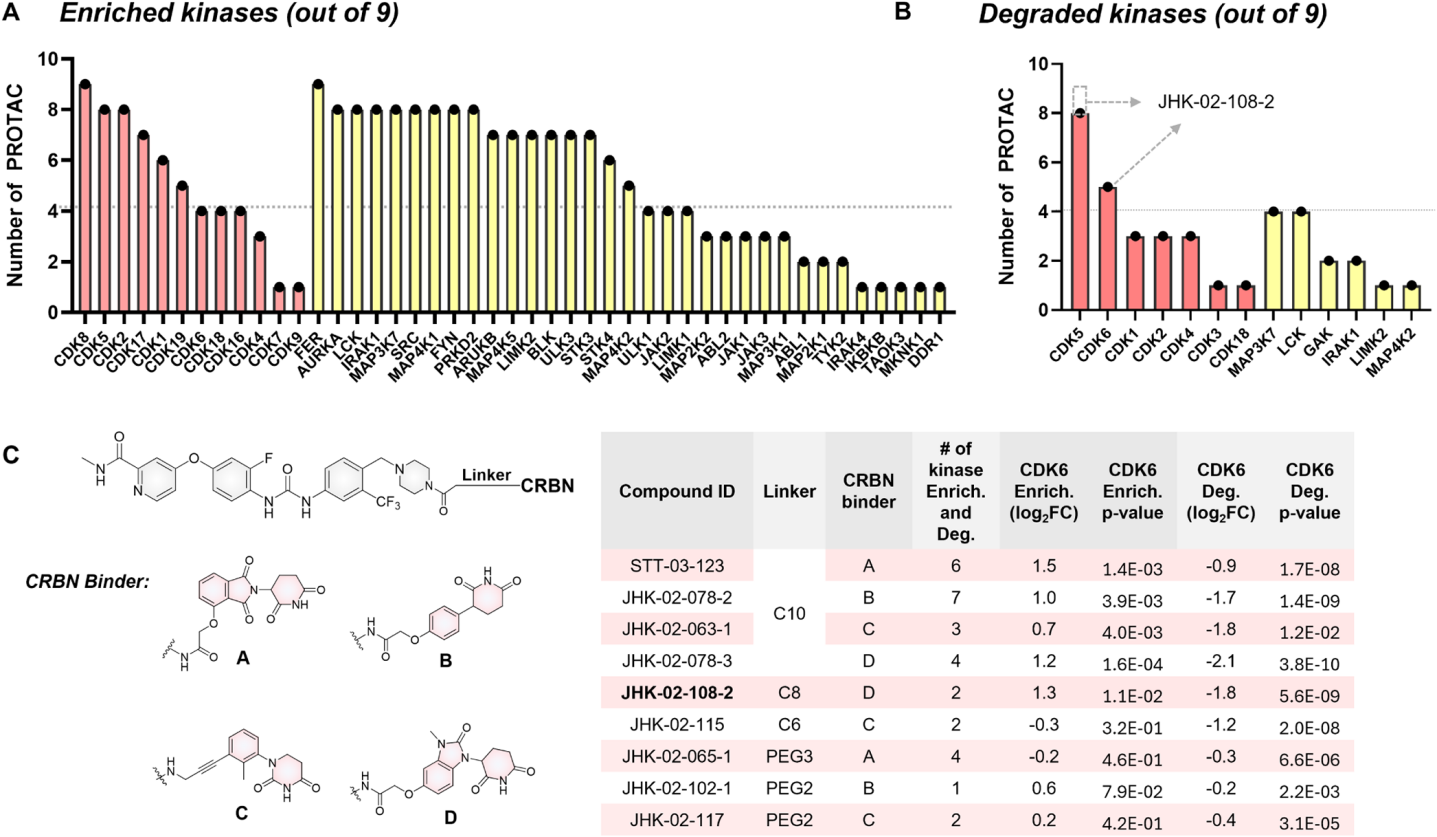
Divergent kinase enrichment and degradation profiles of regorafenib-derived back-recruiter PROTACs. (A) The number of independent degraders (out of 9) for which enrichment was observed for each kinase. Pink: CDK family members; yellow: non-CDK kinases. (B) The number of independent degraders (out of 9) for which degradation was observed for each kinase. (C) Number of proteins both enriched and degraded by each PROTAC shown in relation to the linker and CRBN binder used in the degrader design. CDK6 enrichment and degradation Log_2_FC and p-values in response to treatment with each compound (1 μM), as measured by CRBN-based IP-MS (enrichment) and whole-proteome analysis (degradation). Data extracted from Figures S10-S15.

Although many kinases were broadly enriched in the CRBN IP–MS analyses, most did not undergo degradation. In contrast, CDK6 showed the opposite trend: only four compounds exceeded the enrichment cutoff, yet CDK6 was degraded by as many as five degraders, suggesting that even limited CRBN engagement can drive productive ternary complex formation and proteasomal degradation (Figure 4C). To understand how linker composition influences target selectivity and degradation efficiency, we compared the degradation profiles of PROTACs bearing different linker lengths and types. PROTACs with longer linkers (PEG3 or C10) enriched and degraded a broader set of targets, including MAP3K7, whereas shorter linkers (C8 or PEG2) favored more selective profiles (Figure 4C). The correlation between enrichment and degradation (log_2_FC values) for CDK6 was weak. Together, these data demonstrate that linker and CRBN binder architectures shape the selectivity and breadth of target engagement of regorafenib-based degraders. Furthermore, CRBN-based IP-MS enables the identification of potential degradation targets beyond those predicted by conventional inhibition profiles of type II kinase inhibitors.^31^

### JHK-02–108–2 Efficiently and Selectively Degrades CDK6 and Forms a Ternary Complex Between CRBN and CDK6

To confirm that CDK6 degradation by the regorafenib-based PROTACs occurs via the ubiquitin-proteasome system (UPS), we performed rescue experiments using the neddylation inhibitor MLN4924 with **JHK-02–078–3** (type II CDK6 degrader) (Figure S17). In addition, pretreatment with MG132, a proteasome inhibitor, also rescued CDK6 degradation by **JHK-02–078–3**, consistent with the idea that CDK6 degradation is mediated through a UPS-dependent mechanism. Through global proteomics assessment, we identified **JHK-02–108–2** as a more selective CDK6 degrader than **JHK-02–078–3** and therefore focused our cellular studies on **JHK-02–108–2**. To distinguish whether the observed effects arise from CRBN-dependent CDK6 degradation or from the intrinsic kinase inhibition profile of the regorafenib scaffold, we synthesized the corresponding glutarimide-methylated analog, **JHK-02–137** (Figure 5A). Methylation at this position blocks the interaction between the glutarimide moiety and CRBN, thus preventing E3 ligase recruitment, creating a suitable negative control. This CRBN-bump negative control compound failed to induce CDK6 degradation, confirming that the degradation of CDK6 is indeed CRBN-dependent (Figure S18).

**Figure 5:**
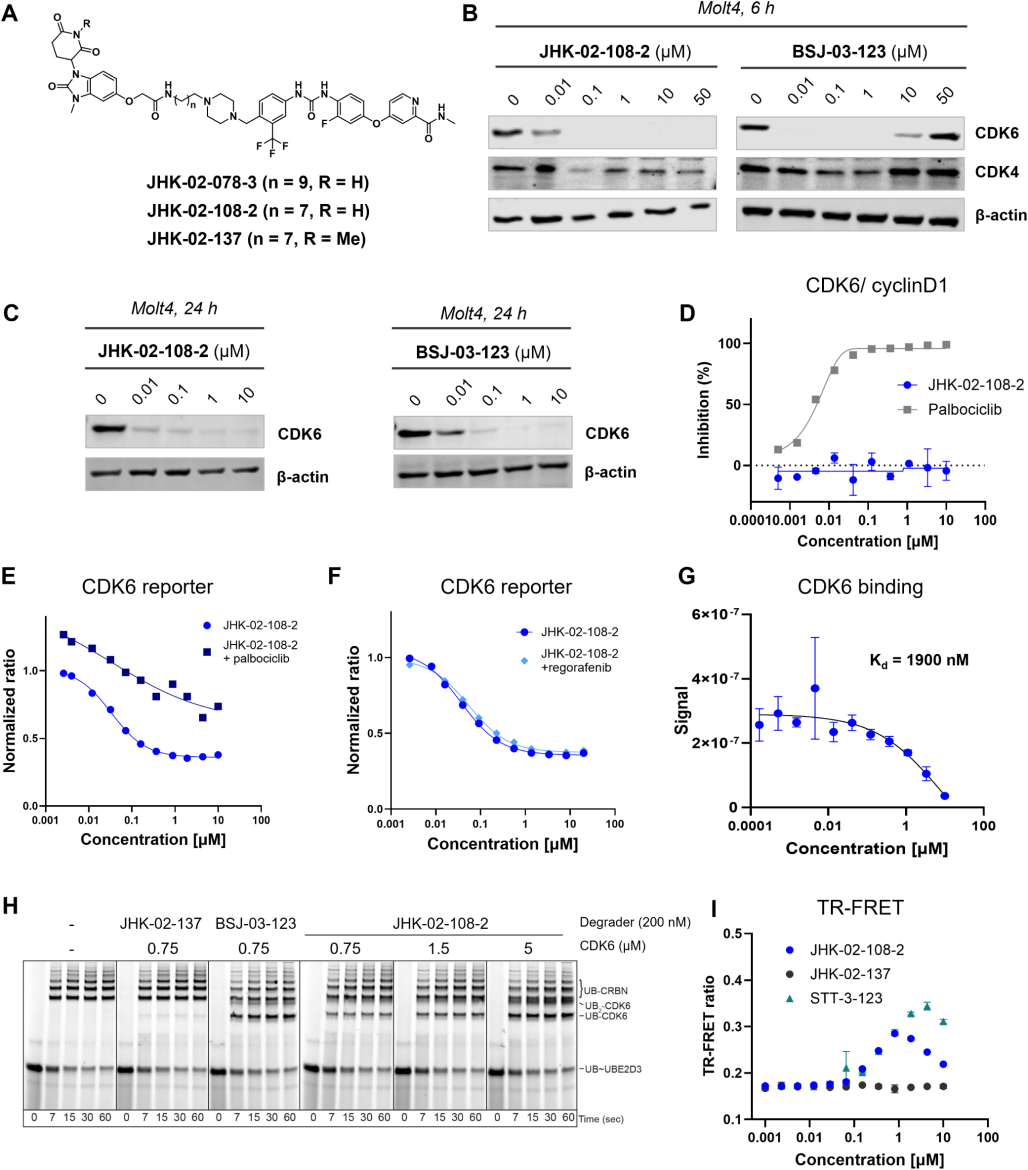
CRBN-dependent CDK6 degradation and ternary complex formation by **JHK-02–108–2.** (A) Chemical structure of type-II CDK6 degraders, **JHK-02–078–3**, **JHK-02–108–2**, and the corresponding negative control, **JHK-02–137**. (B) Western blots showing selective CDK6 degradation compared to CDK4 in MOLT-4 cells after 6 h treatments. (C) Western blots showing the selective degradation of CDK6 following 24-h treatment in MOLT-4 cells. (D) Dose-dependent inhibition of CDK6/cylinD1 activity by **JHK-02–108–2** measured using the Adapta universal kinase assay. Palbociclib was tested as a positive control. (E) Flow cytometry analysis of CDK6_eGFP_ degradation in K562-Cas9 cells treated with **JHK-02–108–2** with or without 2 h pretreatment with palbociclib (1 μM). (F) Flow cytometry analysis of CDK6_eGFP_ degradation in K562-Cas9 cells treated with **JHK-02–108–2** with or without 2 h pretreatment with regorafenib (1 μM). (G) Dose-dependent binding curve of **JHK-02–108–2** to CDK6 as determined by the KINOMEscan KdELECT assay. The binding affinity (K_d_) was calculated from an 11-point 3-fold serial dilution. (H) Pulse-chase ubiquitination assay monitoring the transfer of fluorescently labeled ubiquitin (UB) from the active site of the E2 (UBE2D3) to substrates by incubation with 200 nM of corresponding compounds and neddylated CRL4^CRBN^. Addition of CDK6 shows ubiquitination of CDK6 in addition to automodification of CRBN. (I) Ternary complex formation between CDK6 and CRBN induced by **JHK-02–108–2** and **STT-03–123**, measured by TR-FRET using purified CRBN and CDK6 proteins. **JHK-02–137** does not bind CRBN and fails to induce ternary complex formation.

Our type II-CDK6 degrader is structurally and functionally distinct from the widely studied CDK6 heterobifunctional degrader **BSJ-03–123**, which embeds the CDK6 inhibitor palbociclib and acts via a type I binding mode to induce degradation.^28,29,32^ While both degraders showed selectivity for CDK6 over CDK4, **BSJ-03–123** exhibited a pronounced hook effect at high concentrations, consistent with the formation of excessive binary complexes that outcompete productive ternary complex assembly (Figure 5B and Figure S19). In contrast, no hook effect was observed with **JHK-02–108–2**. The absence of a hook effect can be attributed to the weak intrinsic binding of regorafenib to CDK6 (Figure 2E), suggesting that **JHK-02–108–2** engages CDK6 primarily in the context of the CRBN-mediated ternary complex rather than through strong binary interactions. Because protein turnover rates and degradation kinetics can vary substantially across degraders, we next examined the temporal stability of CDK6 degradation. When the treatment duration was extended to 24 h, the type II CDK6 degrader exhibited more prolonged CDK6 degradation than the palbociclib-based **BSJ-03–123**, underscoring the sustained activity and potential therapeutic advantage of our design (Figure 5C and Figure S20). Especially, the palbociclib-based CDK6 degrader resulted in a rebound in CDK6 protein levels at lower concentrations (10 nM), whereas **JHK-02–108–2** led to a more pronounced degradation of CDK6 than 6 h treatment. No CDK6 inhibitory activity was observed for **JHK-02–108–2** (Figure 5D).

To further test whether target engagement is essential for PROTAC-induced degradation and involves the kinase ATP-binding pocket, we performed a competitive degradation assay using palbociclib, a CDK4 and CDK6 inhibitor that occupies the ATP-binding pocket. Pretreatment of cells with palbociclib rescued **JHK-02–108–2**-induced CDK6 degradation (Figure 5E). In contrast, CDK6 degradation was not affected by regorafenib pretreatment, further supporting that regorafenib itself has minimal binding affinity toward CDK6 (Figure 5F). Measurement of the binary binding affinity between **JHK-02–108–2** and CDK6 yielded a K_d_ of 1900 nM (Figure 5G). While this represents an improvement in CDK6 binding over regorafenib and **STT-03–123** in Figure 2E (both K_d_ > 10,000 nM), the overall affinity remains relatively weak. Interestingly, differences in the structure of the CRBN-recruiting moiety appeared to influence the binary binding affinity toward CDK6.

While the NanoBRET data in Figure 3C suggested ternary complex formation between CDK6 and CRBN, it does not exclude potential contributions from factors such as cell permeability or neosubstrate recruitment without direct binary engagement with CDK6. To directly assess compound-dependent ternary complex formation, we performed a ubiquitination assay and a TR-FRET assay using purified CDK6 and CRBN-DDB1. Both **BSJ-03–123** and **JHK-02–108–2** induced rapid ubiquitination of CDK6, detectable as early as 7 s (Figure 5H). Notably, for **JHK-02–108–2**, ubiquitination increased with higher CDK6 concentrations. Ubiquitination was minimal with the CRBN-bump negative control compound **JHK-02–137**, consistent with CRBN-dependent activity. Consistently, TR-FRET showed that **JHK-02–108–2** induced ternary complex formation between CDK6 and CRBN (EC_50_ = 0.22 μM), whereas no such complex formation was observed with **JHK-02–137** (Figure 5I). Furthermore, a hook effect was observed at higher concentrations (>1 μM) of **JHK-02–108–2**, consistent with its relatively weak binding affinity for CDK6. Notably, similar hook effects at micromolar concentrations have been reported in TR-FRET assays for DCAF16-dependent BRD4 and BRD9 molecular glue degraders.^33–36^ **STT-03–123** (EC_50_ = 0.38 μM) exhibited a similar curve shape to **JHK-02–108–2** but a hook effect was observed at higher concentrations than **JHK-02–108–2**, which may attributed to weaker binary affinity toward CDK6. Together, these results highlight that efficient ternary complex formation can drive CDK6 degradation by **JHK-02–108–2**, even in the context of weak binary binding. This finding aligns with prior studies demonstrating that target engagement or binary binding affinity alone is insufficient to predict degradation outcomes, underscoring the importance of productive ternary complex formation and downstream ubiquitination.^37–40^

### Type II CDK6 Degrader Induces G1 Cell Cycle Arrest in GBM and AML Cells

Type II CDK inhibitors have generally shown limited functional activity in cellular contexts.^13,17,18^ To determine whether targeted degradation could overcome this limitation, we assessed the effects of our type II CDK6 degrader on cell proliferation and cell cycle progression. Recent reports have suggested that CDK6 may be a promising target for a subset of glioblastoma (GBM) with loss of CDKN2A, which encodes the CDK4/6 inhibitor p16^INK4A^.^41–43^ Loss of CDKN2A leads to uncontrolled CDK4/6 activity and makes tumor cells more dependent on CDK6 for proliferation, thereby increasing their sensitivity to CDK6 inhibition or degradation. To evaluate this, we tested **JHK-02–108–2** in BT145 GBM cells harboring CDKN2A loss. BT145 cells were highly sensitive to **JHK-02–108–2,** exhibiting a growth inhibition IC_50_ value of 0.64 μM and dose-dependent G1 arrest compared to the negative control compound **JHK-02–137** (Figure 5A and B). **JHK-02–108–2** induced potent CDK6 degradation (DC_50_ = 11 nM), as determined by immunostaining and microscopy (Figure 5C and D). Considering that **JHK-02–137** shares an otherwise identical structure with **JHK-02–108–2** except for methylation at the CRBN-binding moiety, the kinase inhibition profiles of the two compounds are expected to be largely comparable. Therefore, comparison with the CRBN-bump negative control **JHK-02–137** helps distinguish phenotypes associated with CDK6 degradation from those potentially arising from kinase inhibition. We observed variable responses in other GBM models. While BT179 cells exhibited more pronounced growth inhibition and G1 arrest phenotypes when treated with **JHK-02–108–2** as compared to **JHK-02–137**, three additional lines (BT444, BT187, and BT224) did not show such differences (Figure S21 and Table S3). These results suggest that the contribution of CDK6 degradation to the observed growth inhibition may be context-dependent.

CDK6 has been reported as a critical driver of leukemic proliferation and survival in AML, underscoring its therapeutic significance.^28,44–51^ Accordingly, we tested whether our type II CDK6 degrader could affect cell viability and G1 arrest in AML cell lines. Treatment with JHK-02–108–2 for 72 h resulted in reduced cell proliferation and induction of G1 arrest in U937, NOMO1, and OCIAML2 cells. **JHK-02–108–2** exhibited approximately 2.5–3.9-fold greater growth inhibitory activity than its CRBN-bump negative control, **JHK-02–137**, supporting that these phenotypes are associated with CDK6 degradation (Figure 6E and Table S4). These results are consistent with a contribution of CDK6 degradation to the observed phenotypes (Figure 6F and Figure S22). Given that the type I CDK6 degrader **BSJ-03–123** has been characterized in FLT3-ITD–mutant AML models such as MV4–11,^28^ we also evaluated our degrader in this context. In MV4–11 and MOLM13 cells, **JHK-02–108–2** induced stronger G1 arrest and greater antiproliferative effects than those observed in FLT3-WT AML cell lines (U937, NOMO1, and OCIAML2). However, the separation from its corresponding CRBN-bump negative control, **JHK-02–137**, was less pronounced than in the FLT3-WT AML models (Figure 6E, F and Figure S22). Because MV4–11 and MOLM13 cells harbor FLT3-ITD mutations, they are highly dependent on FLT3 signaling thus the enhanced cytotoxicity in these cell lines may partially arise from unintended FLT3 engagement by both **JHK-02–108–2** and **JHK-02–137**.^49,52,53^ In addition, FLT3-ITD AML has also been reported to exhibit a specific dependency on CDK6 through an FLT3/HCK/CDK6 signaling axis, suggesting that enhanced sensitivity in these models may still be mechanistically linked to CDK6 suppression.^49^ Consistent with this interpretation, we evaluated **JHK-02–145**, in which a piperazine moiety was installed onto the regorafenib scaffold for linker attachment, and found that it exhibited markedly increased cytotoxicity in FLT3-ITD–mutant MV4–11 cells compared with regorafenib, with a 27-fold lower EC_50_ value (Figure S23). Notably, **JHK-02–145** shares structural features with the FLT3 inhibitor AST-487,^54^ supporting the possibility that off-target FLT3 inhibition contributes to the observed phenotype and may partially mask the effects of CDK6 degradation.

**Figure 6:**
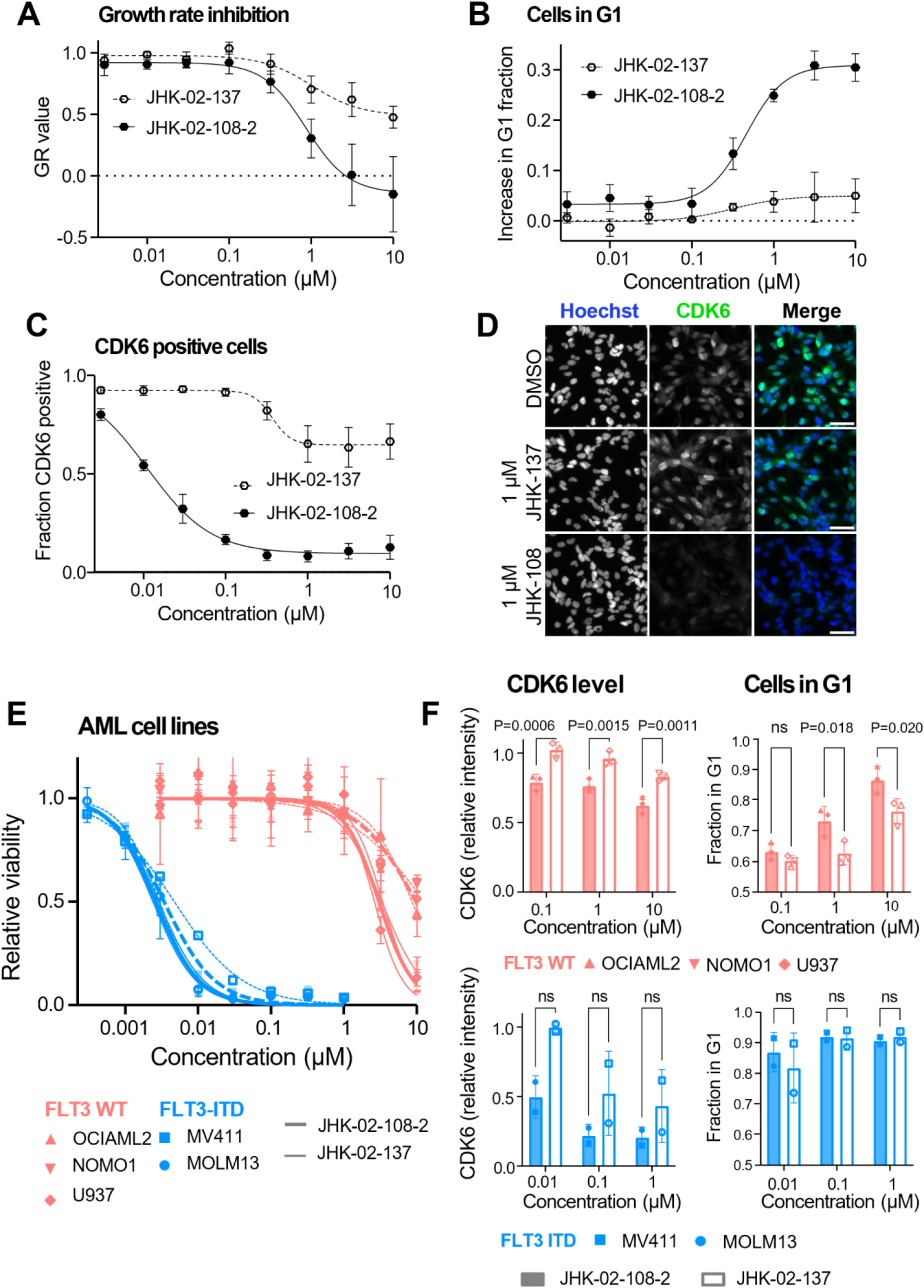
Type II-CDK6 degrader **JHK-02–108–2** induced potent cell cycle arrest in GBM and AML cells. (A) Growth rate inhibition, (B) the fraction of cells in G1, and (C) CDK6 degradation in BT145 cells after 72 h treatments. (D) Confocal microscope imaging of CDK6 degradation after 24 h treatment of 1 μM of corresponding compounds in BT145 cells. Scale bar: 50 μm. (E) Cell viability in FTL3-WT (U937, NOMO1, and OCIAML2) and FLT3-ITD (MV4–11 and MOLM13) AML cells after 72 h treatments. (F) Fraction of CDK6-positive cells and the fraction of cells in FLT3-WT and FLT3-ITD AML cell lines after 72 h treatments. Error bars represent standard deviation of the mean (*n* = 3).

Together, these results in FLT3-WT AML and GBM models indicate that a type II CDK6 degrader can elicit functional cellular effects. Notably, the CRBN-bump negative control **JHK-02–137** also exhibited measurable cytotoxicity, which may be attributable to the intrinsic multikinase activity of the regorafenib-derived warhead. To further investigate this, we performed scanMAX kinome-wide profiling for **JHK-02–108–2**, which revealed engagement with a broad panel of kinases, including FLT3, ABL1, KIT, and FGFR1 (Figure S24 and Tables S5). Although binding affinity does not necessarily correlate with functional inhibition, these data confirm that the warhead retains broad kinase-binding properties. However, despite this promiscuous binding profile, degradation remains selective for CDK6, supporting a partial decoupling between kinase binding and degradation outcomes. At the same time, we cannot exclude that residual kinase inhibition may contribute to the observed cellular phenotypes. Accordingly, comparison with the negative control compound **JHK-02–137** is important for interpreting the contribution of CDK6 degradation. **JHK-02–108–2** exhibited favorable microsomal stability despite its PROTAC-like architecture (Table S6). Taken together, these findings position **JHK-02–108–2** as a chemically tractable example of a type II kinase-based degrader with a hybrid pharmacological profile.

### Overcoming the Challenge of Selective CDK5 Degradation with a Type II Degrader

Developing selective CDK5 degraders has long been considered challenging due to the high structural similarity to CDK2, particularly within the ATP-binding pocket and activation loop.^55^ We previously reported **TMX-2172**, a dual CDK2/5 degrader developed from a CDK1/2/5 inhibitor scaffold.^56^ However, **TMX-2172** was unable to distinguish between CDK2 and CDK5 and binds through a type I binding mode. By exploring diverse CRBN binder ligand structures and linkers, we developed **JHK-02–102–1** as a selective type II CDK5 degrader by employing CRBN binder C and a PEG2 linker. Comparison **of JHK-02–102–1**, **JHK-02–065–2**, and **JHK-02–117**, which share a PEG2 linker and a regorafenib-based warhead, revealed distinct degradation profiles and selectivity (Figures S25–S26). These findings indicate that variations in the CRBN-recruiting ligand can modulate binding orientation and target interactions, thereby influencing ternary complex formation and ultimately driving pronounced differences in degradation profile and selectivity, even with an unchanged type II CDK-binding scaffold.

We validated that the CDK5 degradation is mediated by CRBN recruitment by showing that **JHK-02–138** with a methyl on the glutarimide ring, which is deficient in CRL4^CRBN^ binding, has no activity (Figure 7A). Remarkably, our type-II CDK5 degrader could degrade CDK5 with high potency and selectivity, showing only a very weak hook effect even at a concentration of 50 μM, in contrast to **TMX-2172**, which exhibited a strong hook effect starting at a concentration of 10 μM (Figure 7B–C and Figures S27–S28). The NanoBRET assay profile for **JHK-02–102–1** was sigmoidal, indicating the formation of a ternary complex between CRBN and CDK5 (Figure 7D). In addition, pretreatment of CDK5 reporter cells with MLN4924 prior to **JHK-02–102–1** exposure fully rescued CDK5 levels, supporting that degradation by our type-II CDK5 degrader is mediated through a ubiquitin–proteasome system (UPS)-dependent mechanism (Figure 7E). This highlights that our type-II CDK degrader development strategy successfully overcame the challenge of developing selective CDK5 degraders by enabling their identification through direct degradation-based screening, rather than relying on the selectivity profile of the target-binding warhead.

**Figure 7:**
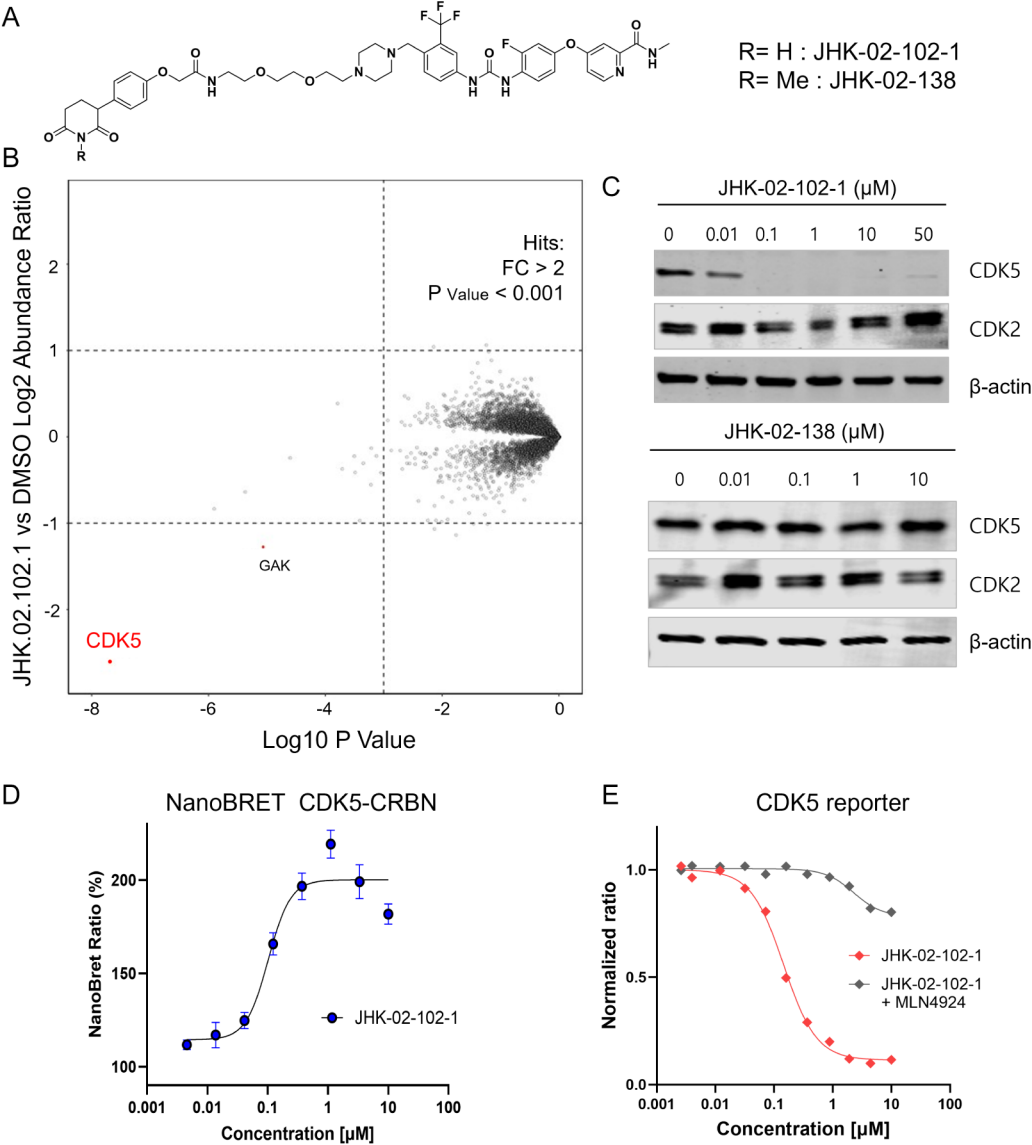
(A) Chemical structure of selective type-II CDK5 degrader, **JHK-02–102–1,** and corresponding negative control **JHK-02–138**. (B) Western blots showing selective CDK5 degradation compared to CDK2 in MOLT-4 cells after 6 h treatments. (C) Volcano plots from global proteomics showing selective CDK5 degradation with **JHK-02–102–1** in MOLT-4 cells treated for 5 h. (D) NanoBRET assay suggesting the induced interaction between HaloTag-CRBN and Nanoluciferase-CDK5 fusion proteins with **JHK-02–102–1**. (E) Flow cytometry analysis of CDK5_eGFP_ degradation in K562-Cas9 cells treated with **JHK-02–102–1** with or without 1 h pretreatment with MLN-4924 (1 μM).

## Conclusion

In this study, we developed regorafenib-derived PROTAC degraders to explore the potential of type II kinase scaffolds in targeted protein degradation. Through proteomics-guided optimization and systematic linker design, we identified **JHK-02–108–2**, a selective type II CDK6 degrader capable of inducing potent and sustained cell-cycle arrest in GBM and AML models. Notably, despite originating from a broad-spectrum kinase inhibitor scaffold, this degrader exhibits selective degradation behavior that diverges from the inhibition profile of the parent inhibitor. Together, these findings establish the first type II inhibitor-derived selective CDK6 degrader and demonstrate that targeted protein degradation can overcome the limitations of type II CDK inhibition to enable functional CDK targeting in cells.

Extending this approach, we also developed **JHK-02–102–1**, a selective type II CDK5 degrader, further illustrating that type II kinase scaffolds can be adapted for selective targeted protein degradation. Our results highlight the importance of E3 ligase engagement, linker architecture, and CRBN binder identity in shaping degradation selectivity, emphasizing that degradation outcomes cannot be predicted solely from inhibitor-binding profiles.

While the degradation profile of **JHK-02–108–2** is selective, residual kinase inhibition from the multikinase inhibitor scaffold may contribute to the observed cellular phenotypes. Ongoing efforts are therefore focused on developing next-generation degraders that retain selective CDK6 degradation while minimizing polypharmacological kinase inhibition. These degraders provide useful chemical tools to investigate the noncatalytic functions of CDK6 and CDK5 and illustrate how targeted protein degradation can modulate kinase biology beyond conventional inhibition-based pharmacology.

## Supplementary Material







## Notes

,^1^The Supporting Information is available free of charge at https://pubs.acs.org/doi/10.1021/jacs.6c10277.

Methods for biology, general information for chemistry, general procedures, ^1^H NMR spectra, and uncropped blots (PDF)
KINOMEscan Profiling of JHK-02–108–2 (CSV)
Raw proteomics data (ZIP)

## ⊗

Aethon Therapeutics, Long Island City, NY, 11101, USA

## ▼

Department of Pharmaceutical Sciences, School of Pharmacy and Pharmaceutical Science, University at Buffalo, 160 Hayes Road, Buffalo, New York, 14214, USA
