# Supplementary material for: A Type II CDK6 Degrader Enables Cellular Targeting beyond the Limits of Type II Inhibition
Source: J Am Chem Soc. 2026 Jun 26;148(26):27951–64. doi: 10.1021/jacs.6c10277 (PMC13352627; doi:10.1021/jacs.6c10277)
Supplement: Supplementary file 1 [file ja6c10277_si_001.pdf]

A Type II CDK6 Degrader Enables Cellular Targeting beyond the Limits of Type II Inhibition

Ji Hyeon Kim[1], Caitlin E. Mills[2], Zuzanna Kozicka[3,4,5], Daniel C. Scott[6], Cyrus Jin[7,8], Zixuan Jiang[1,9] Brendan G. Dwyer[1], Qixiang Geng[1], Sean T. Toenjes[1], Woong Sub Byun[1,10], Dina ElHarouni[11], Hongyu Li[11], Keith L. Ligon[11], Abby M. Thornhill[7], Hannah M. Jones[1], Bryan A. Romero[1], Stephen M. Hinshaw[1], Benjamin L. Ebert[3,4], Brenda A. Schulman[6, 12], Katherine A. Donovan[7,8*], Eric S. Fischer[7,8*], Nathanael S. Gray[1*]

[1]Department of Chemical and Systems Biology, ChEM-H, and Stanford Cancer Institute, Stanford School of Medicine, Stanford University, Stanford, CA, 94305, USA

[2]Laboratory of Systems Pharmacology, Department of Systems Biology, Harvard Medical School, Boston, MA, 02115, USA

[3]Department of Medical Oncology, Dana-Farber Cancer Institute, Boston, MA, 02115, USA

[4]Broad Institute of MIT and Harvard, Cambridge, MA, 02142, USA

[5] ISIC, École Polytechnique Fédérale de Lausanne (EPFL), CH-1015, Switzerland

[6]Department of Structural Biology, St. Jude Children's Research Hospital, Memphis, TN, 38105, USA

[7]Department of Cancer Biology, Dana-Farber Cancer Institute, Boston, MA, 02115, USA

[8]Department of Biological Chemistry and Molecular Pharmacology, Harvard Medical School, Boston, MA, 02115, USA

[9]Department of Chemistry, Stanford School of Humanities and Sciences, Stanford University, Stanford, CA, 94305, USA

[10]College of Pharmacy, Dongguk University-Seoul, Goyang, 10326, Republic of Korea

[11]Department of Pathology, Dana-Farber Cancer Institute, Boston, MA, 02115, USA

[12]Department of Molecular Machines and Signaling, Max Planck Institute of Biochemistry, Martinsried, 82152, Germany

Supplementary information

1. Chemical synthesis

1.1 General methods and materials

Unless otherwise noted, all reagents were purchased from commercial suppliers and used without further purification. Reactions were monitored using a Waters Acquity UPLC/MS system (Waters PDA eλ Detector, QDa Detector, Sample manager - FL, Binary Solvent Manager) using Acquity UPLC® BEH C18 column (2.1 x 50 mm, 1.7 µm particle size): solvent gradient = 85% A at 0 min, 1% A at 1.7 min; solvent A = 0.1% formic acid in water; solvent B = 0.1% formic acid in Acetonitrile; flow rate: 0.6 mL/min. Analytical thin layer chromatography (TLC) was performed on Merck silica gel 60 F254 TLC glass plates and analytes were visualized by fluorescence quenching (using 254 nm light). Purification of reaction products was carried out by flash column chromatography using CombiFlash®Rf with Teledyne Isco RediSep® normal-phase silica flash columns (4 g, 12 g, 24 g, 40 g or 80 g), with Teledyne RediSep Gold ® C18 reversed-phase column (5.5 g, 15.5 g, 30 g, or 50 g), with preparative RP-HPLC using Waters SunFireTM Prep C18 column (19 x 100 mm, 5 µm particle size) with a gradient of 10-90% methanol in water containing 0.035% trifluoroacetic acid (TFA) over 40 min (45 min run time) at a flow of 40 mL/min. NMR spectra were acquired on a 500 MHz Bruker Avance III spectrometer, operating at the denoted spectrometer frequency given in MHz for the specified nucleus. All experiments were acquired at 298.0 K with a calibrated Bruker Variable Temperature Controller unless otherwise noted. The chemical shifts are reported in parts per million (ppm) and coupling constants (J) are given in Hertz (Hz). 1H NMR spectra are reported with the solvent resonance as the reference unless noted otherwise (d_6-DMSO at 2.50 ppm, $CDCl_3$ at 7.26 ppm). Peaks are reported as (s = singlet, d = doublet, t = triplet, q = quartet, m = multiplet or unresolved, br = broad signal, coupling constant(s) in Hz, integration).

1.2 Synthesis procedure

<u>Synthesis of kinase binder</u>

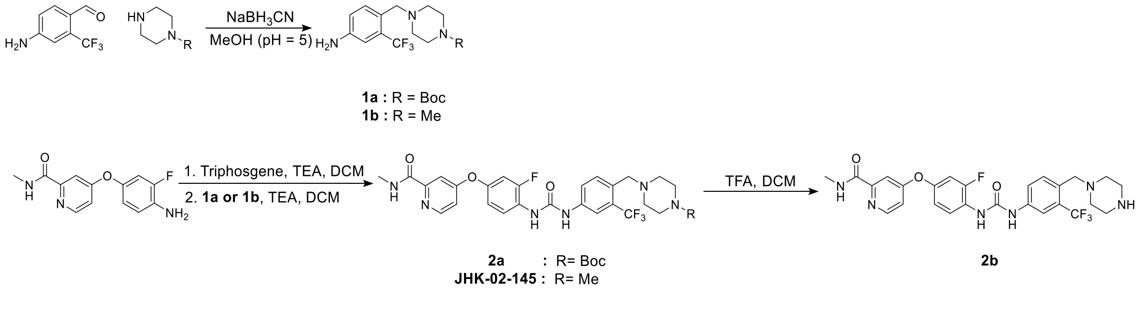

Tert-butyl 4-(4-amino-2-(trifluoromethyl)benzyl)piperazine-1-carboxylate (1a)

To a solution of tert-butyl piperazine-1-carboxylate (449 mg, 2.41 mmol, 1 eq) and 4-amino-2-(trifluoromethyl)benzaldehyde (456 mg, 2.41 mmol, 1 eq) in 5 mL MeOH (pH was adjusted to 5 with acetic acid) was added sodium cyanoborohydride (167 mg, 2.65 mmol, 1.1 eq) and the mixture was stirred at 25 °C for 12 hour. Solvent was removed in vacuo, mixture was dissolved in DCM, and wash with water. Purification through a flash chromatography column with 10% MeOH with DCM afforded tert-butyl 4-(4-amino-2-(trifluoromethyl)benzyl)piperazine-1-carboxylate (1a) (550 mg, 63.5%) as a yellow solid. LC/MS: m/z 360.23 $[M+1]^+$.

4-(3-fluoro-4-(3-(4-(piperazin-1-ylmethyl)-3-(trifluoromethyl)phenyl)ureido)phenoxy)-N-methylpicolinamide (2b):

Triphosgene (842 mg, 3 eq, 2.84 mmol) in dry DCM (10 mL) was added DIPEA (383 mg, 4 eq) dropwise, and stirred at 0 °C for 10 min. Then 4-(4-amino-3-fluorophenoxy)-N-methylpicolinamide (340 mg, 1 eq) was added and the mixture was stirred at 25 °C for 4 h. The solvent was slowly removed in vacuo using a solvent trap containing aqueous sodium hydroxide, then 1-[4-Amino-2-(trifluoromethyl)benzyl]-4-methylpiperazine (1b) (297 mg, 1.1 eq) and DIPEA (383 mg, 4 eq) in dry DCM (10 mL) was added to the mixture, and stirred overnight in a room temperature. The purification was conducted with silica gel chromatography using DCM to 10% MeOH with DCM as a gradient eluent to yield **2a**. LC/MS: m/z 647.31 $[M+1]^+$. **2a** was then dissolved in DCM (33 mL) and subjected to TFA (1 mL) at room temperature for 5 h. The solvent was then evaporated to afford compound **2b** as a yellow solid solid (423 mg, 69% in two steps) as a yellow solid. LC/MS: m/z 547.24 $[M+1]^+$.

4-(3-fluoro-4-(3-(4-((4-methylpiperazin-1-yl)methyl)-3-(trifluoromethyl)phenyl)ureido)phenoxy)-N-methylpicolinamide (JHK-02-145):

Triphosgene (341 mg, 3 eq) in dry DCM (4 mL) was added DIPEA (247 mg, 5 eq) dropwise, stirred at 0 °C for 10 min. Then 4-(4-amino-3-fluorophenoxy)-N-methylpicolinamide (100 mg, 1 eq) was added and the mixture was stirred at 25 °C for 4 h. The solvent was slowly removed in vacuo using a solvent trap containing aqueous sodium hydroxide, then 1-[4-Amino-2-(trifluoromethyl)benzyl]-4-methylpiperazine (**1a**) (105 mg, 1 eq) and DIPEA (247 mg, 5 eq) in dry DCM (4 mL) was added to the mixture, and stirred overnight in a room temperature. The purification was conducted with silica gel chromatography using DCM to 10% MeOH with DCM as a gradient eluent to yield **JHK-02-145** (180 mg, 84%) as a white solid. LC/MS: m/z 561.39 $[M+1]^+$. ^{1}H NMR (500 MHz, DMSO) δ 9.49 (s, 1H), 8.82 (q, *J* = 4.8 Hz, 1H), 8.76 (d, *J* = 2.3 Hz, 1H), 8.54 (d, *J* = 5.6 Hz, 1H), 8.18 (t, *J* = 9.1 Hz, 1H), 8.00 (d, *J* = 2.0 Hz, 1H), 7.67 – 7.59 (m, 2H), 7.42 (d, *J* = 2.6 Hz, 1H), 7.36 (dd, *J* = 11.6, 2.7 Hz, 1H), 7.21 (dd, *J* = 5.6, 2.6 Hz, 1H), 7.08 (ddd, *J* = 9.0, 2.7, 1.2 Hz, 1H), 3.66 (s, 2H), 3.41 (d, *J* = 12.1 Hz, 2H), 3.03 (t, *J* = 11.8 Hz, 2H), 2.93 (d, *J* = 12.5 Hz, 2H), 2.81 (s, 3H), 2.80 (d, *J* = 4.8 Hz, 3H), 2.41 – 2.32 (m, 2H).

General procedure A: Linker attachment to the kinase binder

Synthesis of **Compound 3a :** To a solution of intermediate 2b (30 mg, 1 eq.) and tert-butyl (10-bromodecyl)carbamate (22 mg, 1.2 eq.) in ethanol (12 mL) was potassium carbonate(2 mg, 2 eq), and the solution was stirred at 70 °C overnight. Purification through a reverse phase flash column chromatography with ACN/water to afford the intermediate compound. After dissolving into DCM with 25% TFA, the mixture was stirred under room temperature for 3 h, followed by removing solvents under reduced pressure, yielding **3a** (25 mg, 57% in two steps) as a yellow solid. LC/MS: m/z 702.51 $[M+1]^+$.

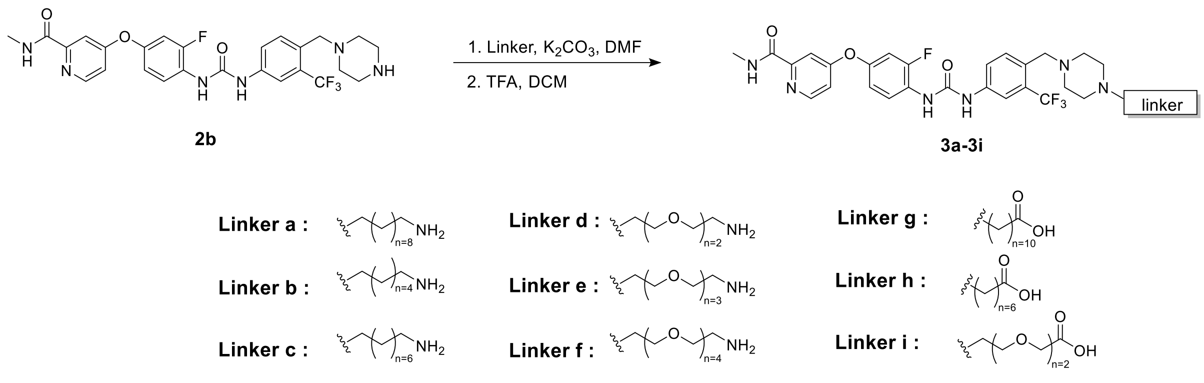

Compound **3b:** The corresponding compound was prepared following **Procedure A** using **2a** (30 mg, 1 eq) to afford **3b** (17 mg, 42% in two steps, LC/MS: m/z 646.31 $[M+1]^+$)

Compound **3c:** The corresponding compound was prepared following **Procedure A** using **2a** (30 mg, 1 eq) to afford **3c** (19 mg, 45% in two steps, LC/MS: m/z 674.46 $[M+1]^+$)

Compound **3d:** The corresponding compound was prepared following **Procedure A** using **2a** (20 mg, 1 eq) to afford **3d** (12 mg, 42% in two steps, LC/MS: m/z 678.35 $[M+1]^+$)

Compound **3e:** The corresponding compound was prepared following **Procedure A** using **2a** (20 mg, 1 eq) to afford **3e** (12 mg, 40% in two steps, LC/MS: m/z 772.40 $[M+1]^+$)

Compound **3f:** The corresponding compound was prepared following **Procedure A** using **2a** (20 mg, 1 eq) to afford **3f** (13 mg, 41% in two steps, LC/MS: m/z 766.40 $[M+1]^+$)

Compound **3g:** The corresponding compound was prepared following **Procedure A** using **2a** (5 mg, 1 eq) to afford **3g** (15 mg, 52% in two steps, LC/MS: m/z 731.36 $[M+1]^+$)

Compound **3h:** The corresponding compound was prepared following **Procedure A** using **2a** (5 mg, 1 eq) to afford **3h** (5 mg, 70% in two steps, LC/MS: m/z 675.29 $[M+1]^+$)

Compound **3i:** The corresponding compound was prepared following **Procedure A** using **2a** (5 mg, 1 eq) to afford **3i** (3 mg, 40% in two steps, LC/MS: m/z 707.69 $[M+1]^+$)

Synthesis of compound 4: To a solution 10-((tert-butoxycarbonyl)amino)decanoic acid (5.3 mg, 1 eq), HATU (14 mg, 2 eq) in DMF (1 mL) was added DIPEA (9.4 μL, 3 eq), and the reaction was stirred at room temperature for 15 min. Intermediate amine **2b** (10 mg, 1eq) was added and the mixture was stirred at room temperature overnight. The crude was then purified by reverse phase combiflash with ACN/H_2O gradient to give the product. The product was dissolved into DCM with 25% TFA, and the mixture was stirred under room temperature for 2 h, followed by removing solvents under reduced pressure yielded **4** as a white solid (10 mg, 76%). LC/MS: m/z 716.40 $[M+1]^+$.

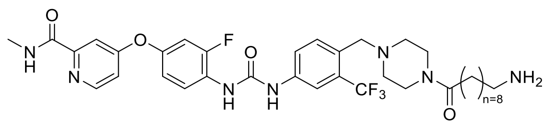

4

<u>**General procedure B**: CRBN binder attachment to generate bivalent degraders 1</u>

Synthesis of **STT-03-123:**

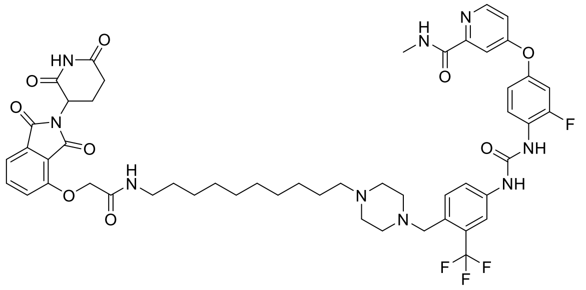

To a solution of 2-((2-(2,6-dioxopiperidin-3-yl)-1,3-dioxoisoindolin-4-yl)oxy)acetic acid (2.0 mg, 1 eq), HATU (4.6 mg, 2 eq) in DMF (0.5 mL) was added DIPEA (3.1 μL, 3 eq), and the reaction was stirred at room temperature for 15 min. Intermediate amine **3a** (4.2 mg, 1eq) was added and the mixture was stirred at room temperature for overnight. The crude was then purified by HPLC with MeOH/H_2O (0.035% TFA) to give compound **STT-03-123** as a white solid (3.2 mg, 52%). LC/MS: m/z 1016.60 $[M+1]^+$. ^{1}H NMR (500 MHz, DMSO) δ 11.14 (s, 1H), 9.47 (s, 1H), 8.82 (q, *J* = 4.8 Hz, 1H), 8.74 (d, *J* = 2.4 Hz, 1H), 8.54 (d, *J* = 5.6 Hz, 1H), 8.18 (t, *J* = 9.0 Hz, 1H), 8.00 (d, *J* = 2.1 Hz, 1H), 7.94 (t, *J* = 5.7 Hz, 1H), 7.82 (dd, *J* = 8.5, 7.3 Hz, 1H), 7.69 – 7.60 (m, 2H), 7.51 (d, *J* = 7.2 Hz, 1H), 7.42 (d, *J* = 2.7 Hz, 1H), 7.40 (d, *J* = 8.5 Hz, 1H), 7.36 (dd, *J* = 11.6, 2.7 Hz, 1H), 7.21 (dd, *J* = 5.6, 2.6 Hz, 1H), 7.08 (ddd, *J* = 8.9, 2.8, 1.2 Hz, 1H), 5.13 (dd, *J* = 12.8, 5.5 Hz, 1H), 4.77 (s, 2H), 3.71 (s, 2H), 3.48 (d, *J* = 12.0 Hz, 2H), 3.20 – 2.85 (m, 10H), 2.80 (d, *J* = 4.9 Hz, 3H), 2.67 – 2.53 (m, 2H), 2.44 (s, 2H), 2.08 – 2.00 (m, 2H), 1.61 (s, 2H), 1.42 (d, *J* = 7.1 Hz, 2H), 1.37 – 1.20 (m, 10H).

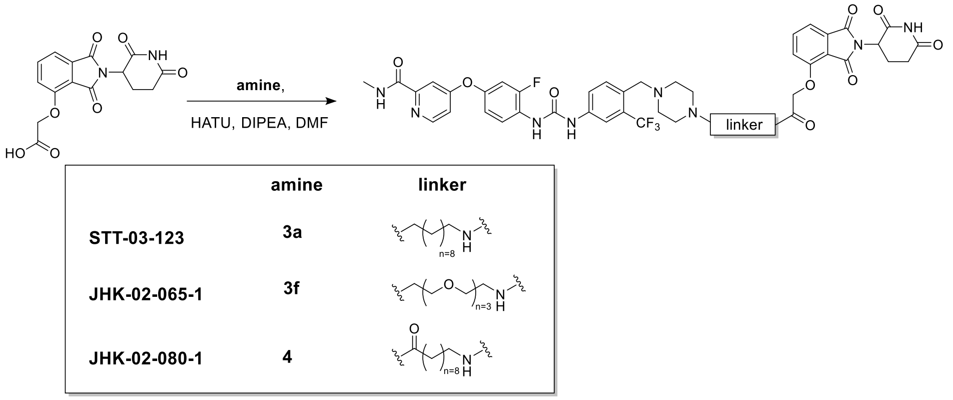

Synthesis of **JHK-02-065-1**:

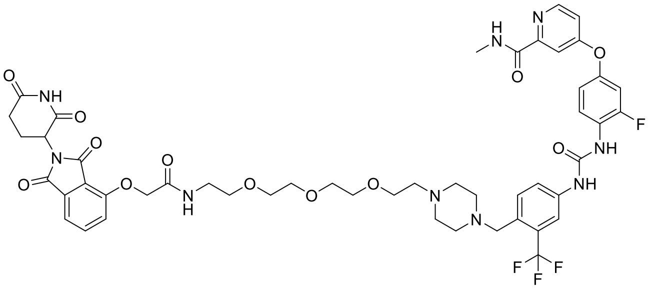

The corresponding compound was prepared following **Procedure B** using 2-((2-(2,6-dioxopiperidin-3-

yl)-1,3-dioxoisoindolin-4-yl)oxy)acetic acid (2.0 mg, 6 µmol, 1.1 eq.) and intermediate **3e** (1 eq). Purification by HPLC with MeOH/H_2O (0.035% TFA) to afford the product as a white solid (2.3 mg, 40%). LC/MS: m/z 1036.26 $[M+1]^+$. ^1H NMR (500 MHz, DMSO) δ 11.12 (s, 1H), 9.52 (s, 1H), 8.78 (dd, *J* = 7.6, 3.6 Hz, 2H), 8.54 (d, *J* = 5.6 Hz, 1H), 8.17 (t, *J* = 9.1 Hz, 1H), 7.99 (d, *J* = 2.0 Hz, 2H), 7.81 (dd, *J* = 8.5, 7.3 Hz, 1H), 7.68 – 7.60 (m, 2H), 7.51 (d, *J* = 7.2 Hz, 1H), 7.42 (d, *J* = 2.7 Hz, 1H), 7.41 (d, *J* = 8.6 Hz, 1H), 7.34 (dd, *J* = 11.6, 2.7 Hz, 1H), 7.20 (dd, *J* = 5.6, 2.6 Hz, 1H), 7.07 (ddd, *J* = 8.9, 2.7, 1.2 Hz, 1H), 5.12 (dd, *J* = 12.8, 5.4 Hz, 1H), 4.79 (s, 2H), 3.74 (t, *J* = 5.0 Hz, 2H), 3.65 (s, 2H), 3.59 – 3.43 (m, 12H), 3.36 – 3.31 (m, 4H), 3.08 (m, 2H), 2.90 (m, 3H), 2.80 (d, *J* = 4.8 Hz, 3H), 2.60 (d, *J* = 18.9 Hz, 2H), 2.46 (m, 2H), 2.11 – 2.00 (m, 1H).

Synthesis of **JHK-02-080-1**:

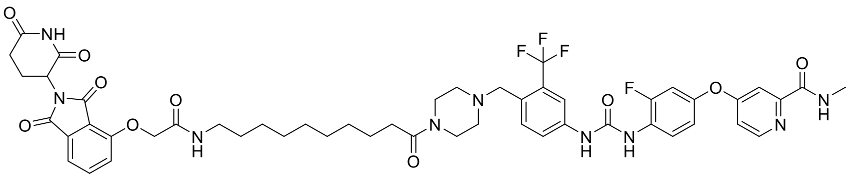

The corresponding compound was prepared following **Procedure B** using 2-((2-(2,6-dioxopiperidin-3-yl)-1,3-dioxoisoindolin-4-yl)oxy)acetic acid (2.0 mg, 6 µmol, 1 eq.) and intermediate **4** (1 eq). Purification by HPLC with MeOH/H_2O (0.035% TFA) to afford the product as a white solid (1.3 mg, 20%). LC/MS: m/z 1030.67 $[M+1]^+$. ^1H NMR (500 MHz, DMSO) δ 11.12 (s, 1H), 9.72 (s, 1H), 8.88 (s, 1H), 8.79 (q, *J* = 4.8 Hz, 1H), 8.54 (d, *J* = 5.6 Hz, 1H), 8.15 (t, *J* = 9.0 Hz, 1H), 8.10 (s, 1H), 7.93 (t, *J* = 5.8 Hz, 1H), 7.86 – 7.73 (m, 3H), 7.51 (d, *J* = 7.2 Hz, 1H), 7.44 – 7.39 (m, 2H), 7.35 (dd, *J* = 11.5, 2.7 Hz, 1H), 7.20 (dd, *J* = 5.6, 2.6 Hz, 1H), 7.09 (dd, *J* = 9.2, 2.6 Hz, 1H), 5.12 (dd, *J* = 12.8, 5.4 Hz, 1H), 4.77 (s, 2H), 4.32 (s, 2H), 3.4 –3.6 (2H hidden by H2O peak), 3.20 – 2.86 (m, 8H), 2.80 (d, *J* = 4.9 Hz, 3H), 2.65 – 2.53 (m, 2H), 2.33 (t, *J* = 7.5 Hz, 2H), 2.05 (ddd, *J* = 11.5, 6.2, 3.8 Hz, 1H), 1.46 (dt, *J* = 23.4, 7.0 Hz, 4H), 1.26 (d, *J* = 3.4 Hz, 10H).

Synthesis of 2-((2-(2,6-dioxopiperidin-3-yl)-1,3-dioxoisoindolin-5-yl)oxy)acetic acid (**JHK-02-071)**:

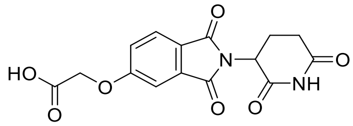

To a solution of I, III, IV in THF (5 mL) was cooled to icebath and added diisopropyl (E)-diazene-1,2-dicarboxylate (442 mg, 1.2 Eq, 2.19 mmol) under N2 atmosphere and the mixture was stirred at 25 °C for 12 h. Product was purified through silica gel chromatography yielding tert-butyl 2-((2-(2,6-dioxopiperidin-3-yl)-1,3-dioxoisoindolin-5-yl)oxy)acetate (345 mg, 49%). Subsequent deprotection of the t-butyl group was performed by 30% TFA in DCM for 12 h to yield JHK-02-071 (270 mg, 91%). LC/MS: m/z 333.01 $[M+1]^+$.

Synthesis of **JHK-02-078-1**:

The corresponding compound was prepared following **Procedure B** using 2-((2-(2,6-dioxopiperidin-3-yl)-1,3-dioxoisoindolin-5-yl)oxy)acetic acid (**JHK-02-071,** 3.0 mg, 1.1 eq.) and intermediate **3a** (1 eq). Purification by HPLC with MeOH/H_2O (0.035% TFA) to afford the product as a white solid (1.8 mg, 20%). LC/MS: m/z 1016.38 $[M+1]^+$.

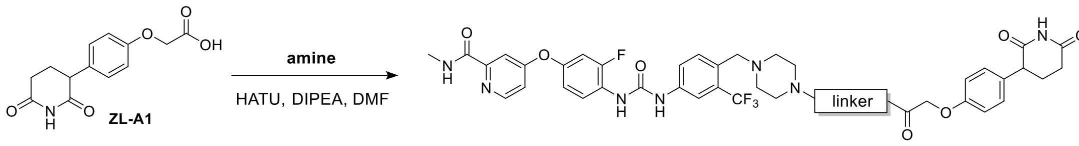

	amine	linker
JHK-02-078-2	3a	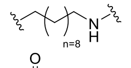
JHK-02-080-2	4	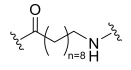
JHK-02-102-1	3d	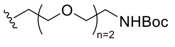

Synthesis of **JHK-02-078-2**:

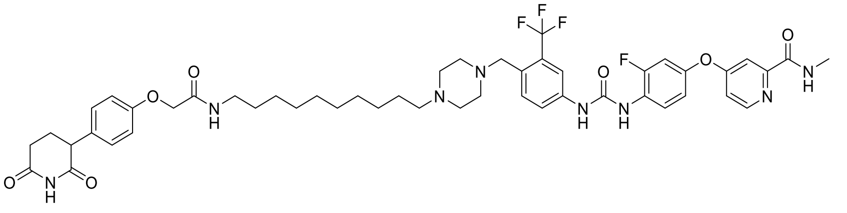

The corresponding compound was prepared following **Procedure B** using 2-(4-(2,6-dioxopiperidin-3-yl)phenoxy)acetic acid (**ZL-A1,** 3.0 mg, 1.1 eq.) and intermediate **3a** (1 eq). Purification by HPLC with MeOH/H_2O (0.035% TFA) to afford the product as a white solid (6 mg, 60%). LC/MS: m/z 947.48 $[M+1]^+$. ^{1}H NMR (500 MHz, DMSO) δ 10.80 (s, 1H), 9.48 (d, *J* = 2.4 Hz, 1H), 8.82 (d, *J* = 4.9 Hz, 1H), 8.75 (dr, *J* = 2.3 Hz, 1H), 8.53 (d, *J* = 5.6 Hz, 1H), 8.14 (t, *J* = 9.0 Hz, 1H), 8.10 (t, *J* = 6.0 Hz, 1H), 7.99 (s, 1H), 7.66 – 7.59 (m, 2H), 7.41 (d, *J* = 2.6 Hz, 1H), 7.33 (dt, *J* = 11.5, 2.2 Hz, 1H), 7.19 (dd, *J* = 5.6, 2.6 Hz, 1H), 7.16 – 7.12 (m, 2H), 7.10 – 7.05 (m, 1H), 6.95 – 6.87 (m, 2H), 4.43 (s, 2H), 3.77 (m, 6H), 3.15 – 2.85 (m, 8H), 2.79 (d, *J* = 4.8 Hz, 3H), 2.65 (ddd, *J* = 17.1, 11.8, 5.3 Hz, 1H), 2.48 – 2.30 (m, 2H), 2.23 – 2.08 (m, 1H), 2.05 – 1.94 (m, 1H), 1.60 (m, 2H), 1.41 (t, *J* = 7.0 Hz, 2H), 1.25 (m, 12H).

Synthesis of **JHK-02-080-2**:

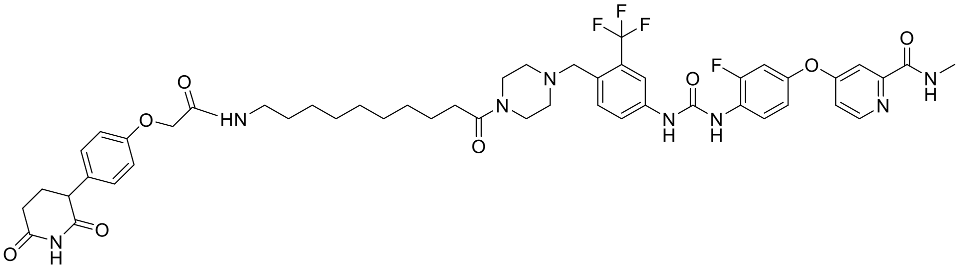

The corresponding compound was prepared following **Procedure B** using 2-(4-(2,6-dioxopiperidin-3-yl)phenoxy)acetic acid (**ZL-A1,** 2.0 mg, 1 eq.) and intermediate **4** (4 mg, 1 eq). Purification by HPLC with MeOH/H_2O (0.035% TFA) to afford the product as a white solid (1.5 mg, 30%). LC/MS: m/z 961.46 $[M+1]^+$. ^{1}H NMR (500 MHz, DMSO) δ 10.79 (s, 1H), 9.41 (s, 1H), 8.79 (q, *J* = 4.8 Hz, 1H), 8.70

(s, 1H), 8.54 (d, *J* = 5.6 Hz, 1H), 8.20 (t, *J* = 9.1 Hz, 1H), 8.03 (dd, *J* = 13.3, 7.5 Hz, 2H), 7.69 (d, *J* = 8.5 Hz, 1H), 7.58 (d, *J* = 8.4 Hz, 1H), 7.43 (d, *J* = 2.6 Hz, 1H), 7.34 (dd, *J* = 11.6, 2.7 Hz, 1H), 7.19 (dd, *J* = 5.6, 2.6 Hz, 1H), 7.17 – 7.13 (m, 2H), 7.11 – 7.05 (m, 1H), 6.97 – 6.90 (m, 2H), 4.44 (s, 2H), 3.80 (dd, *J* = 11.5, 4.9 Hz, 1H), 3.57 (s, 1H), 3.45 (s, 3H), 3.12 (q, *J* = 6.7 Hz, 2H), 2.80 (d, *J* = 4.8 Hz, 3H), 2.66 (ddd, *J* = 17.1, 11.7, 5.2 Hz, 1H), 2.38 – 2.25 (m, 5H), 2.16 (qd, *J* = 11.9, 4.4 Hz, 1H), 2.01 (td, *J* = 9.1, 4.1 Hz, 2H), 1.49 – 1.39 (m, 4H), 1.25 (s, 14H).

Synthesis of **JHK-02-102-1**:

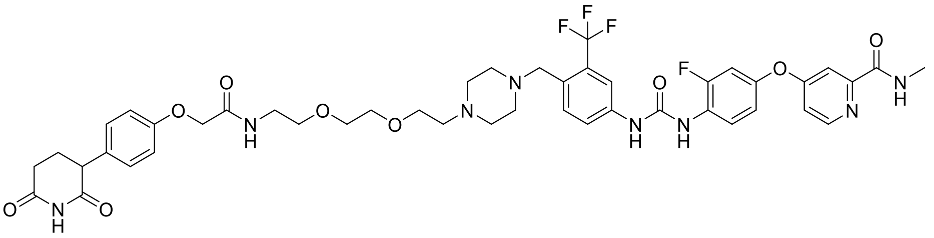

The corresponding compound was prepared following **Procedure B** using 2-(4-(2,6-dioxopiperidin-3-yl)phenoxy)acetic acid (**ZL-A1,** 5.4 mg, 1 eq.) and intermediate **3d** (14 mg, 1 eq). Purification by HPLC with MeOH/H_2O (0.035% TFA) to afford the product as a white solid (3 mg, 16%). LC/MS: m/z 923.35 $[M+1]^+$. ^{1}H NMR (500 MHz, DMSO) δ 10.80 (s, 1H), 9.51 (s, 1H), 8.78 (dt, J = 12.2, 3.5 Hz, 2H), 8.54 (d, J = 5.6 Hz, 1H), 8.17 (t, J = 9.1 Hz, 1H), 8.08 (t, J = 5.8 Hz, 1H), 7.99 (d, J = 1.9 Hz, 1H), 7.67 – 7.59 (m, 2H), 7.42 (d, J = 2.6 Hz, 1H), 7.34 (dd, J = 11.6, 2.7 Hz, 1H), 7.20 (dd, J = 5.6, 2.6 Hz, 1H), 7.19 – 7.13 (m, 2H), 7.10 – 7.05 (m, 1H), 6.95 – 6.88 (m, 2H), 4.47 (s, 2H), 3.79 (dd, J = 11.6, 4.9 Hz, 2H), 3.74 (m, 6H), 3.59 – 3.51 (m, 8H), 3.47 (t, J = 6.0 Hz, 4H), 3.31 (q, J = 5.9 Hz, 4H), 3.08 (s, 2H), 2.91 (s, 2H), 2.80 (d, J = 4.9 Hz, 3H), 2.65 (ddd, J = 17.1, 11.8, 5.3 Hz, 1H), 2.50 (t, *J* = 4.2 Hz, 1H), 2.16 (qd, J = 12.1, 4.5 Hz, 1H), 2.00 (dq, J = 13.3, 4.7 Hz, 1H).

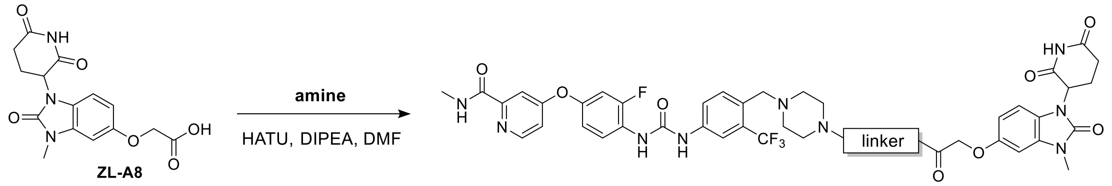

	amine	linker		amine	linker
**JHK-02-078-3**	**3a**	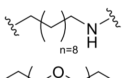	**JHK-02-104-2**	**3f**	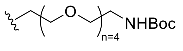
**JHK-02-102-2**	**3d**	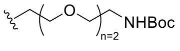	**JHK-02-108-2**	**3c**	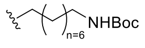
**JHK-02-103-2**	**3e**	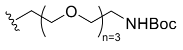			

Synthesis of **JHK-02-078-3**:

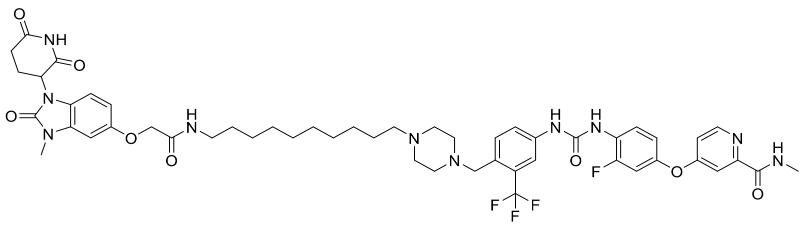

The corresponding compound was prepared following **Procedure B** using 2-((1-(2,6-dioxopiperidin-3-yl)-3-methyl-2-oxo-2,3-dihydro-1H-benzo[d]imidazol-5-yl)oxy)acetic acid (**ZL-A8**, 3.0 mg, 10 µmol, 1.1 eq.) and intermediate **3a** (1 eq). Purification by HPLC with MeOH/H_2O (0.035% TFA) to afford the product as a white solid (3.5 mg, 30%). LC/MS: m/z 1016.38 $[M+1]^+$. ^1H NMR (500 MHz, DMSO) δ 11.03 (s, 1H), 9.39 (s, 1H), 9.14 (s, 1H), 8.75 (q, *J* = 4.8 Hz, 1H), 8.66 (d, f*J* = 2.3 Hz, 1H), 8.47 (d, *J* = 5.6 Hz, 1H), 8.11 (t, *J* = 9.1 Hz, 1H), 7.99 (t, *J* = 5.9 Hz, 1H), 7.93 (d, *J* = 2.2 Hz, 1H), 7.63 – 7.54 (m, 2H), 7.35 (d, *J* = 2.6 Hz, 1H), 7.29 (dd, *J* = 11.6, 2.7 Hz, 1H), 7.14 (dd, *J* = 5.6, 2.7 Hz, 1H), 7.01 (ddd, *J* = 8.9, 2.7, 1.3 Hz, 1H), 6.95 (d, *J* = 8.6 Hz, 1H), 6.85 (d, *J* = 2.4 Hz, 1H), 6.60 (dd, *J* = 8.6, 2.4 Hz, 1H), 5.26 (dd, *J* = 12.9, 5.4 Hz, 1H), 4.39 (s, 2H), 3.65 (s, 2H), 3.41 (d, *J* = 11.8 Hz, 2H), 3.24 (s, 3H), 3.10 – 2.97 (m, 3H), 2.97 – 2.78 (m, 4H), 2.72 (d, *J* = 4.8 Hz, 5H), 2.69 – 2.49 (m, 2H), 2.38 (m, 2H), 1.92 (dtd, *J* = 10.8, 5.6, 2.9 Hz, 1H), 1.54 (m, 2H), 1.36 (m, 2H), 1.27 – 1.04 (m, 8H).

Synthesis of **JHK-02-108-2**:

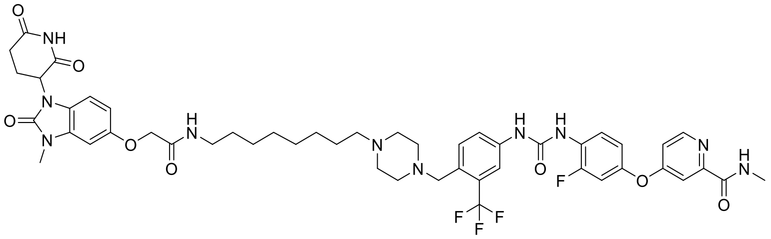

The corresponding compound was prepared following **Procedure B** using 2-((1-(2,6-dioxopiperidin-3-yl)-3-methyl-2-oxo-2,3-dihydro-1H-benzo[d]imidazol-5-yl)oxy)acetic acid (**ZL-A8**, 23 mg, 1.1 eq.) and intermediate **3c** (43 mg, 1 eq). Purification by HPLC with MeOH/H_2O (0.035% TFA) to afford the product as a white solid (11 mg, 17%). LC/MS: m/z 989.56 $[M+1]^+$. ^1H NMR (500 MHz, DMSO) δ 11.09 (s, 1H), 9.51 (s, 1H), 8.83 – 8.75 (m, 2H), 8.54 (d, *J* = 5.6 Hz, 1H), 8.18 (d, *J* = 9.0 Hz, 1H), 8.06 (t, *J* = 5.9 Hz, 1H), 8.00 (d, *J* = 2.0 Hz, 1H), 7.72 – 7.60 (m, 2H), 7.42 (d, *J* = 2.6 Hz, 1H), 7.34 (dd, *J* = 11.6, 2.7 Hz, 1H), 7.20 (dd, *J* = 5.6, 2.6 Hz, 1H), 7.08 (ddd, *J* = 8.9, 2.8, 1.3 Hz, 1H), 7.02 (d, *J* = 8.6 Hz, 1H), 6.92 (d, *J* = 2.4 Hz, 1H), 6.68 (dd, *J* = 8.6, 2.4 Hz, 1H), 5.33 (dd, *J* = 12.9, 5.5 Hz, 1H), 4.46 (s, 2H), 3.47 (m, *J* = 5.6 Hz, 3H), 3.32 (s, 3H), 3.13 (q, *J* = 6.7 Hz, 2H), 3.06 (dd, *J* = 9.9, 6.4 Hz, 2H), 3.03 – 2.86 (m, 5H), 2.80 (d, *J* = 4.8 Hz, 3H), 2.72 – 2.60 (m, 2H), 2.42 (s, 2H), 2.07 – 1.95 (m, 1H), 1.61 (s, 2H), 1.47 – 1.42 (m, 2H), 1.26 (s, 9H).

Synthesis of **JHK-02-102-2**:

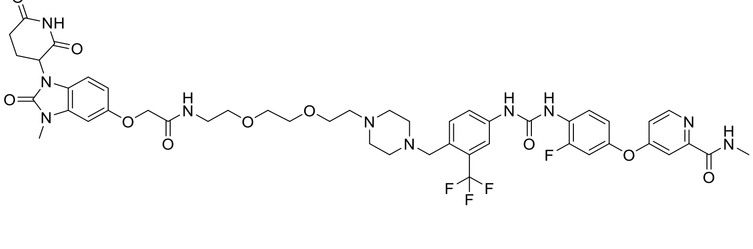

The corresponding compound was prepared following **Procedure B** using 2-((1-(2,6-dioxopiperidin-3-yl)-3-methyl-2-oxo-2,3-dihydro-1H-benzo[d]imidazol-5-yl)oxy)acetic acid (**ZL-A8**, 6.9 mg, 1 eq.) and intermediate **3d** (14 mg, 1 eq). Purification by HPLC with MeOH/H_2O (0.035% TFA) to afford the product as a white solid (4.6 mg, 23%). LC/MS: m/z 993.65 $[M+1]^+$. ^{1}H NMR (500 MHz, DMSO) δ 11.08 (s, 1H), 9.51 (s, 1H), 8.79 (q, *J* = 5.1 Hz, 1H), 8.77 (d, *J* = 2.3 Hz, 1H), 8.54 (d, *J* = 5.6 Hz, 1H), 8.17 (t, *J* = 9.0 Hz, 1H), 8.07 (t, *J* = 5.9 Hz, 1H), 7.99 (d, *J* = 1.9 Hz, 1H), 7.63 (d, *J* = 2.7 Hz, 2H), 7.42 (d, *J* = 2.6 Hz, 1H), 7.34 (dd, *J* = 11.6, 2.7 Hz, 1H), 7.20 (dd, *J* = 5.6, 2.6 Hz, 1H), 7.08 (dt, *J* = 8.9, 1.8 Hz, 1H), 7.02 (d, *J* = 8.6 Hz, 1H), 6.92 (d, *J* = 2.4 Hz, 1H), 6.68 (dd, *J* = 8.6, 2.4 Hz, 1H), 5.33 (dd, *J* = 12.9, 5.4 Hz, 1H), 4.48 (s, 2H), 3.74 (t, *J* = 5.0 Hz, 2H), 3.67 (s, 2H), 3.56 (tt, *J* = 5.4, 2.6 Hz, 4H), 3.48 (t, *J* = 6.1 Hz, 2H), 3.31 (d, *J* = 4.9 Hz, 8H), 3.08 (s, 2H), 2.90 (ddd, *J* = 17.7, 13.2, 5.4 Hz, 3H), 2.80 (d, *J* = 4.8 Hz, 3H), 2.75 – 2.54 (m, 2H), 2.03 – 1.95 (m, 1H).

Synthesis of **JHK-02-103-2**:

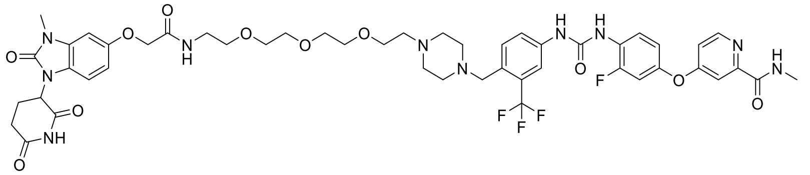

The corresponding compound was prepared following **Procedure B** using 2-((1-(2,6-dioxopiperidin-3-yl)-3-methyl-2-oxo-2,3-dihydro-1H-benzo[d]imidazol-5-yl)oxy)acetic acid (**ZL-A8**, 4.4 mg, 1 eq) and intermediate **3e** (12 mg, 1 eq). Purification by HPLC with MeOH/H_2O (0.035% TFA) to afford the product as a white solid (5 mg, 29%). LC/MS: m/z 1037.73 $[M+1]^+$. ^{1}H NMR (500 MHz, DMSO) δ 11.08 (s, 1H), 9.52 (s, 1H), 8.83 – 8.73 (m, 2H), 8.54 (d, *J* = 5.6 Hz, 1H), 8.17 (t, *J* = 9.0 Hz, 1H), 8.06 (t, *J* = 5.8 Hz, 1H), 8.00 (d, *J* = 2.0 Hz, 1H), 7.68 – 7.60 (m, 2H), 7.42 (d, *J* = 2.6 Hz, 1H), 7.34 (dd, *J* = 11.6, 2.7 Hz, 1H), 7.20 (dd, *J* = 5.6, 2.6 Hz, 1H), 7.10 – 7.06 (m, 1H), 7.02 (d, *J* = 8.6 Hz, 1H), 6.92 (d, *J* = 2.4 Hz, 1H), 6.68 (dd, *J* = 8.6, 2.4 Hz, 1H), 5.33 (dd, *J* = 12.8, 5.4 Hz, 1H), 4.48 (s, 2H), 3.74 (t, *J* = 5.1 Hz, 2H), 3.67 (s, 2H), 3.61 – 3.53 (m, 5H), 3.51 (s, 4H), 3.46 (t, *J* = 6.1 Hz, 3H), 3.31 (m, *J* = 6.4 Hz, 7H), 3.08 (s, 2H), 2.90 (ddd, *J* = 18.9, 11.3, 3.5 Hz, 3H), 2.80 (d, *J* = 4.8 Hz, 3H), 2.74 – 2.59 (m, 2H), 2.47 (brd s, 2H), 2.05 – 1.93 (m, 1H).

Synthesis of **JHK-02-104-2**:

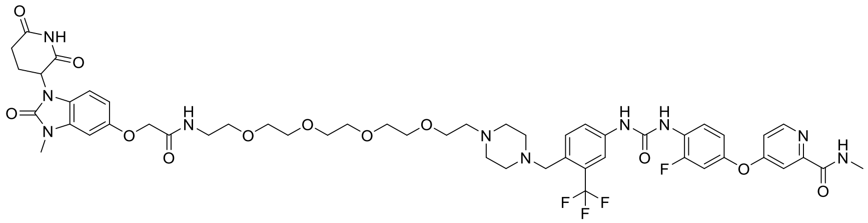

The corresponding compound was prepared following **Procedure B** using 2-((1-(2,6-dioxopiperidin-3-yl)-3-methyl-2-oxo-2,3-dihydro-1H-benzo[d]imidazol-5-yl)oxy)acetic acid (**ZL-A8**, 4.5 mg, 1 eq.) and intermediate **3f** (10 mg, 1 eq). Purification by HPLC with MeOH/H_2O (0.035% TFA) to afford the product as a white solid (4 mg, 28%). LC/MS: m/z 1081.69 $[M+1]^+$. ^{1}H NMR (500 MHz, DMSO) δ 11.08 (s, 1H), 9.53 (s, 1H), 8.79 (t, *J* = 4.2 Hz, 2H), 8.54 (d, *J* = 5.6 Hz, 1H), 8.17 (t, *J* = 9.0 Hz, 1H), 8.05 (t, *J* = 5.8 Hz, 1H), 8.00 (d, *J* = 2.0 Hz, 1H), 7.68 – 7.59 (m, 2H), 7.42 (d, *J* = 2.6 Hz, 1H), 7.34 (dd, *J* = 11.6, 2.7 Hz, 1H), 7.20 (dd, *J* = 5.6, 2.6 Hz, 1H), 7.11 – 7.06 (m, 1H), 7.02 (d, *J* = 8.7 Hz, 1H), 6.92 (d, *J* = 2.4 Hz, 1H), 6.68 (dd, *J* = 8.7, 2.4 Hz, 1H), 5.33 (dd, *J* = 12.9, 5.4 Hz, 1H), 4.48 (s, 2H), 3.74 (t, *J* = 5.0 Hz,

2H), 3.67 (s, 2H), 3.60 – 3.54 (m, 5H), 3.49 (m, 4H), 3.48 (s, 4H), 3.46 (t, J = 6.1 Hz, 3H), 3.32 (d, J = 2.1 Hz, 7H), 3.09 (s, 2H), 2.96 – 2.85 (m, 3H), 2.80 (d, J = 4.8 Hz, 3H), 2.74 – 2.60 (m, 2H), 2.47 (brd s, 2H), 2.00 (m, 1H).

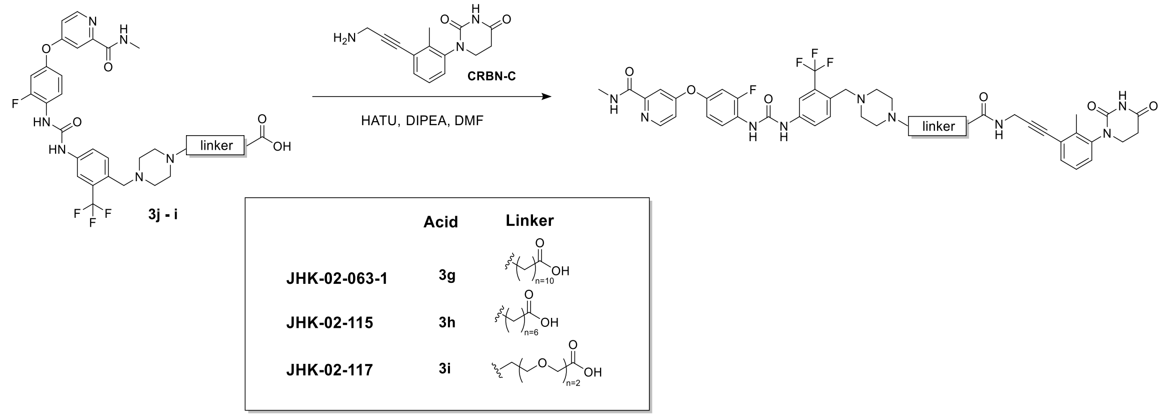

General Procedure C: <u>CRBN binder attachment to generate bivalent degraders 2</u>

Synthesis of **JHK-02-063-1**:

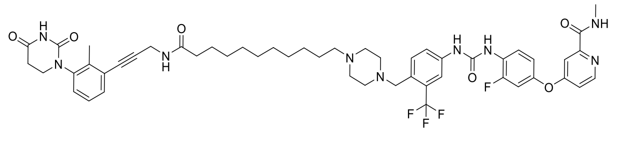

To a solution of compound **3i** (6.0 mg, 1 eq), HATU (6 mg, 2 eq) in DMF (1 mL) was added DIPEA (4 μL, 3 eq), and the reaction was stirred at room temperature for 15 min. Intermediate amine **CRBN-C** (2 mg, 1.1 eq) was added and the mixture was stirred at room temperature overnight. Purification by HPLC with MeOH/H_2O (0.035% TFA) to afford the product as a white solid (2.4 mg, 30%). LC/MS: m/z 970.46 $[M+1]^+$.^{1}H NMR (500 MHz, DMSO) δ 10.38 (s, 1H), 9.50 (s, 1H), 8.79 (q, J = 5.0 Hz, 1H), 8.77 (d, J = 2.3 Hz, 1H), 8.54 (d, J = 5.6 Hz, 1H), 8.35 (t, J = 5.5 Hz, 1H), 8.16 (t, J = 9.1 Hz, 1H), 7.99 (d, J = 2.0 Hz, 1H), 7.68 – 7.59 (m, 2H), 7.42 (d, J = 2.6 Hz, 1H), 7.37 – 7.28 (m, 3H), 7.24 (t, J = 7.7 Hz, 1H), 7.20 (dd, J = 5.6, 2.6 Hz, 1H), 7.08 (ddd, J = 9.0, 2.8, 1.3 Hz, 1H), 4.14 (d, J = 5.5 Hz, 2H), 3.78 (ddd, J = 12.3, 9.8, 5.2 Hz, 1H), 3.66 (s, 2H), 3.11 – 3.03 (m, 2H), 2.98 (d, J = 7.6 Hz, 2H), 2.95 – 2.89 (m, 2H), 2.83 – 2.75 (m, 5H), 2.69 (dt, J = 16.7, 5.5 Hz, 1H), 2.40 (t, J = 12.6 Hz, 2H), 2.24 (s, 3H), 2.11 (t, J = 7.3 Hz, 2H), 1.60 (s, 2H), 1.51 (t, J = 7.1 Hz, 2H), 1.26 (d, J = 5.4 Hz, 14H).

Synthesis of **JHK-02-115**:

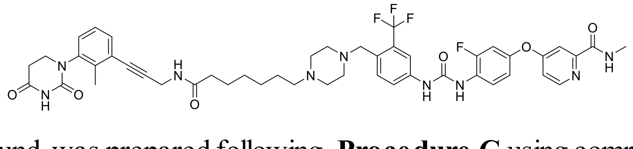

The corresponding compound was prepared following **Procedure C** using compound **3h** (5 mg, 1 eq) Purification by HPLC with MeOH/H_2O (0.035% TFA) to afford the product as a white solid (4.5 mg,

70%). LC/MS: m/z 914.62 [M+1]$^{+}$. ^{1}H NMR (500 MHz, DMSO) δ 10.39 (s, 1H), 9.58 (s, 1H), 8.80 (d, *J* = 5.4 Hz, 2H), 8.54 (d, *J* = 5.6 Hz, 1H), 8.37 (t, *J* = 5.6 Hz, 1H), 8.17 (t, *J* = 9.0 Hz, 1H), 8.00 (d, *J* = 2.0 Hz, 1H), 7.64 (d, *J* = 3.9 Hz, 2H), 7.42 (d, *J* = 2.6 Hz, 1H), 7.38 – 7.30 (m, 3H), 7.24 (t, *J* = 7.7 Hz, 1H), 7.20 (dd, *J* = 5.6, 2.6 Hz, 1H), 7.10 – 7.06 (m, 1H), 4.15 (d, *J* = 5.2 Hz, 2H), 3.78 (ddd, *J* = 12.1, 9.8, 5.1 Hz, 1H), 3.67 (s, 2H), 3.55 – 3.49 (m, 1H), 3.45 (d, *J* = 11.6 Hz, 2H), 3.06 (d, *J* = 8.9 Hz, 2H), 3.00 – 2.89 (m, 4H), 2.84 – 2.76 (m, 4H), 2.69 (dt, *J* = 16.6, 5.5 Hz, 1H), 2.39 (m, 2H), 2.25 (s, 3H), 2.13 (t, *J* = 7.4 Hz, 2H), 1.61 (m, 2H), 1.53 (m, 2H), 1.29 (m, 4H).

Synthesis of **JHK-02-117**:

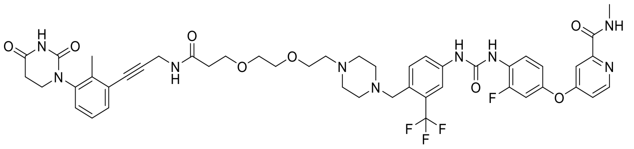

The corresponding compound was prepared following **Procedure C** using compound **3i** (3 mg, 1 eq) Purification by HPLC with MeOH/H_2O (0.035% TFA) to afford the product as a white solid (2.2 mg, 60%). LC/MS: m/z 946.61 [M+1]$^{+}$. ^{1}H NMR (500 MHz, DMSO) δ 10.38 (s, 1H), 9.52 (s, 1H), 8.78 (dd, *J* = 12.8, 3.8 Hz, 2H), 8.54 (d, *J* = 5.6 Hz, 1H), 8.43 (t, *J* = 5.4 Hz, 1H), 8.17 (t, *J* = 9.0 Hz, 1H), 8.00 (d, *J* = 2.1 Hz, 1H), 7.70 – 7.59 (m, 2H), 7.42 (d, *J* = 2.6 Hz, 1H), 7.37 – 7.29 (m, 3H), 7.24 (t, *J* = 7.7 Hz, 1H), 7.20 (dd, *J* = 5.6, 2.6 Hz, 1H), 7.07 (ddd, *J* = 9.1, 2.7, 1.3 Hz, 1H), 4.15 (d, *J* = 5.4 Hz, 2H), 3.78 (dd, *J* = 5.2, 2.5 Hz, 1H), 3.73 (t, *J* = 5.0 Hz, 2H), 3.67 (s, 2H), 3.64 (t, *J* = 6.3 Hz, 2H), 3.59 – 3.51 (m, 6H), 3.51 – 3.39 (m, 4H), 3.29 (t, *J* = 5.0 Hz, 2H), 3.07 (s, 2H), 2.91 (d, *J* = 10.3 Hz, 2H), 2.83 – 2.75 (m, 4H), 2.69 (dt, *J* = 11.2, 5.5 Hz, 1H), 2.47 (s, 1H), 2.38 (t, *J* = 6.0 Hz, 2H), 2.25 (s, 3H).

Synthesis of **JHK-02-065-2**:

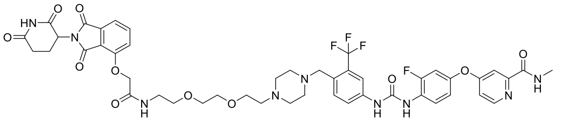

The corresponding compound was prepared following **Procedure B** using 2-((2-(2,6-dioxopiperidin-3-yl)-1,3-dioxoisoindolin-4-yl)oxy)acetic acid (8.6 mg, 24 µmol, 1.1 eq.) and intermediate **3d** (16 mg, 1 eq). Purification by HPLC with MeOH/H_2O (0.035% TFA) to afford the product as a white solid (4.8 mg, 20%). LC/MS: m/z 992.64 [M+1]$^{+}$. ^{1}H NMR (500 MHz, DMSO) δ 11.13 (s, 1H), 9.50 (s, 1H), 8.78 (dd, *J* = 17.2, 3.6 Hz, 2H), 8.54 (dd, *J* = 5.6, 1.7 Hz, 1H), 8.17 (t, *J* = 9.1 Hz, 1H), 7.98 (dd, *J* = 8.6, 2.0 Hz, 2H), 7.82 (dd, *J* = 8.5, 7.3 Hz, 1H), 7.63 – 7.61 (m, 1H), 7.52 – 7.49 (m, 1H), 7.43 – 7.39 (m, 2H), 7.35 (dd, *J* = 11.6, 2.7 Hz, 1H), 7.20 (dd, *J* = 5.6, 2.7 Hz, 1H), 7.12 – 7.04 (m, 1H), 5.12 (dd, *J* = 12.8, 5.5 Hz, 1H), 4.79 (d, *J* = 3.8 Hz, 2H), 3.74 (q, *J* = 4.1 Hz, 2H), 3.68 – 3.62 (m, 2H), 3.62 – 3.51 (m, 5H), 3.48 (t, *J* = 5.8 Hz, 4H), 3.33 (q, *J* = 6.5 Hz, 4H), 3.16 – 3.00 (m, 2H), 2.90 (ddd, *J* = 17.0, 14.0, 5.3 Hz, 3H), 2.80 (d, *J* = 4.8 Hz, 3H), 2.65 – 2.52 (m, 2H), 2.48 (s, 2H), 2.08 – 2.01 (m, 1H).

Negative compound synthesis:

Synthesis of **2-((1-(2,6-dioxopiperidin-3-yl)-3-methyl-2-oxo-2,3-dihydro-1H-benzo[d]imidazol-5-yl)oxy)-N-(8-hydroxyoctyl)acetamide (compound 5)**:

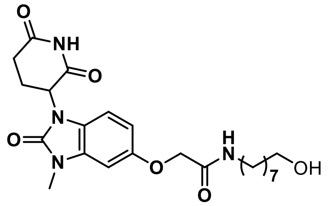

To a solution of 2-((1-(2,6-dioxopiperidin-3-yl)-3-methyl-2-oxo-2,3-dihydro-1H-benzo[d]imidazol-5-yl)oxy)acetic acid (150 mg, 1 eq), HATU (342 mg, 2 eq) in DMF (3 mL) was added DIPEA (240 μL, 3 eq), and the reaction was stirred at room temperature for 15 min. 8-aminooctan-1-ol (65.4 mg, 1 eq) was added and the mixture was stirred at room temperature for overnight. The crude was then purified by reverse phase combiflash with ACN/H_2O to give compound **5** as a white solid (70 mg, 33%). LC/MS: m/z 461.37 $[M+1]^+$.

Synthesis of N-(8-hydroxyoctyl)-2-((3-methyl-1-(1-methyl-2,6-dioxopiperidin-3-yl)-2-oxo-2,3-dihydro-1H-benzo[d]imidazol-5-yl)oxy)acetamide (compound 6)

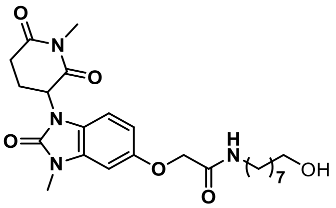

To a solution compound 5 (68 mg, 1 eq) in acetonitrile (1 mL) was added iodomethane (31 mg, 14 μL, 1.5 eq) and potassium carbonate (41 mg, 2 eq) and the mixture was stirred at 50 °C for 12 h. Purification by silica gel chromatography using DCM to 10% MeOH with DCM as a gradient eluent to yield compound 6 (44 mg, 66%). LC/MS: m/z 475.42 $[M+1]^+$.

Synthesis of **JHK-02-137 (JHK-02-108-2 neg)**:

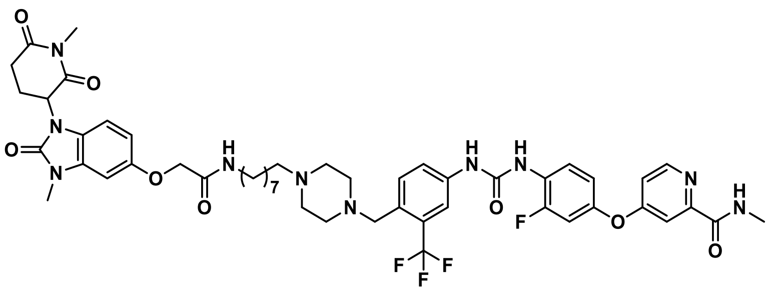

To a solution of compound 6 (16 mg, 1eq) in DCM (1 mL) was added Dess–Martin periodinane (15 mg, 1.1 eq) and the mixture was stirred at RT for 2 h. After checking LC/MS of formation of aldehyde, solution was celite filter with DCM, directly add DIPEA (23 μL, 4 eq) and compound **2b** (21 mg, 1 eq) with sodium triacetoxyhydroborate (28 mg, 4 eq) and the mixture was stirred at RT for 24 h. Purification

with by HPLC with MeOH/H_2O (0.035% TFA) to afford the product as a white solid (5 mg, 16%). LC/MS: m/z 1003.45 $[M+1]^+$. ^1H NMR (500 MHz, DMSO) δ 9.63 (s, 1H), 8.86 (s, 1H), 8.79 (d, J = 5.0 Hz, 1H), 8.53 (d, J = 5.6 Hz, 1H), 8.17 (t, J = 9.0 Hz, 1H), 8.05 (t, J = 5.8 Hz, 1H), 8.00 (d, J = 2.2 Hz, 1H), 7.67 – 7.57 (m, 2H), 7.43 (d, J = 2.6 Hz, 2H), 7.33 (dd, J = 11.6, 2.7 Hz, 1H), 7.19 (dd, J = 5.6, 2.6 Hz, 1H), 7.10 – 7.00 (m, 1H), 6.92 (d, J = 2.4 Hz, 1H), 6.66 (dd, J = 8.6, 2.4 Hz, 1H), 5.39 (dd, J = 13.1, 5.3 Hz, 1H), 5.33 (t, J = 4.9 Hz, 1H), 4.46 (s, 2H), 3.58 (s, 2H), 3.32 (s, 3H), 3.14 – 3.09 (m, 2H), 3.03 (s, 3H), 2.80 (d, J = 4.9 Hz, 4H), 2.77 – 2.63 (m, 3H), 2.00 (dt, J = 17.4, 7.0 Hz, 5H), 1.45 (dt, J = 13.6, 6.9 Hz, 6H), 1.24 (d, J = 3.0 Hz, 10H).

Synthesis of 2-(4-(2,6-dioxopiperidin-3-yl)phenoxy)-N-(2-(2-(2-hydroxyethoxy)ethoxy)ethyl)-acetamide (compound 7)

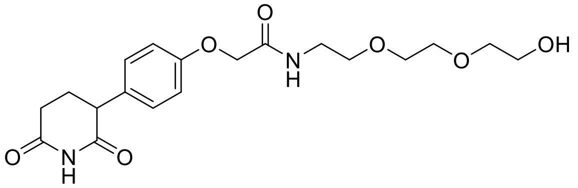

To a solution of 2-(4-(2,6-dioxopiperidin-3-yl)phenoxy)acetic acid (80 mg, 1 eq), HATU (230 mg, 2 eq) in DMF (2 mL) was added DIPEA (160 µL, 3 eq), and the reaction was stirred at room temperature for 15 min. 2-(2-(2-aminoethoxy)ethoxy)ethan-1-ol (45 mg, 1 eq) was added and the mixture was stirred at room temperature for overnight. The crude was then purified by reverse phase combiflash with ACN/H_2O to give compound **7** as a white solid (40 mg, 33%). LC/MS: m/z 395.14 $[M+1]^+$.

Synthesis of N-(2-(2-(2-hydroxyethoxy)ethoxy)ethyl)-2-(4-(1-methyl-2,6-dioxopiperidin-3-yl)phenoxy)acetamide (compound 8)

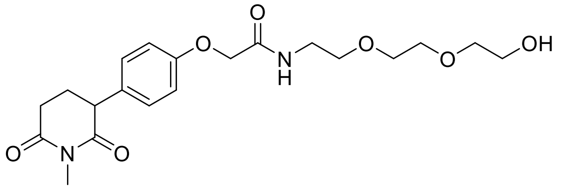

To a solution compound 5 (126 mg, 1 eq) in acetonitrile (1 mL) was added iodomethane (50 mg, 1.1 eq) and potassium carbonate (88.3 mg, 2 eq) and the mixture was stirred at 60 °C for 12 h. Purification by silica gel chromatography using DCM to 10% MeOH with DCM as a gradient eluent to yield compound 6 (100 mg, 76%). The crude was then purified by reverse phase combiflash with ACN/H_2O to give compound **8** as a white solid. LC/MS: m/z 409.15 $[M+1]^+$.

Synthesis of JHK-02-138 (JHK-02-102-1 neg)

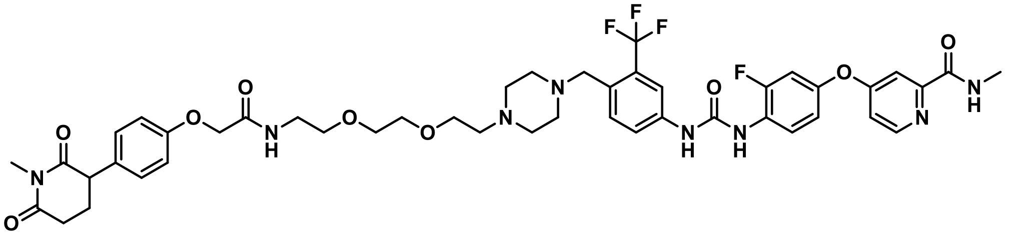

To a solution of compound 8 (12 mg, 1eq) in DCM (1 mL) was added Dess–Martin periodinane (11 mg, 1.1 eq) and the mixture was stirred at RT for 2 h. After checking LC/MS of formation of aldehyde, solution was celite filter with DCM, directly add DIPEA (13 µL, 4 eq) and compound **2b** (12 mg, 1 eq) with sodium triacetoxyhydroborate (11 mg, 2 eq) and the mixture was stirred at RT for 24 h. Purification with by HPLC with MeOH/H_2O (0.035% TFA) to afford the product as a white solid (7 mg, 30%). LC/MS: m/z 937.33 $[M+1]^+$. ^{1}H NMR (500 MHz, DMSO) δ 9.53 (s, 1H), 8.87 – 8.77 (m, 2H), 8.54 (d, *J* = 5.5 Hz, 1H), 8.16 (td, *J* = 9.0, 7.1 Hz, 1H), 8.10 (t, *J* = 5.9 Hz, 1H), 8.00 (d, *J* = 1.9 Hz, 1H), 7.66 – 7.56 (m, 2H), 7.42 (d, *J* = 2.6 Hz, 1H), 7.35 (dd, *J* = 11.5, 2.7 Hz, 1H), 7.20 (dt, *J* = 5.6, 2.6 Hz, 1H), 7.18 – 7.13 (m, 2H), 7.10 – 7.05 (m, 1H), 6.93 – 6.90 (m, 2H), 4.47 (s, 2H), 3.89 (dd, *J* = 11.6, 4.9 Hz, 2H), 3.77 – 3.69 (m, 6H), 3.60 – 3.55 (m, 8H), 3.46 (t, *J* = 6.1 Hz, 4H), 3.38 – 3.27 (m, 4H), 3.14 (td, *J* = 7.3, 4.2 Hz, 1H), 3.07 (m, 2H), 3.02 (s, 3H), 2.90 (m, 2H), 2.80 (d, *J* = 4.8 Hz, 3H), 2.68 – 2.60 (m, 1H), 2.44 (d, *J* = 15.4 Hz, 1H), 2.16 (qd, *J* = 11.5, 3.7 Hz, 1H), 1.99 (dt, *J* = 13.1, 4.7 Hz, 1H).

2. Supplementary Figures

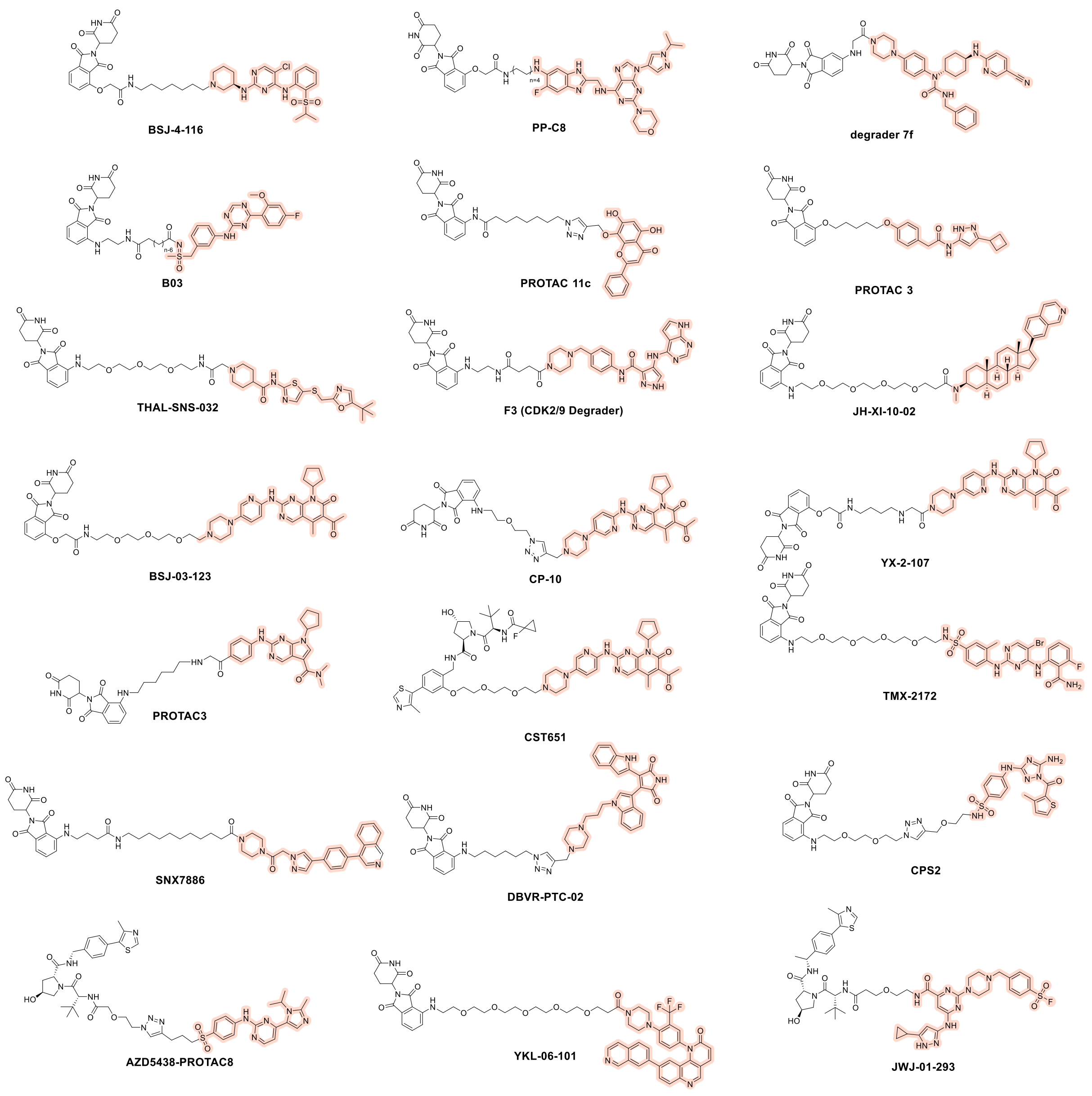

Figure S1. Chemical structure of bivalent degraders targeting CDK families.

Table S1. List of bivalent compounds degrading CDK families and the original targets of their corresponding warheads.

Compound	Degradation target	Parent inhibitor and structure	Target of parent inhibitor	Reference

BSJ-4-116	CDK12	THZ531	CDK12/13	10.1038/s41589-021-00765-y.
PP-C8	CDK12	SR-4835	CDK12/13	10.1016/j.ejmech.2021.114012
compound 7f	dual CDK12/13 degraders	inhibitor 3	CDK12/13	compound
B03	CDK9	**Atuveciclib** (BAY-1143572)	PTEFb/CDK9	10.1016/j.ejmech.2020.113091
PROTAC 11c	CDK9	Wogonin	CDK9	10.1016/j.bioorg.2018.08.028
PROTAC 3 (30)	CDK9	aminopyrazole	CDK5 CDK9	10.1039/c7cc03879h
THAL-SNS-032	CDK9	SNS-032	ATP competitive CDK9 inhibitor	10.1038/nchembio.2538
F3 (34)	CDK2/9	FN-1501	CDK2/4/6/FLT3	10.1016/j.ejmech.2019.111952
JH-XI-10-02	CDK8	JH-VIII-49	CDK19, CDK8, NEK1	10.1021/acsmedchemlett.8b00011
BSJ-03-123	CDK6	Palbociclib	CDK4/6	10.1016/j.chembiol.2018.11.006
CP-10	CDK6	Palbociclib	CDK4/6	10.1021/acs.jmedchem.9b00871
YX-2-107	CDK6	Palbociclib	CDK4/6	10.1182/blood.2019003604
PROTAC 3 (37)	CDK2/4/6	compound 2 modified from ribociclib	CDK2/4/6	10.1016/j.ejmech.2020.112903
CST651	CDK6	palbociclib	CDK4/6	10.1039/d0sc00167h
TMX-2172	CDK2/5	TMX2039	CDK1/2/5	10.1002/anie.202004087.
SNX7886	CDK8/19	BI1347	CDK8/19	10.1093/nar/gkad538
DBVR-PTC-02	GSK-3β, CDK5	N/A	-	https://doi.org/10.1016/j.bmc.2025.118085
CPS2	CDK2, AURKA	JNJ-7706621	CDK1, CDK2, AURKA, AURKB	10.1038/s41589-021-00742-5.

AZD5438-PROTAC8	CDK2	AZD5438	CDK1, CDK2, CDK9	10.1016/j.ejmech.2021.113849
YKL-06-101	CDK8	Torin1	CDK8, mTOR	10.1038/s41467-019-12656-x.
JWJ-01-293	AURKA, ITK, PRKAA1, NEK9, CDK6	Pan-kinase binder	Multi-kinase	10.1002/anie.202417272

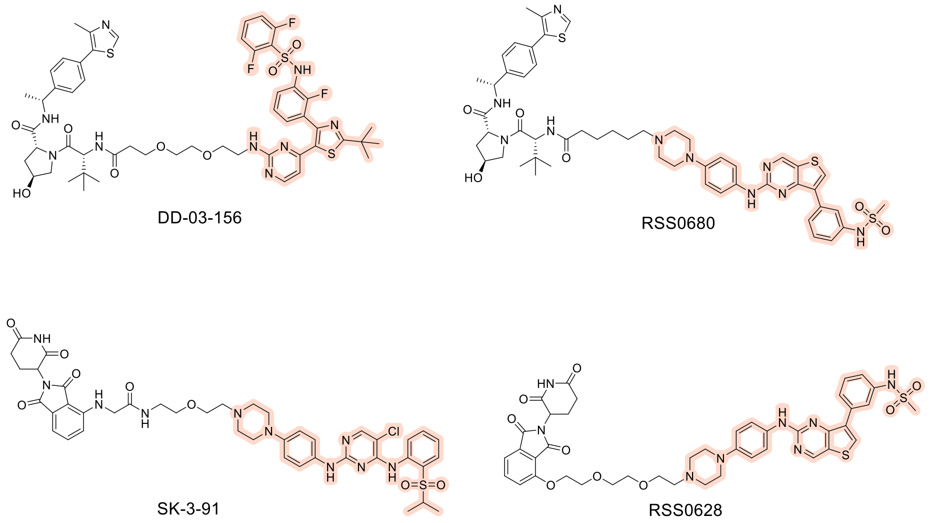

Figure S2. Chemical structure of bivalent degraders from multi-kinase inhibitor warhead and degrading CDK families.

Table S2. List of bivalent compounds from multi-kinase inhibitor warheads and degrading CDK families and the original targets of their corresponding warheads.

Compound	Degradation target	Parent inhibitor and structure	Target of parent inhibitor	Reference
DD-03-156	CDK17, LIMK2	dabrafenib	BRAF	10.1016/j.cell.2020.10.038
SK-3-91	PTK2B, ITK, FER, AURKA (strong, (log2FC < -2), CDK2, CDK5, CDK6, CDK9, CDK12, CDK13, CDK18 (weak, log2FC > -1.5)	des methoxy-TAE684	Multi-kinases	10.1016/j.cell.2020.10.038
RSS0680	Wee1, CDK6, NEK9, AAK1, CDK4, STK17B	FLT3-IN-17 (VHL)	FLT3, FAK	10.1016/j.cell.2020.10.038

RSS0628	NEK9, CDK6, Wee1, PTK2B, AAK1, ZFP91, CDK4	FLT3-IN-17 (CRBN)	FLT3, FAK	10.1016/j.cell.2020.10.038

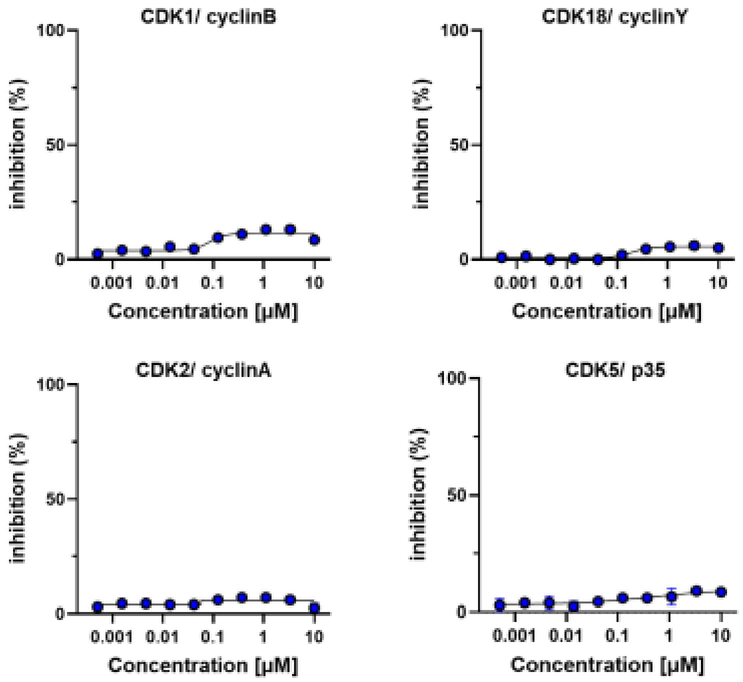

Figure S3. Dose-dependent inhibition of CDK1/cyclinB, CDK18/cyclinY, CDK2/ cyclinA, and CDK5/p35 activity by **STT-03-123**, measured by the Z’-lyte^{TM} kinase assay.

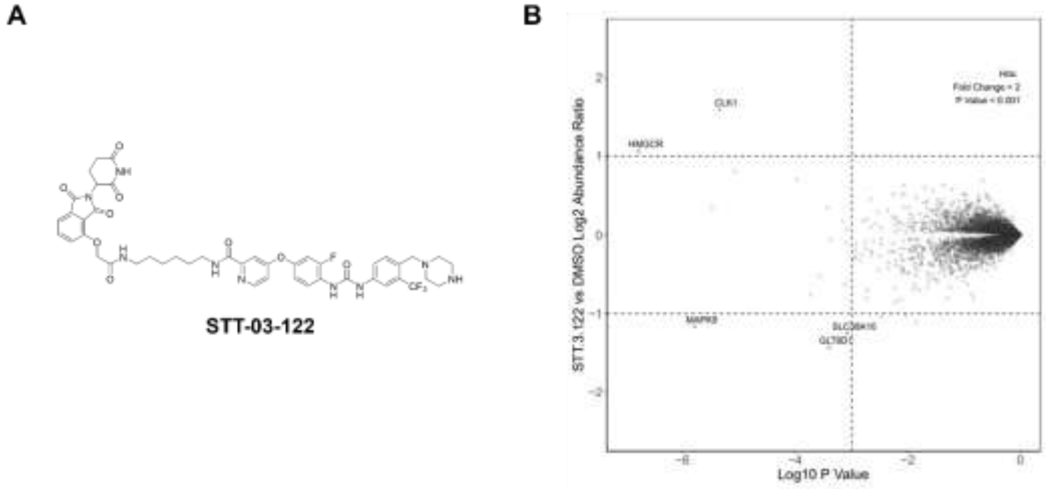

Figure S4. Hinge-binding region modified ‘front’ degrader, **STT-03-122**. (A) Chemical structure and (B) quantitative proteome-wide mass spectrometry in MOLT-4 cells after 5 hours treatment with 1 μM **STT-03-122**.

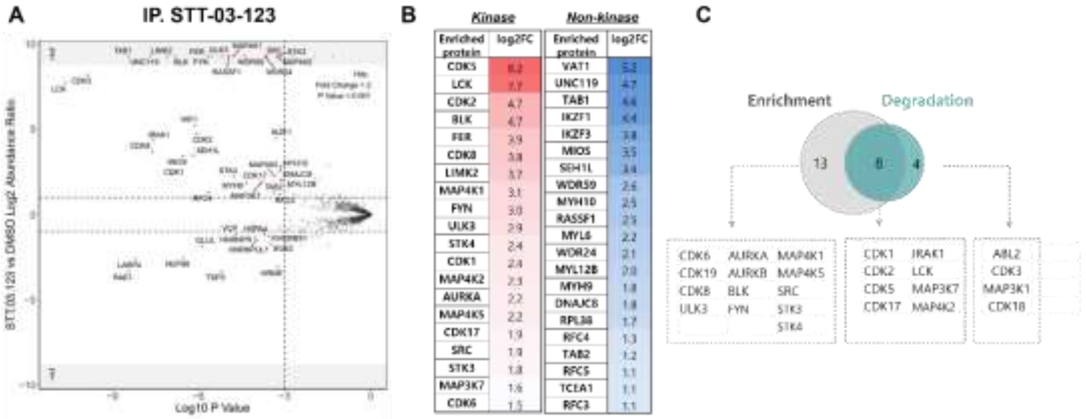

Figure S5. **STT-03-123** targets identified by CRBN IP-MS and whole-proteome degradation. (A) Scatterplots depicting relative protein abundance following Flag-CRBN-DDB1ΔB enrichment from MOLT-4 in-lysate treatment with 1 μM **STT-03-123** and recombinant Flag-CRBN-DDB1ΔB spike in. Cutoffs: FC>1.5; P-value<0.001. (B) Table displays the $\log_2$ fold change ($\log_2$FC) values of the top enriched proteins upon **STT-03-123** treatment in CRBN IP-MS. (C) The Venn diagram compares proteins enriched by **STT-03-123** in IP-MS with those significantly degraded in whole-proteome analysis, highlighting overlapping targets that are both recruited to CRBN and degraded upon treatment.

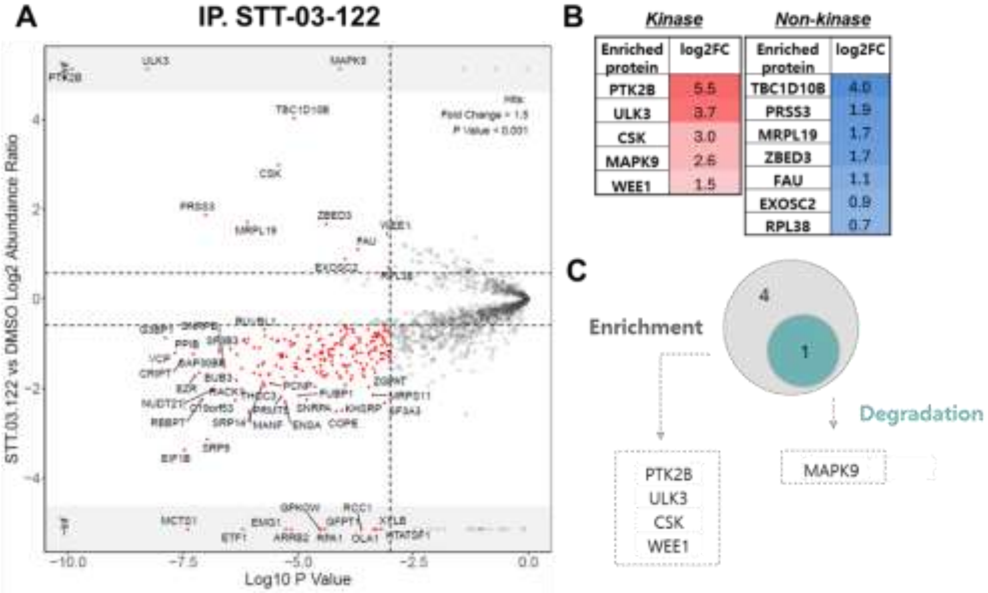

Figure S6. **STT-03-122** targets identified by CRBN IP-MS and whole-proteome degradation. (A) Scatterplots depicting relative protein abundance following Flag-CRBN-DDB1ΔB enrichment from MOLT-4 in-lysate treatment with 1 μM **STT-03-122** and recombinant Flag-CRBN-DDB1ΔB spike in. Cutoffs: FC>1.5; P-value<0.001. (B) Table displays the $\log_2$ fold change ($\log_2$FC) values of the top enriched proteins upon **STT-03-122** treatment in CRBN IP-MS. (C) The Venn diagram compares proteins enriched by **STT-03-12**2 in IP-MS with those significantly degraded in whole-proteome analysis, highlighting overlapping targets that are both recruited to CRBN and degraded upon treatment.

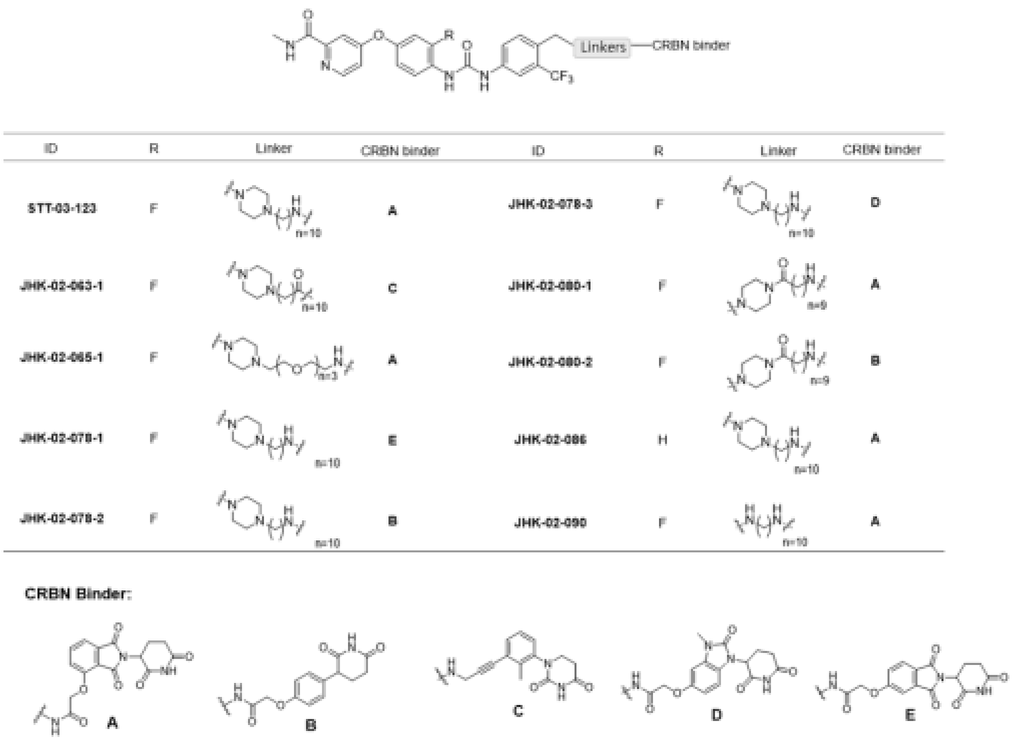

Figure S7. Chemical structures of heterobivalent degraders are shown in Figure 3A.

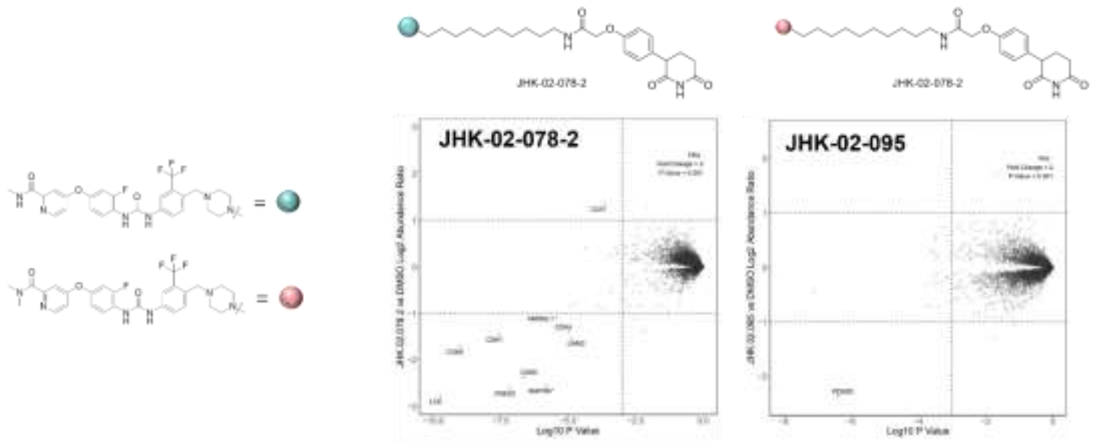

Figure S8. Volcano plots comparing global proteomics profiles of 1 µM of **JHK-02-078-2** and **JHK-02-095** in MOLT-4 cells, 6 h treatment. Dimethylation of a hinge binding site in **JHK-02-095** abolished kinase degradation activity.

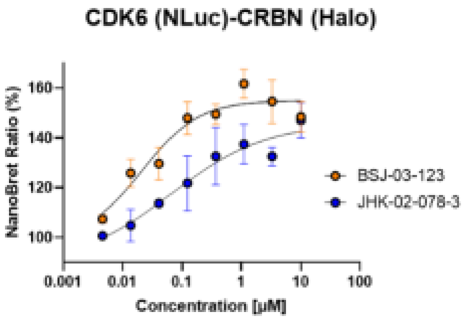

Figure S9. NanoBRET assay for evaluating ternary complex formation between CRBN (HaloTag-CRBN) and CDK6 (Nanoluciferase-CDK6) fusion proteins. Comparison of **JHK-02-078-3** and type-I CDK6 degrader **BSJ-03-123**. **JHK-02-078-3** data corresponds to Figure 3C.

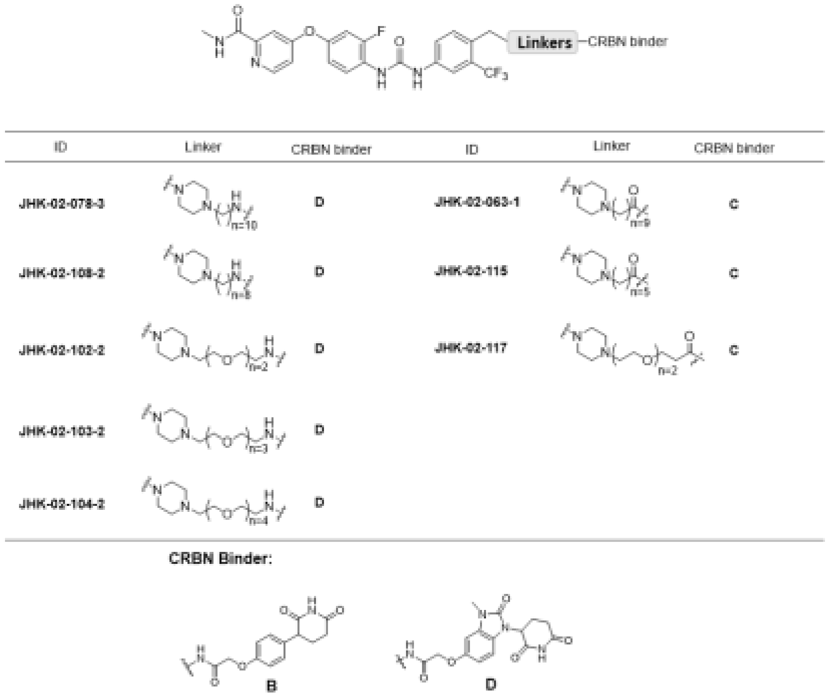

Figure S10. Chemical structure of linker modified regorafenib-based heterobivalent degraders correspond to Figure 3E and F.

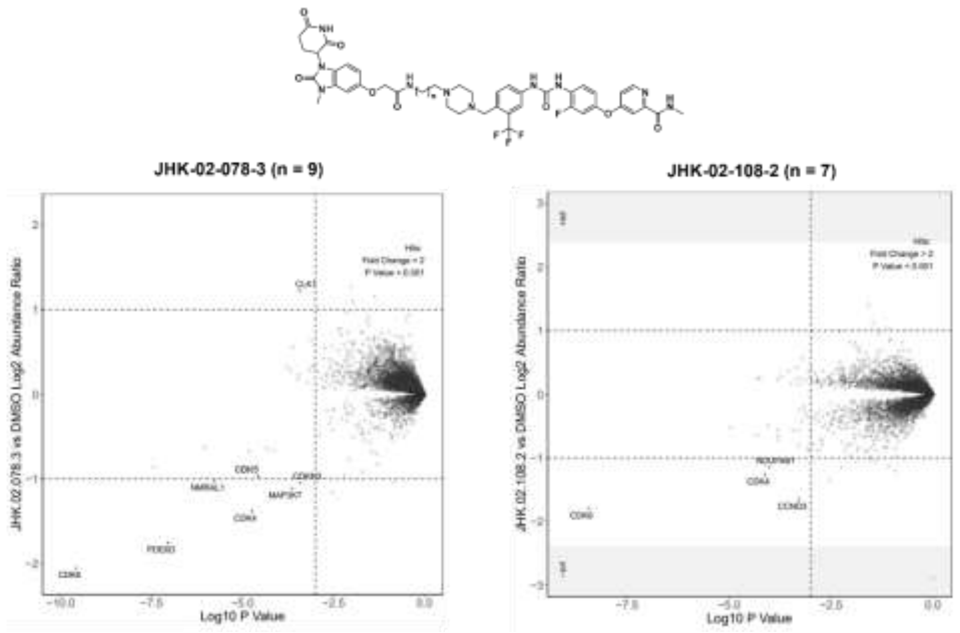

Figure S11. Scatterplot comparison of relative fold changes in protein abundance following 5 h treatment of MOLT-4 cells with 1 μM **JHK-02-078-3** or **JHK-02-108-2** (corresponding to Figure 3G).

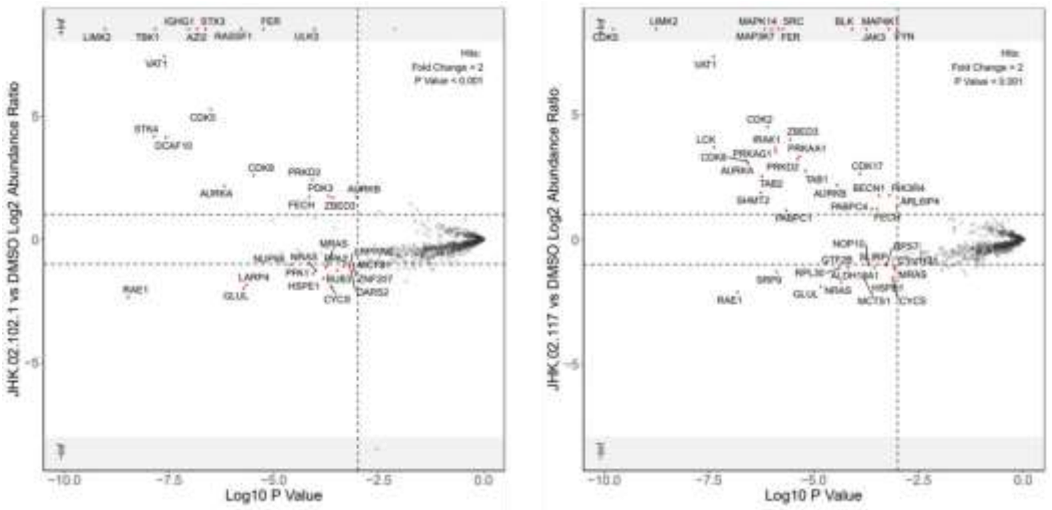

Figure S12. Scatterplot depicting relative protein abundance following Flag-CRBN-DDB1DB enrichment from in-lysate treatment with **JHK-02-102-1** (left) and **JHK-02-117** (right) and recombinant Flag-CRBN-DDB1DB spike in. Scatterplot displays fold change in abundance to DMSO.

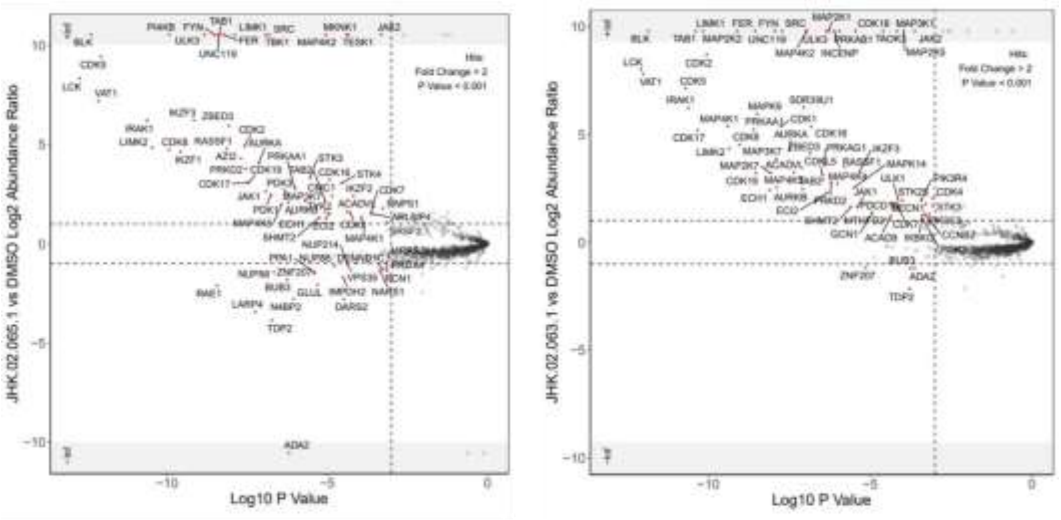

Figure S13. Scatterplot depicting relative protein abundance following Flag-CRBN-DDB1DB enrichment from in-lysate treatment with **JHK-02-065-1** (left) and **JHK-02-063-1** (right) and recombinant Flag-CRBN-DDB1DB spike in. Scatterplot displays fold change in abundance to DMSO.

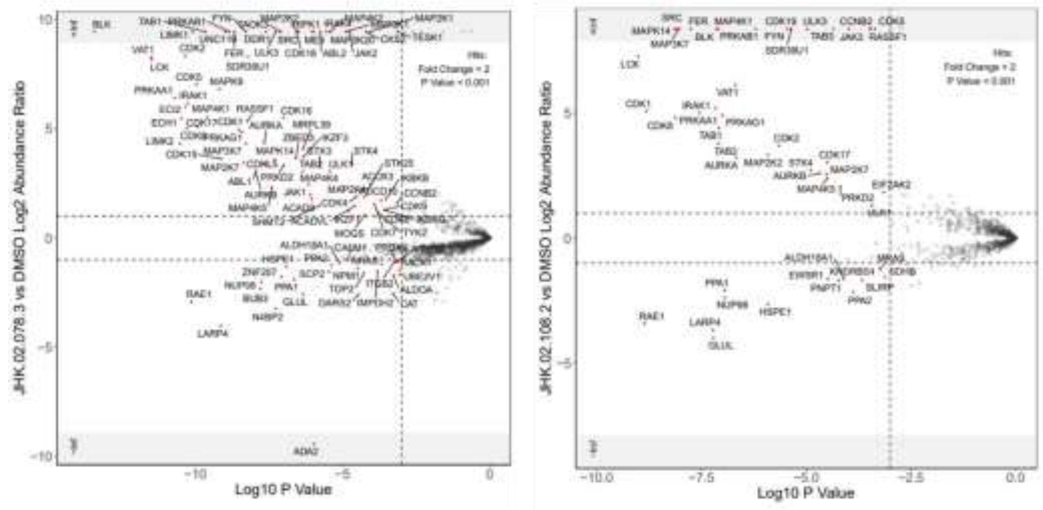

Figure S14. Scatterplot depicting relative protein abundance following Flag-CRBN-DDB1DB enrichment from in-lysate treatment with **JHK-02-078-3** (left) and **JHK-02-108-2** (right) and recombinant Flag-CRBN-DDB1DB spike in. Scatterplot displays fold change in abundance to DMSO.

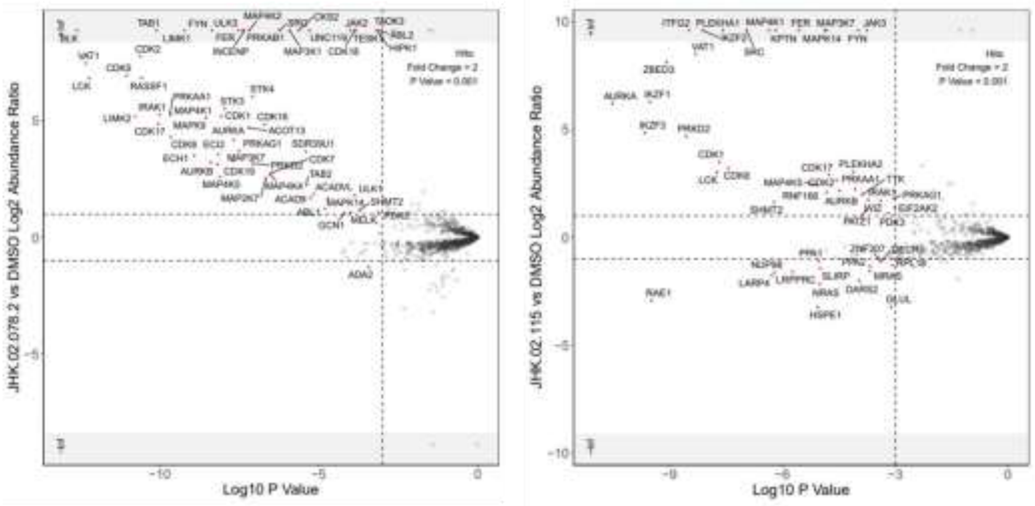

Figure S15. Scatterplot depicting relative protein abundance following Flag-CRBN-DDB1DB enrichment from in-lysate treatment with **JHK-02-078-2** (left) and **JHK-02-115** (right) and recombinant Flag-CRBN-DDB1DB spike in. Scatterplot displays fold change in abundance to DMSO.

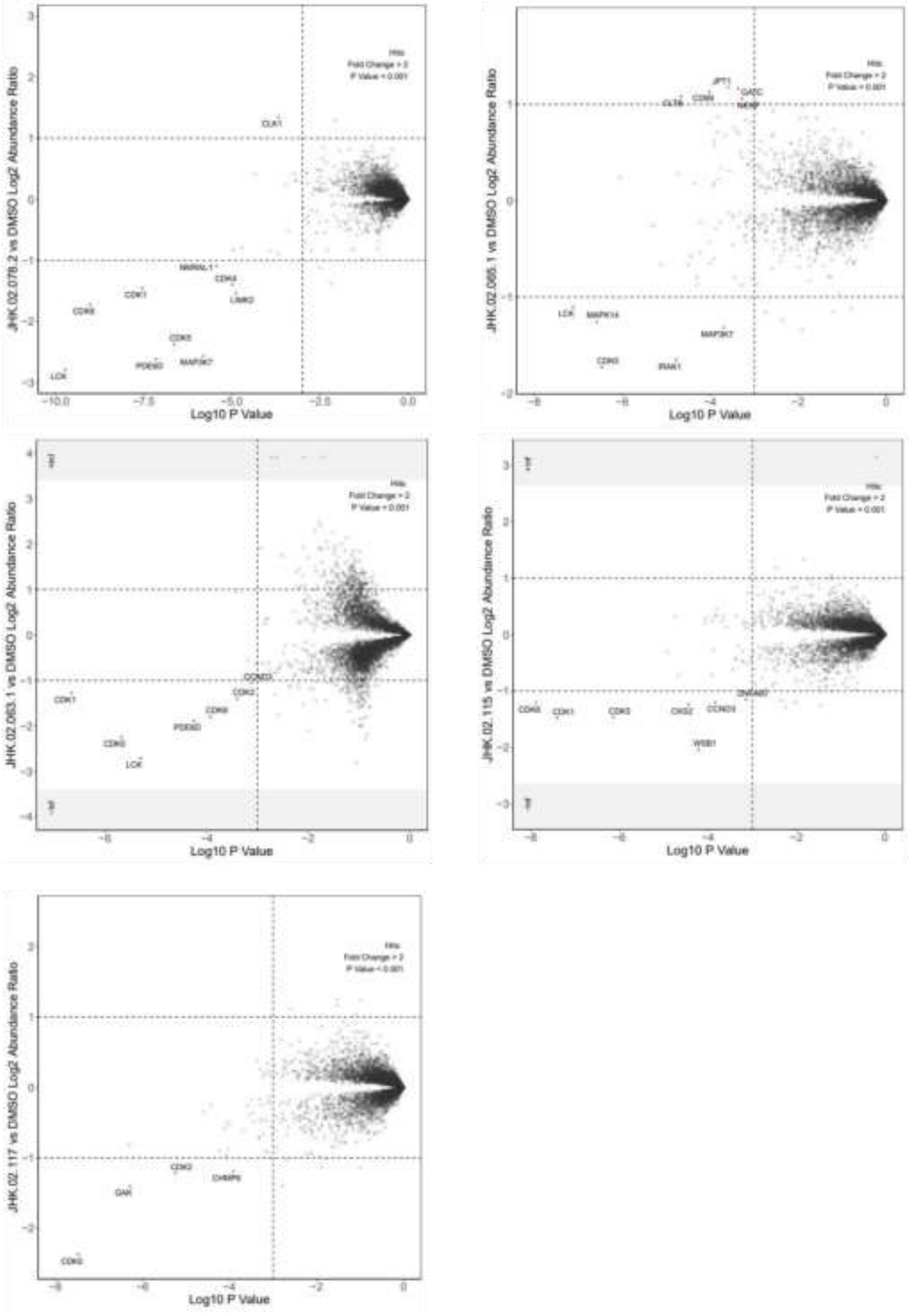

Figure S16. Scatterplot depicting relative FC in protein abundance following treatment of MOLT-4 cells with 1 μM of **JHK-02-078-2** (correspond to Figure S6), **JHK-02-078-3** (correspond to Figure S9), **JHK-02-065-1**, **JHK-02-063-1**, **JHK-02-115**, and **JHK-02-117** for 5 h.

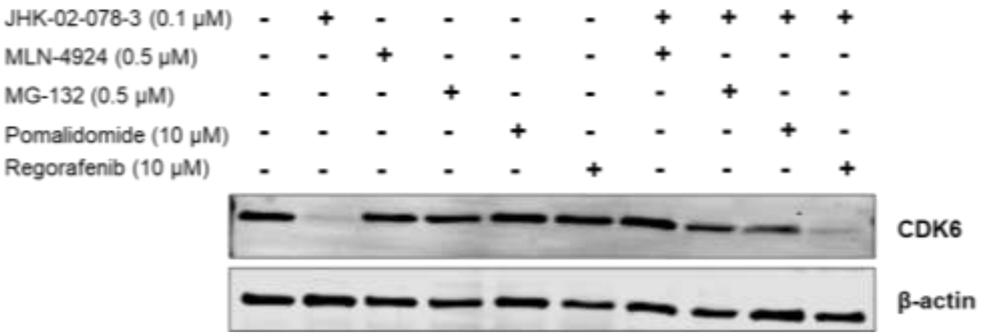

Figure S17. Competition experiment with type-II CDK6 degrader **JHK-02-078-3** in MOLT-4 cells with 5 h treatment. MLN-4924, MG-132, Pomalidomide, and regorafenib were pretreated for 2 h before **JHK-02-078-3** treatment.

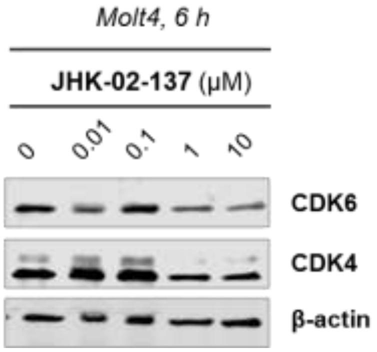

Figure S18. Western blots of CRBN-bump compound, **JHK-02-137,** show no degradation of CDK6 and CDK4 in MOLT-4 cells after 6 h treatments.

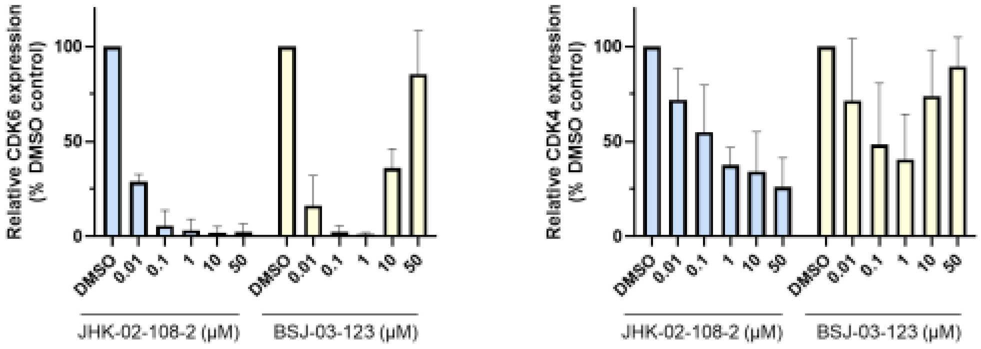

Figure S19. Western blot quantification CDK6 and CDK4 of **JHK-02-108-2** and **BSJ-03-123** in MOLT-4 cells after 6 h treatments. Data corresponds to Figure 5B. Protein expression was normalized using β-Actin as a loading control.

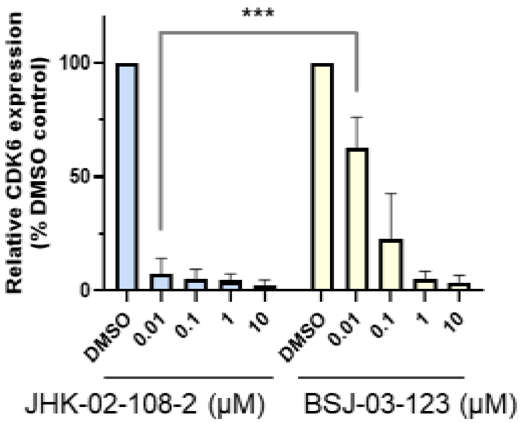

Figure S20. Western blot quantification CDK6 of **JHK-02-108-2** and **BSJ-03-123** in MOLT-4 cells after 24 h treatments. Data corresponds to Figure 5C. Protein expression was normalized using β-Actin as a loading control.

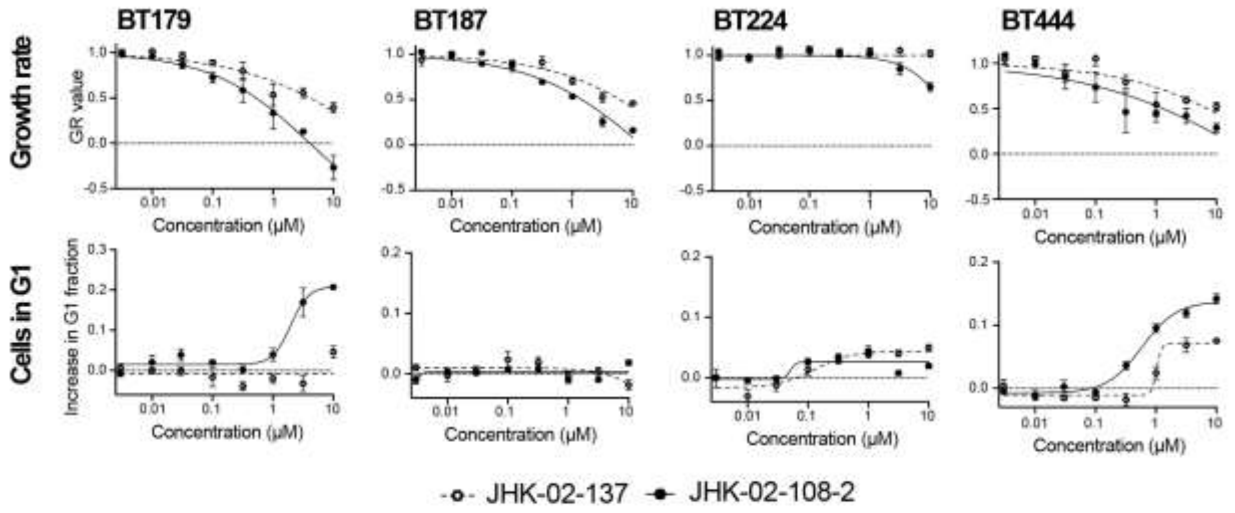

Figure S21. Growth rate inhibition values and the fraction of cells in G1 for GBM cell lines treated with the indicated compounds for 72 h. Error bars represent the standard error of the mean (n=3).

Table S3. Growth inhibition GR_{50} values of **JHK-02-108-2** and **JHK-02-137** in GBM cell lines, corresponding to Figure 6A and Figure S21. GR_{50} is from the fit to the mean of three independent experiments.

GR_{50} (µM)	JHK-02-108-2	JHK-02-137
BT145	0.64	> 10
BT179	0.522	2.40
BT187	1.01	4.89
BT224	ND	ND
BT444	0.322	ND

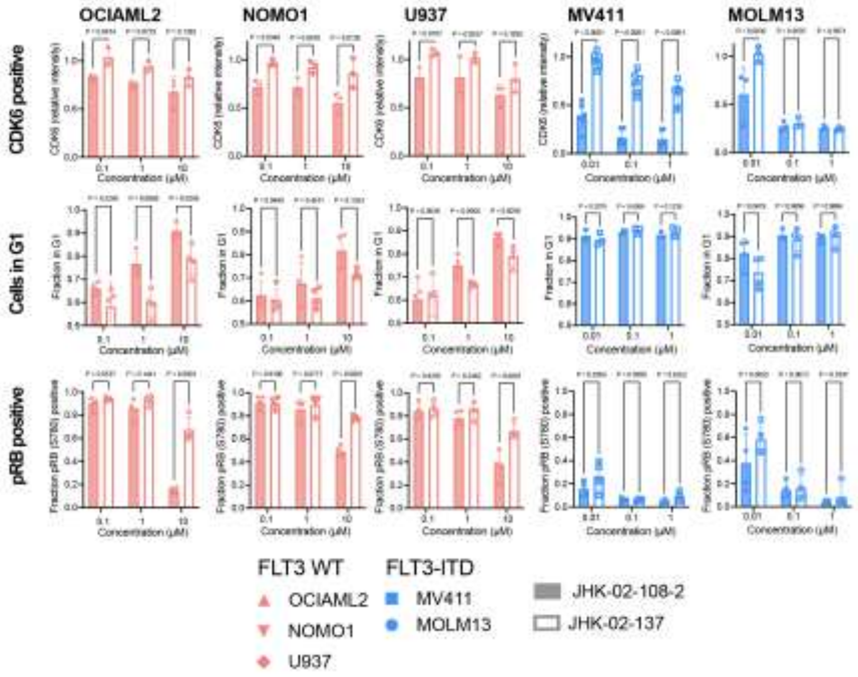

Figure S22. Median relative CDK6 intensity, the fraction of cells in G1, and the fraction of pRB (S780) positive cells in FTL3-WT and FTL3-ITD AML cell lines after 24 h treatments. Error bars represent standard deviation of the mean (n≥ 3).

Table S4. Relative viability IC_{50} values for **JHK-02-108-2** and **JHK-02-137** in AML cell lines, corresponding to Figure 5E. IC_{50} values represent the mean of three independent experiments.

IC_{50} (μM)	JHK-02-108-2	JHK-02-137
MV411	0.0029	0.0047
MOLM13	0.0024	0.0029
OCI-AML-2	3.39	8.58
NOMO1	3.31	12.80
U937	2.62	9.39

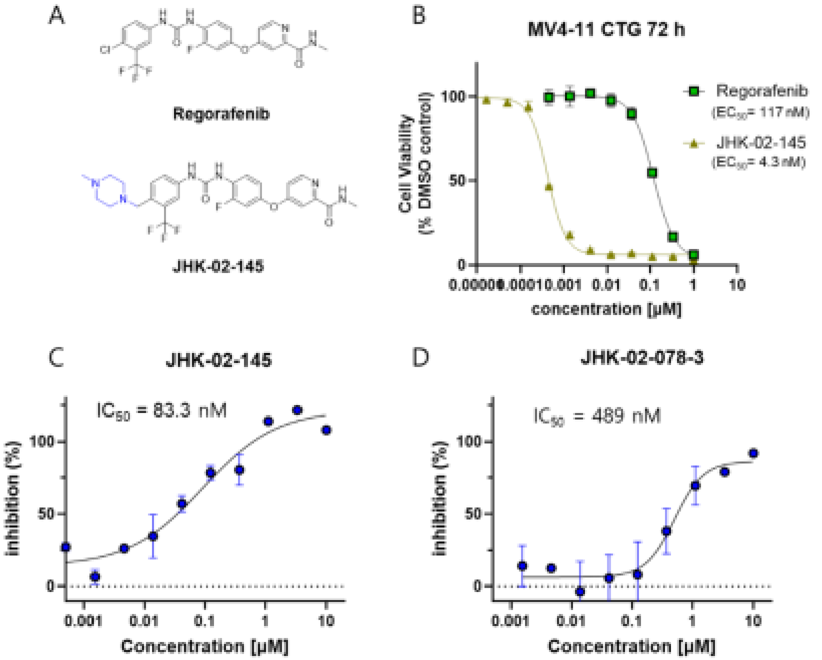

Figure S23. Enhanced sensitivity of the regorafenib-derived analog JHK-02-145 in FLT3-ITD–mutant AML models. (A) Chemical structure of regorafenib and **JHK-02-145**. (B) Cell viability of MV4-11 cell treated with the indicated compounds for 72 h. Dose-dependent inhibition of FLT3-ITD activity by (C) **JHK-02-145** and (D) **JHK-02-078-3**, measured using the LanthaScreen kinase assay.

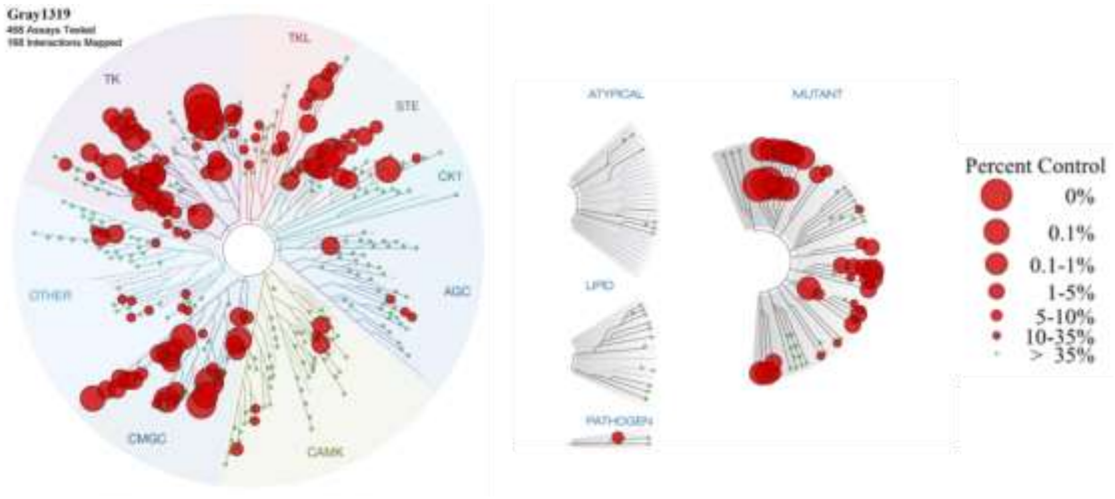

Figure S24. Kinase selectivity data of **JHK-02-108-2** (1 μM) based on *AMBIT KINOMEscan*. Percent control (%Ctrl) values represent the remaining kinase binding relative to the DMSO control.

Table S5. Kinase binary binding affinity and CRBN-mediated ternary complex enrichment for overlapping kinases identified in both the Ambit kinome scan and IP-MS pulldown of **JHK-02-108-2** (1 µM). Ambit %Ctrl reflects binary engagement strength (lower = stronger binding); Log2FC from IP-MS with CRBN reflects ternary complex enrichment. Kinases are categorized by Ambit binding: strong (≤10%, green), moderate (11–35%, yellow), weak (36–70%, orange), and non-binder (>70%, pink). [a] The Ambit %Ctrl/Log2FC ratio serves as a composite index: lower values indicate strong binary affinity with efficient ternary enrichment, whereas higher values suggest context-dependent cooperativity driven by CRBN-mediated complex formation.

Kinase	Ambit scan %Ctrl (Binary)	Ambit Category	Log2FC from IP-MS with CRBN (Ternary)	Ambit scan %Ctrl/IP-MS Log2FC
**MAP4K1**	0.1	Strong binder (≤10%)	4.3	0.0
**MAP3K7**	0.4	Strong binder (≤10%)	5.3	0.1
**BLK**	0.4	Strong binder (≤10%)	4.3	0.1
**CDK19**	0.7	Strong binder (≤10%)	2.7	0.2
**MAPK14**	0.8	Strong binder (≤10%)	5.1	0.1
**CDK17**	1.0	Strong binder (≤10%)	3.0	0.3
**MAP4K4**	1.2	Strong binder (≤10%)	1.2	1.0
**CDK8**	1.5	Strong binder (≤10%)	4.8	0.3
**LCK**	2.4	Strong binder (≤10%)	7.3	0.3
**CDK5**	2.6	Strong binder (≤10%)	1.9	1.4
**MAP2K7**	3.4	Strong binder (≤10%)	2.6	1.3
**ULK3**	3.7	Strong binder (≤10%)	2.4	1.5
**SRC**	5.0	Strong binder (≤10%)	3.8	1.3
**CDK2**	5.0	Strong binder (≤10%)	3.7	1.4
**JAK3**	9.0	Strong binder (≤10%)	2.3	4.0
**RAF1**	9.1	Strong binder (≤10%)	0.8	[illegible]
**FYN**	14.0	Moderate (11–35%)	3.5	4.0
**PRKAA1**	15.0	Moderate (11–35%)	5.0	3.0
**MAP4K5**	19.0	Moderate (11–35%)	2.4	7.9
**IRAK1**	21.0	Moderate (11–35%)	5.2	4.0
**CDK4**	23.0	Moderate (11–35%)	1.6	[illegible]
**ULK1**	28.0	Moderate (11–35%)	1.3	[illegible]
**PRKD2**	45.0	Weak (36–70%)	2.1	[illegible]
**AURKB**	45.0	Weak (36–70%)	2.6	[illegible]
**ITK**	48.0	Weak (36–70%)	1.0	[illegible]
**AURKA**	59.0	Weak (36–70%)	3.2	[illegible]
**FER**	59.0	Weak (36–70%)	5.1	[illegible]
**STK4**	65.0	Weak (36–70%)	2.7	[illegible]
**MAP2K2**	80.0	Non-binder (>70%)	3.3	[illegible]
**GSK3B**	82.0	Non-binder (>70%)	0.8	[illegible]
**EIF2AK2**	85.0	Non-binder (>70%)	1.9	[illegible]
**STK3**	100.0	Non-binder (>70%)	1.5	[illegible]

Table S6. In vitro metabolic stability of **JHK-02-108-2** in human and mouse liver microsomes (half-life determined in 0.5 mg/mL hepatic microsomes supplemented with NADPH).

	Species (T1/2 in minutes)		Species (Cl_{int} - µl/min/mg)	
Compound ID	Human	Mouse	Human	Mouse
**sunitinib**	38.4	23.6	36	59
**JHK-02-108-2**	82.0	44.8	17	31

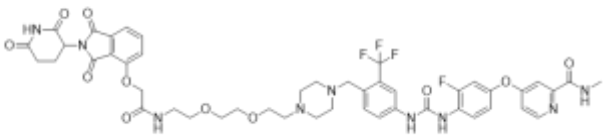

JHK-02-065-2

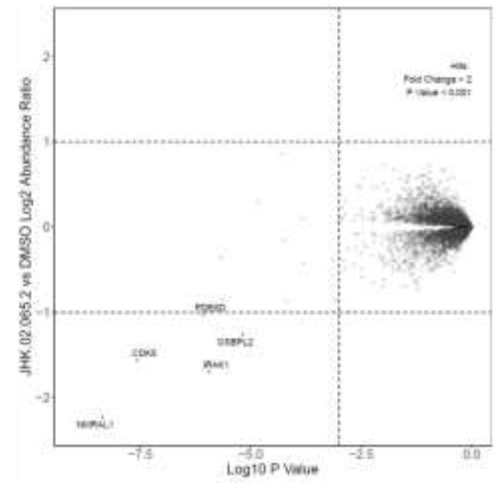

Figure S25. Chemical structure and a scatterplot depicting relative FC in protein abundance following treatment of MOLT-4 cells with 1 µM of **JHK-02-065-2** for 5 h.

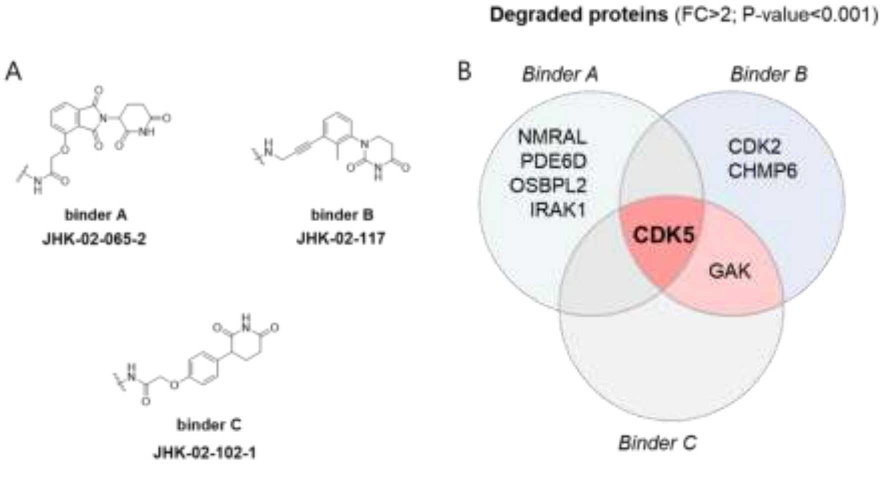

Figure S26. CRBN ligand–dependent modulation of degradation selectivity with a PEG2 linker with regorafenib back exit vector. (A) Chemical structures of the degraders. All PROTACs contain a PEG2 linker. (B) Venn diagram showing overlapping and distinct degradation targets identified across compounds, based on proteomics data presented in Figures 7B and S15.

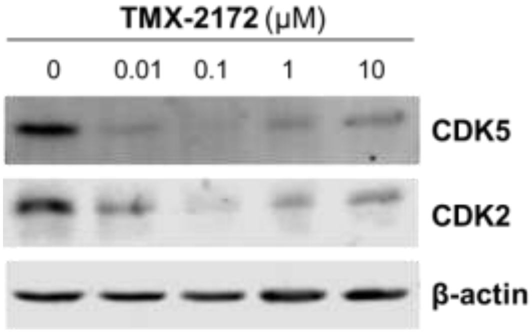

Figure S27. Western blot showing CDK2 and CDK5 degradation in MOLT-4 cells after 6 h treatment with **TMX-2172**.

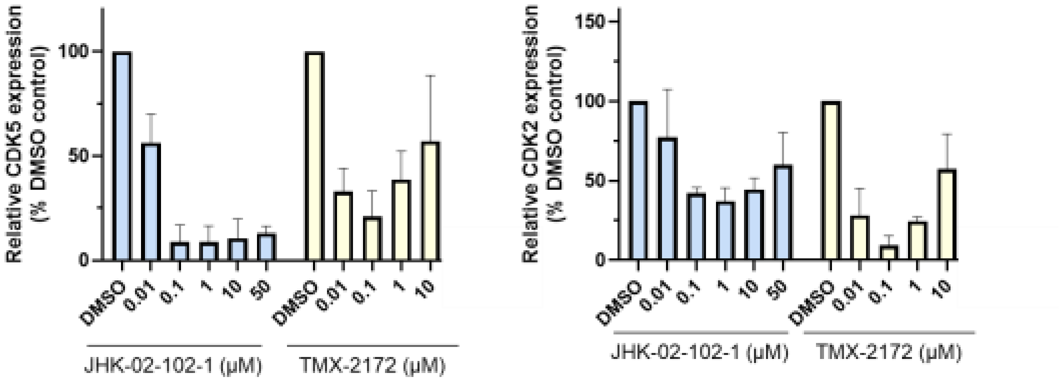

Figure S28. Western blot quantification CDK5, and CDK2 of **JHK-02-102-1** and **TMX-2172** in MOLT-4 cells after 6 h treatments. Data corresponds to Figure 7C and Figure S27. Protein expression was normalized using β-Actin as a loading control.

Methods

General Cell Biology Materials

Roswell Park Memorial Institute (RPMI) 1640 medium and Dulbecco's modified Eagle's medium (DMEM), Fetal bovine serum (FBS), penicillin–streptomycin (10,000 units/mL sodium penicillin G and 10,000 µg/mL streptomycin), trypsin–EDTA solution (1×), and phosphate-buffered saline (PBS; 1×) were purchased from Gibco (Grand Island, NY, USA). MG-132, MLN-4924 were purchased from MedChemExpress (Monmouth Junction, NJ, USA).

Mammalian Cell Culture

Human leukemia cell line, MOLT-4, was obtained from the American Type Culture Collection (ATCC, Manassas, VA, USA). Cells were incubated in a humidified atmosphere at 37 °C with 5% CO_2. Mycoplasma testing was conducted monthly using the MycoAlert Mycoplasma Detection Kit (Lonza, Basel, Switzerland), and all cell lines tested negative.

MV4-11, HEK293T and K562 cells were purchased from ATCC, OCI-AML-2, NOMO1, U937 and MOLM13 cells were obtained from the Laboratory of Systems Pharmacology; all lines were identity verified and maintained free of mycoplasma. HEK293T cells were cultured in DMEM medium, with 10% heat-inactivated FBS and 1% penicillin-streptomycin. K562, NOMO1, U937 and MOLM13 cells were cultured in RPMI medium, with 10% FBS and 1% penicillin-streptomycin. MV4-11 and OCI-AML-2 cells were cultured in IMDM supplemented with 10% FBS and 1% penicillin-streptomycin. NextGen glioblastoma cell lines[1] (BT145, BT179, BT187, BT224, and BT444) were obtained from the Ligon lab at Dana Farber Cancer Institute and maintained in neurospheres using the NeuroCult NS-A Proliferation Kit (StemCell Technologies) supplemented with 0.0002% heparin (StemCell Technologies), epidermal growth factor (EGF; 20 ng/ml), and fibroblast growth factor (FGF; 10 ng/ml) (Miltenyi Biotec).

In vitro kinase assays

K^{D}ELECT assays were conducted for CDK6 as performed in the commercial assay service by DiscoverX in a 11-point dose response. All K_Ds were tested in duplicate. For most assays, kinase-tagged T7 phage strains were prepared in an *E. coli* host derived from the BL21 strain. *E. coli* were grown to log-phase and

i

n

f

detection. Streptavidin-coated magnetic beads were treated with biotinylated small molecule ligands for

e

c

t

e

30 minutes at room temperature to generate affinity resins for kinase assays. The liganded beads were blocked with excess biotin and washed with blocking buffer (SeaBlock (Pierce), 1% BSA, 0.05% Tween 20, 1 mM DTT) to remove unbound ligand and to reduce non-specific binding. Binding reactions were assembled by combining kinases, liganded affinity beads, and test compounds in 1x binding buffer (20% SeaBlock, 0.17x PBS, 0.05% Tween 20, 6 mM DTT). Test compounds were prepared as 111X stocks in 100% DMSO. Kds were determined using an 11-point 3-fold compound dilution series with three DMSO control points. All compounds for Kd measurements are distributed by acoustic transfer (non-contact dispensing) in 100% DMSO. The compounds were then diluted directly into the assays such that the final concentration of DMSO was 0.9%. All reactions performed in polypropylene 384-well plate. Each was a final volume of 0.02 ml. The assay plates were incubated at room temperature with shaking for 1 hour and the affinity beads were washed with wash buffer (1x PBS, 0.05% Tween 20). The beads were then resuspended in elution buffer (1x PBS, 0.05% Tween 20, 0.5 μM nonbiotinylated affinity ligand) and incubated at room temperature with shaking for 30 minutes. The kinase concentration in the eluates was measured by qPCR.

Binding constants (Kds) were calculated with a standard dose-response curve using the Hill equation:

$$\text{Response} = \text{Background} + \frac{\text{Signal} - \text{Background}}{1 + (\text{Kd}^{\text{Hill Slope}} / \text{Dose}^{\text{Hill Slope}})}$$

The Hill Slope was set to -1. Curves were fitted using a non-linear least square fit with the Levenberg-Marquardt algorithm.

<u>KINOME*scan*™ Profiling</u> was service by DiscoverX. For most assays, kinase-tagged T7 phage strains were grown in parallel in 24-well blocks in an *E. coli* host derived from the BL21 strain. *E. coli* were grown to log-phase and infected with T7 phage from a frozen stock (multiplicity of infection = 0.4) and

i

n

c

u

b

a

t (SeaBlock (Pierce), 1 % BSA, 0.05 % Tween 20, 1 mM DTT) to remove unbound ligand and to reduce

e non-specific phage binding. Binding reactions were assembled by combining kinases, liganded affinity

d beads, and test compounds in 1x binding buffer (20 % SeaBlock, 0.17x PBS, 0.05 % Tween 20, 6 mM

w

i

t

DTT). Test compounds were prepared as 100x stocks in 100% DMSO and directly diluted into the assay. All reactions were performed in polypropylene 384-well plates in a final volume of 0.02 ml. The assay plates were incubated at room temperature with shaking for 1 hour and the affinity beads were washed with wash buffer (1x PBS, 0.05 % Tween 20). The beads were then re-suspended in elution buffer (1x PBS, 0.05 % Tween 20, 0.5 µM non-biotinylated affinity ligand) and incubated at room temperature with shaking for 30 minutes. The kinase concentration in the eluates was measured by qPCR.

JHK-02-108-2 was screened at the concentration of 1 µM, and results for primary screen binding interactions are reported as '% Ctrl', where lower numbers indicate stronger hits in the matrix.

%Ctrl Calculation:

$$\left(\frac{\text{test compound signal - positive control signal}}{\text{negative control signal - positive control signal}}\right) \times 100$$

negative control = DMSO (100%Ctrl), positive control = control compound (0%Ctrl)

Western blotting analysis

Total cells lysates were prepared by pelleting cells from each cell line at 4 °C (500 g) for 5 minutes. The resulting cell pellets were washed once with ice-cold 1x PBS and then resuspended in RIPA Lysis and Extraction Buffer (Thermo Fisher Scientific) supplemented with protease and phosphatase cocktails (Roche). Lysates were clarified at 20,000 g for 15 minutes at 4 °C. Protein concentrations were determined by BCA protein assay (Pierce). Lysate samples were prepared in 4× sample loading buffer (Bio-Rad), boiling for 10 min at 95 °C. Samples were loaded into 4-20% precast polyacrylamide gels (Bio-Rad) and separated by electrophoresis. The gels were transferred to polyvinylidene fluoride membranes (Millipore) activated with 100% methanol. The membranes were blocked for 1 hour at room temperature in Intercept (TBS) Blocking Buffer (LI-COR), and subsequently probed with appropriate primary antibodies [CDK6 (Cell Signaling Technology, #3136) CDK5 (Cell Signaling Technology, #12134), CDK2 (Cell Signaling Technology, #18048), LCK (Cell Signaling Technology, #2657), CRBN (Novus Biologicals, #NBP1-91810), β-Actin (Cell Signaling Technology, #3700)] at 4 °C overnight and then incubated with IRDye®800- labeled goat anti-rabbit IgG (LI-COR Biosciences, cat. no. 926-32211) or IRDye 680RD goat anti-Mouse IgG (LI-COR Biosciences, cat. no. 926-68070) secondary antibodies at room temperature for 1 hour. The membranes were detected on Li-COR Odyssey CLx system.

Cell viability assay (CellTiter-Glo assay)

Cell viability was evaluated using the CellTiter-Glo assay (CTG; Promega, Madison, WI, USA) for MV4-11 cells. Drug treatments were dry-dispensed in half-log dilutions into 384 well plates (Corning 3603) using a D300 digital drug dispenser (Hewlett Packard). Briefly, 10^5 MV4-11 cells in 50 µl per well were seeded in drug-containing 384-well plates (Corning) and incubated. After 72 h, the plates were subjected to CellTiter-Glo (Promega) as described in the manufacturer's manual. Luminescence was measured with a Synergy H1 (BioTek) plate scanner. The proliferation assays were performed in biological triplicate. IC50 values were determined using a non-linear regression curve fit in GraphPad PRISM 9.5.1.

Microscopy-based viability assays

SYTOX viability assays

OCI-AML-2, NOMO1, U937 and MOLM13 AML cell lines were plated in drug-containing 384 well plates as described above for the CTG assay at 4000 cells/well in 50 µl of media and incubated for 72h. 10 µl of a 6x solution of Hoechst 33342 (final concentration 1:5000; Thermo Fisher) and SYTOX Green Nucleic Acid Stain (final concentration 100 nM) in PBS was added per well and the contents were mixed using a liquidator multi-channel pipette and plates were returned to 37˚C for a minimum of one hour. Image acquisition was performed with an Image Xpress Micro-Confocal microscope (IXM-C; Molecular Devices). Four fields of view were imaged per well with a 10x objective. Nuclei were segmented using MetaXpress software and the SYTOX intensity was measured within each nuclear mask. Dead cells were gated manually based nuclear SYTOX. Live cell counts were used to calculate growth rate inhibition values (GR) and relative viabilities. Since GR values and relative viabilities led to the same conclusions, relative viabilities were used so that these dose responses could be compared to the CTG results in MV411 cells.

Dye Drop viability assays

GBM cell lines were plated as single cell suspensions 500-2000 cells per well in 60 µl of media in laminin coated 384 well CellCarrier plates (Perkin Elmer). Cells were allowed to adhere for 72h prior to treatment with the D300 drug dispenser (Hewlett Packard). Following 72 or 96h in drug, cells were fixed according to the Deep Dye Drop protocol.[2] Briefly, cells were stained with Live/Dead far red fluorescent dye (LDR; 1:2000 final dilution; Thermo Fisher) and pulsed with EdU (Lumiprobe) in a 10% solution of OptiPrep™ (Sigma) in PBS for 1 h prior to fixation with 4% formaldehyde, prepared in 20% OptiPrep™ for 30 min. Wells were aspirated and cells were then permeabilized with 0.5% Triton X-100 (Sigma) in 10% OptiPrep™ for 20 min. at RT, and the EdU was then labeled with cy3-azide (Lumiprobe) by Click chemistry in 20% OptiPrep™ for 30 min. at RT. Following aspiration, cells were stained overnight in

Hoechst 33342 (1:5000 in PBS; Thermo Fisher). Cells were washed three times with PBS and imaged on an Image Xpress Micro-Confocal microscope (IXM-C; Molecular Devices). All wash steps were performed with an eL406 automated plate washer (BioTek). Four fields of view were acquired with a 10x objective for each well. Nuclear segmentation, and feature extraction were performed with MetaXpress software (Molecular Devices) and growth rate and cell cycle analyses were performed using custom python scripts (https://github.com/datarail/DrugResponse/wiki) as previously described.[2-3]

Immunofluorescence and Cell Cycle Analysis

6-8x10^5 AML cells were plated into drug-containing 96-well plates (Corning) and incubated for 24 h. Cells were then fixed with 4% formaldehyde in 10% OptiPrep™ for 30 min. at room temperature (RT). Cells were transferred to v-bottom plates and centrifuged. Supernatant was discarded and cells were resuspended in 0.5% Triton-X in PBS and incubated for 15 min., centrifuged, and then resuspended in Intercept blocking buffer (Li-COR) and incubated for 1 h at RT. Cells were then centrifuged, and resuspended in antibody (1:200 CDK6; Cell Signaling Technologies 13331 or 1:400 phospho-RB (serine 780); Cell Signaling Technologies 8180) prepared in Intercept with Hoechst 33342 (1:5000) for overnight incubation at 4˚C. Cells were then washed once with PBST (PBS with 0.1% Tween 20), and twice with PBS with centrifugation between each step. Cells were then resuspended in donkey anti-rabbit Alexa 647 secondary antibody (Thermo Fisher) at 1:2000 prepared in Intercept, and incubated for 1 h. Cells were washed once in PBST and twice in PBS and transferred to 384w CellCarrier ULTRA plates (Perkin Elmer) for imaging. Image acquisition was performed on an Image Xpress Micro-Confocal (IXM-C) high throughput microscope (Molecular Devices), nuclei were segmented using Otsu thresholding in CellProfiler, or using Spot Detection in MetaXpress (Molecular Devices) and the integrated intensity of the Hoechst signal within the nuclear masks was used to measure DNA content. Manual gates were applied to classify cells as antibody positive or negative and identify G1 and G2 cell cycle phases. The median intensity of nuclear CDK6 was calculated.

NanoBRET experiment

HEK293 cells were plated 6-well plates containing 8 x 10^5 cells/well and allowed to synchronize for 4-6 hours at 37 °C, 5% CO_2. In 200µl Opti-MEM reduced serum medium, 4 µg of HT acceptor CRBN-HaloTag vector and 0.2µg of diluted NanoLuc donor CDK6 vector were combined. For CDK5-CRBN engagement, 2 µg of HT acceptor CRBN-HaloTag vector and 0.2µg of diluted NanoLuc donor CDK5 vector were combined. 8 µl FuGENE® HD transfection reagent was added and cells were incubate for 20

minutes at room temperature. ~200 μL of the DNA/transfection reagent was then added to each well. Cells were then Incubated at 37 °C, 5% CO_2 incubator overnight. The transfected cells were trypsinized and re-plated into a 384-well plate in replicates in FluoroBrite™ DMEM (+ 10% FBS) at a density of ~20,000 cells/well. Cells were incubated overnight at 37 °C in 5% CO_2 incubator. Next day, MLN4924 1 μM was pretreated for 2 h, then 100 nM HaloTag NanoBRET 618 ligand is treated and incubated followed by treating each compound right after HaloTag NanoBRET 618 treatment. After incubating for 2 h, 10 μL of 1:1000 diluted NanoBRET substrate in serum free FluoroBrite™ DMEM is treated and plate was read immediately.

Flow cytometry analysis for reporter assay

K562-Cas9 cells expressing the $CDK6_{eGFP}$ or $CDK5_{eGFP}$ dual-colour degradation reporters were resuspended at 0.7×10^6 ml^{-1} and 50 μl of cell suspension was seeded in 384-well plates. Shortly after, cells were treated with DMSO (n=2) or drug (n=2) for 16 h. The indicated drugs were dispensed with a D300 digital dispenser (Tecan). The fluorescent signal was quantified by flow cytometry (FACSymphony flow cytometer, BD Biosciences). Using FlowJo (flow cytometry analysis software, BD Biosciences), the geometric mean of the eGFP and mCherry fluorescent signal for round and mCherry-positive cells was calculated. The ratio of eGFP to mCherry was normalized to the average of two DMSO-treated controls. Dose-response curves were generated using Prism 10 (GraphPad), and IC50 values were calculated accordingly.

Constructs

Expression constructs generated for this study were prepared by standard molecular biology techniques and coding sequences entirely verified. Constructs for bacterial expression of the NEDD8 E1 APPBP1-UBA3, UBE2M, and NEDD8 were previously described.[4] UBE2D3 was cloned into a pGEX-4T1-TEV based vector. cDNA constructs for CUL4, DDB1, CRBN, and CDK6 were obtained from Open Biosystems. CUL4A (residues 38-C), His-TEV-RBX1 (residues 5-C), full-length DDB1, GST-TEV-CRBN, and His-TEV-CDK6 were first cloned into pLib via Gibson assembly. A single expression vector for CUL4A-RBX1 and DDB1-CRBN were generated by preparing cassettes from the relevant pLib vectors via PCR as described,[5] and Gibson assembled into pBig1a. Proper assembly into pBig1a was confirmed by PmeI and SwaI restriction digestion

Protein expression and purification

UBE2M and the NEDD8 E1 APPBP1-UBA3 were expressed in *E. coli* BL21 Gold (DE3) cells as GST fusion proteins. Fusion proteins were purified from cell lysates by glutathione affinity chromatography and liberated from GST by thrombin cleavage overnight at 4 °C. Cleavage reactions were further purified by ion-exchange and size exclusion chromatography in 25 mM HEPES, 200 mM NaCl, 1 mM DTT pH 7.5 (Buffer A). NEDD8 was expressed in *E. coli* BL21 Gold (DE3) cells as GST- fusion protein, purified from cell lysates by glutathione affinity chromatography, and liberated from GST by thrombin cleavage during extensive dialysis overnight in Buffer A at 4 °C. Cleavage reactions were passed back over a glutathione affinity resin to remove free GST and any remaining uncleaved GST-fusion protein. Protein collected in the flow fraction was concentrated with an Amicon Ultra filtration unit and further purified by size exclusion chromatography in Buffer A.

CUL4A-RBX1 was co-expressed in insect cells as a His-TEV-RBX1 fusion protein with untagged CUL4A and CDK6 was expressed as a His-TEV-CDK6 fusion protein. Proteins were purified from cell lysates by Ni affinity chromatography. Following TEV cleavage overnight at 4 °C cleavage reactions were further purified by ion exchange and size exclusion chromatography in Buffer A. DDB1-CRBN was co-expressed in insect cells as a GST-TEV-CRBN fusion protein with untagged DDB1. Fusion proteins were purified from cell lysates by glutathione affinity chromatography and liberated from GST by TEV cleavage overnight at 4 °C. Cleavage reactions were further purified by ion-exchange and size exclusion chromatography in buffer A.

To introduce a cysteine for fluorescent labeling of ubiquitin (UB) we mutated the protein kinase a site in the pGEX-2TK backbone converting the PKA site from RRASV to RRACV.[6] UB purified from this expression construct was labeled with AlexaFluor 488 Maleimide as previously described.[6] Briefly, DTT was added to UB at a final concentration of 10 mM and incubated on ice for 20 minutes to completely reduce cysteines for labeling. DTT was removed by buffer exchange over a NAP-5 column (GE Healthcare) in labeling buffer (25 mM HEPES, 200 mM NaCl). Labeling reactions consisted of UB at 150 mM final concentration and was initiated by the addition of 600 mM AlexaFluor 488 Maleimide (4X excess over labeling target and <5% final DMSO concentration). Reactions were incubated at room temperature for 2 hours and quenched by the addition of DTT to 10mM. Quenched reactions were desalted over a PD-10 column in labeling buffer containing 1mM DTT to remove unreacted probe.

Desalted protein was concentrated in an Amicon Ultra filtration unit and further purified over a Sephadex SD75 column.

Neddylated CUL4A-RBX1 was prepared by mixing 8 μM CUL4A-RBX1, 1 μM UBE2M, 0.1 μM APPBP1-UBA3, and 20 μM NEDD8 in 25mM HEPES, 200mM NaCl, 10mM $MgCl_2$, 1mM ATP, pH = 7.5. Reactions were initiated at room temperature by the addition of NEDD8 and incubated for ten minutes prior to quenching by the addition of DTT to 10 mM. Quenched reactions were spun at 17K xg for 10 minutes and immediately applied to a Superdex SD200 column in buffer A to purify NEDD8-CUL4A-RBX1 away from reaction components

Biochemical Assays

Ubiquitination assays were performed in pulse-chase format following the path of fluorescent ubiquitin from UBE2D3 to substrate. First, UBE2D3 was pulse-labeled by incubating a mixture of UBA1 (0.3 μM), UBE2D3 (10 μM), and fluorescently labeled UB (15 μM) in 25 mM HEPES, 100 mM NaCl, 100mM $MgCl_2$, ATP (2 mM), pH 7.5 at room temperature for 12 minutes to generate the UBE2D3~UB thioester intermediate (~ refers to covalent complex, thioester bonded between UB C-terminus and UBE2D3 catalytic cysteine). Pulse-loading reactions were quenched by the addition of EDTA to 25 mM and incubated on ice for 5 minutes. Chase reactions consisted of mixing the UBE2D3~UB thioester conjugate (0.3 μM final concentration) with pre-equilibrated NEDD8-$CRL4^{CRBN}$ (0.2 μM final concentration) with or without the indicated molecules (0.3 μM final concentration) and the indicated concentration of CDK6 in 25 mM HEPES, 100 mM NaCl, 0.5 mg/ml BSA, pH 7.5 at room temperature. Reactions were quenched at the indicated times with 2X SDS-PAGE sample buffer. Quenched samples were separated on 4-12% Bis-Tris gradient gels and scanned for fluorescence on a Typhoon imager.

TR-FRET assay

50 nM terbium-labeled CDK6 and 150 nM unlabeled CDK6 were mixed with 200 nM eGFP-CRBN/DDB1ΔB in 30 mM HEPES pH 7.4, 200 mM NaCl, 0.05% Tween-20, 0.5% BSA, and 2 mM TCEP. 15 μL of the mixture was added per well to a 384-well microplate. Compounds were titrated at the

indicated concentration using a D300e Digital Dispenser (HP). Reactions were incubated for 1 hour at room temperature prior to being measured on a PHERAstar FS microplate reader (BMG Labtech). TR-FRET signal was calculated by averaging the 520 nm/490 nm signal over 5 cycles.

Sample preparation LFQ quantitative mass spectrometry

Cells were lysed by addition of lysis buffer (8 M Urea, 50 mM NaCl, 50 mM 4-(2-hydroxyethyl)-1-piperazineethanesulfonic acid (EPPS) pH 8.5, Protease and Phosphatase inhibitors) and homogenization by bead beating (BioSpec) for three repeats of 30 seconds at 2400 strokes/min. Bradford assay was used to determine the final protein concentration in the clarified cell lysate. Fifty micrograms of protein for each sample was reduced, alkylated and precipitated using methanol/chloroform as previously described,[7] and the resulting washed precipitated protein was allowed to air dry. Precipitated protein was resuspended in 4 M urea, 50 mM HEPES pH 7.4, followed by dilution to 1 M urea with the addition of 200 mM EPPS, pH 8. Proteins were digested with the addition of LysC (1:50; enzyme:protein) and trypsin (1:50; enzyme:protein) for 12 h at 37 °C. Sample digests were acidified with formic acid to a pH of 2-3 before desalting using C18 solid phase extraction plates (SOLA, Thermo Fisher Scientific). Desalted peptides were dried in a vacuum-centrifuged and reconstituted in 0.1% formic acid for liquid chromatography-mass spectrometry analysis.

Data were collected using a TimsTOF Pro2 or HT (Bruker Daltonics, Bremen, Germany) coupled to a nanoElute or nanoElute2 LC pump (Bruker Daltonics, Bremen, Germany) via a CaptiveSpray nano-electrospray source. Peptides were separated on a reversed-phase C_{18} column (25 cm x 75 µm ID, 1.6 µM, IonOpticks, Australia) containing an integrated captive spray emitter. Peptides were separated using a 50 min gradient of 2 - 30% buffer B (acetonitrile in 0.1% formic acid) with a flow rate of 250 nL/min and column temperature maintained at 50 ºC.

To perform diaPASEF on the TimsTOF Pro2, the precursor distribution in the DDA *m/z*-ion mobility plane was used to design an acquisition scheme for Data-independent acquisition (DIA) data collection which included two windows in each 50 ms diaPASEF scan. Data was acquired using sixteen of these 25 Da precursor double window scans (creating 32 windows) which covered the diagonal scan line for doubly and triply charged precursors, with singly charged precursors able to be excluded by their position in the m/z-ion mobility plane. These precursor isolation windows were defined between 400 - 1200 *m/z* and 1/k0 of 0.7 - 1.3 V.s/cm^2.

To perform diaPASEF on the TimsTOF HT, we used py_diAID [8], a python package, to assess the precursor

distribution in the *m/z*-ion mobility plane to generate a diaPASEF acquisition scheme with variable window isolation widths that are aligned to the precursor density in m/z. Data was acquired using twenty cycles with three mobility window scans each (creating 60 windows) covering the diagonal scan line for doubly and triply charged precursors, with singly charged precursors able to be excluded by their position in the m/z-ion mobility plane. These precursor isolation windows were defined between 350 - 1250 *m/z* and 1/k0 of 0.6 - 1.45 V.s/cm^2.

Immunoprecipitation and sample preparation for immunoprecipitation mass spectrometry (IP-MS)

A total of 1 × 10^7 cells per IP were collected and lysed in lysis buffer (50 mM Tris pH 8, 200 mM NaCl, 2 mM TCEP, 0.1% NP-40, 10 units turbonuclease/200 µL buffer, 1x cOmplete protease inhibitor tablet/5 mL buffer) and sonicated on ice for 5 rounds of 2 seconds followed by 10 second pauses at 25% amplitude. After centrifugation clarification, the following steps were performed on an opentrons OT2 liquid handler. Cell lysate, 10µg of Flag-CRBN-DDB1ΔB, 1 µM of MLN4924 and CSN5i-3 (neddylation trap)[6], and 1 µM of selected degraders or DMSO vehicle control were transferred to PCR plate and incubated for 1 hour at 4 °C. 3 µL of pre-washed and resuspended Anti-Flag magnetic bead 25% slurry (Pierce) were added to the lysates followed by a second incubation for 1 hour at 4 °C. Beads were washed three times with wash buffer (50 mM Tris pH 8, 2 mM TCEP, 0.1% NP-40, 1x cOmplete protease inhibitor tablet/5 mL buffer) containing the required compounds, followed by three additional non-detergent (50 mM Tris pH 8, 2 mM TCEP, 1x cOmplete protease inhibitor tablet/5 mL buffer) washes containing the required compounds. After the final wash step, samples were eluted using 0.1 M Glycine-HCl, pH 2.7. Tris (1M, pH 8.5) was added to elution to reach a pH of 8. Samples were then reduced with 10 mM TCEP for 30 min at room temperature, followed by alkylation with 15 mM iodoacetamide for 45 min at room temperature in the dark. Alkylation was quenched by the addition of 10 mM DTT. The resuspended protein samples were digested with 2 µg LysC and 1 µg Trypsin overnight at 37°C. Sample digests were acidified with formic acid to a pH of 2-3 prior to desalting using C18 solid phase extraction plates (SOLA, Thermo Fisher Scientific). Desalted peptides were dried in a vacuum-centrifuged and reconstituted in 0.1% formic acid for LC-MS analysis.

Data were collected using a TimsTOF Ultra2 (Bruker Daltonics, Bremen, Germany) coupled to a nanoElute2 LC pump (Bruker Daltonics, Bremen, Germany) via a CaptiveSpray nano-electrospray source. Peptides were separated on a reversed-phase C18 column (25 cm x 75 µM ID, 1.6 µM,

IonOpticks, Australia) containing an integrated captive spray emitter. Peptides were separated using a 30 min gradient of 2 - 30% buffer B (acetonitrile in 0.1% formic acid) with a flow rate of 250 nL/min and column temperature maintained at 50 ºC.

The TIMS elution voltages were calibrated linearly with three points (Agilent ESI-L Tuning Mix Ions; 622, 922, 1,222 *m/z*) to determine the reduced ion mobility coefficients ($1/K_0$). To perform diaPASEF, we used py_diAID [8], a python package, to assess the precursor distribution in the *m/z*-ion mobility plane to generate a diaPASEF acquisition scheme with variable window isolation widths that are aligned to the precursor density in m/z. Data was acquired using twenty cycles with three mobility window scans each (creating 60 windows) covering the diagonal scan line for doubly and triply charged precursors, with singly charged precursors able to be excluded by their position in the m/z-ion mobility plane. These precursor isolation windows were defined between 350 - 1250 *m/z* and 1/k0 of 0.6 - 1.45 $V.s/cm^2$.

LC-MS data analysis

The diaPASEF raw file processing and controlling peptide and protein level false discovery rates, assembling proteins from peptides, and protein quantification from peptides were performed using either a targeted cell line specific spectral or using library free analysis searched with a Swissprot human database (January 2021) in DIA-NN 1.8.[10] Database search criteria largely followed the default settings for directDIA including: tryptic with two missed cleavages, carbamidomethylation of cysteine, and oxidation of methionine and precursor Q-value (FDR) cut-off of 0.01. Precursor quantification strategy was set to Robust LC (high accuracy) with RT-dependent cross run normalization.

Whole cell data: Proteins with low sum of abundance (<500-2,000 x no. of treatments) were excluded from further analysis and resulting data was filtered to only include proteins that had a minimum of 3 counts in at least 4 replicates of each independent comparison of treatment sample to the DMSO control.[11] IP data: Resulting data was filtered to only include proteins that had a minimum of 3 counts in at least 4 replicates of each independent comparison of treatment sample to the DMSO control. Protein abundances were globally normalized using in-house scripts in the R framework (R Development Core Team, 2014). Proteins with missing values were imputed by random selection from a Gaussian distribution either with a mean of the non-missing values for that treatment group or with a mean equal to the median of the background (in cases when all values for a treatment group are missing). Protein abundances were scaled and significant changes comparing the relative protein abundance of each treatment to DMSO control were assessed by two-sided moderated t-test as implemented in the limma package within the R

framework.[12]

3. NMR spectra

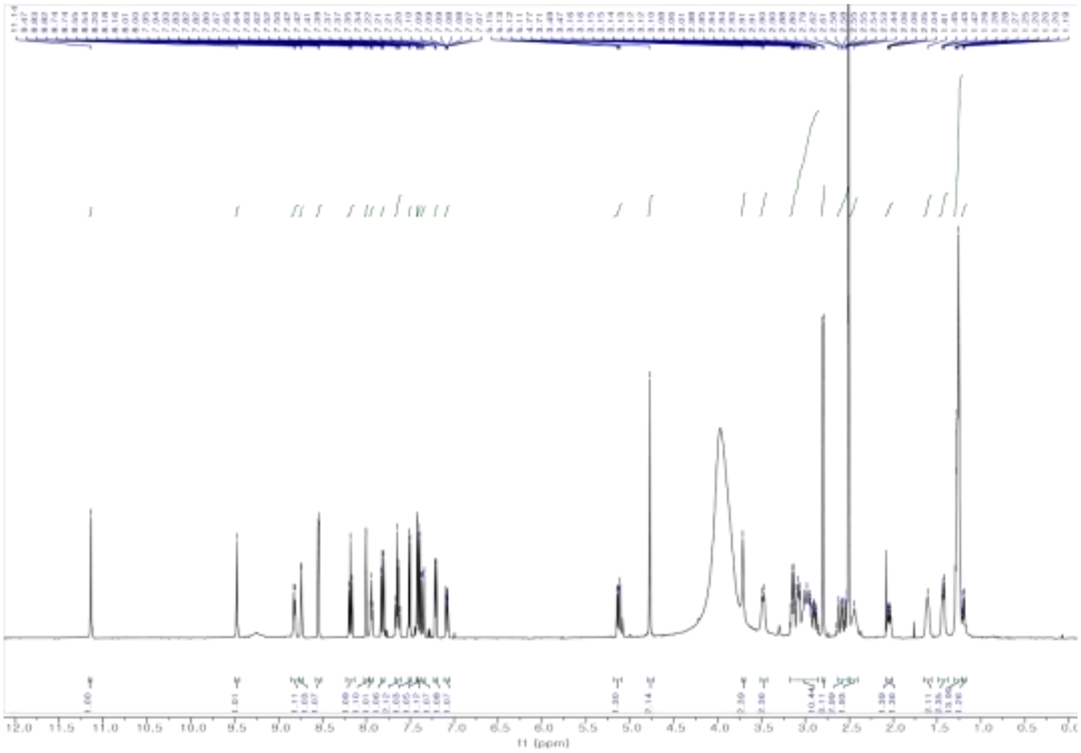

^{1}H NMR spectrum of **STT-03-123** in DMSO-d_6

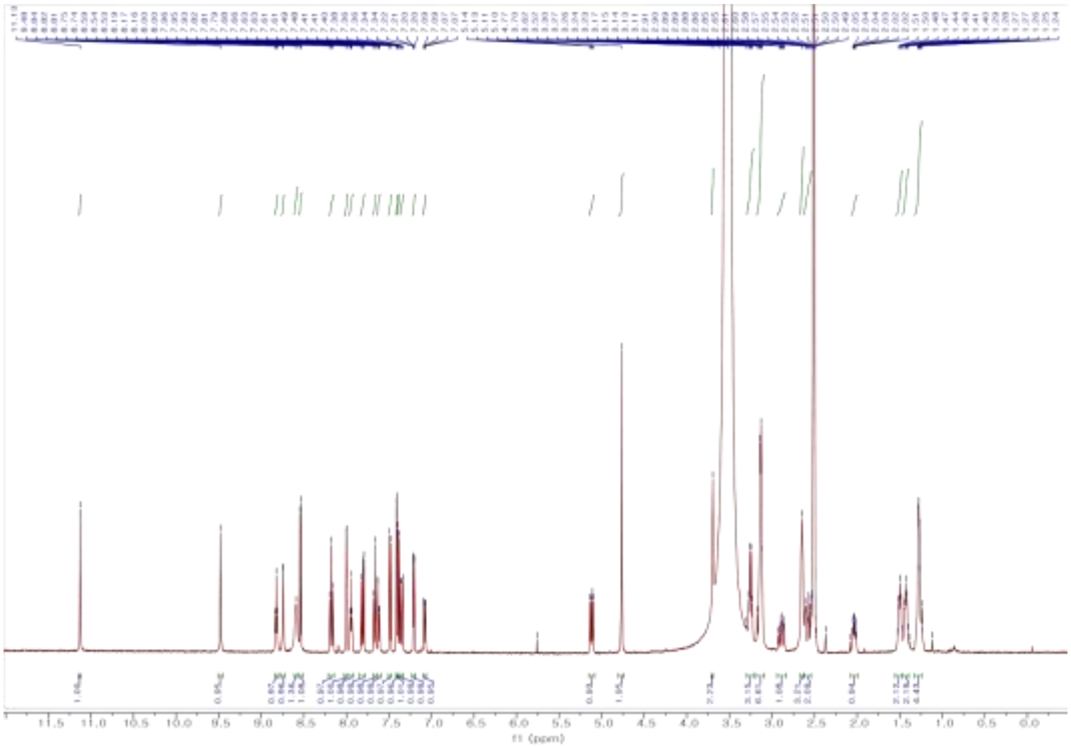

^{1}H NMR spectrum of **STT-03-122** in DMSO-d_6

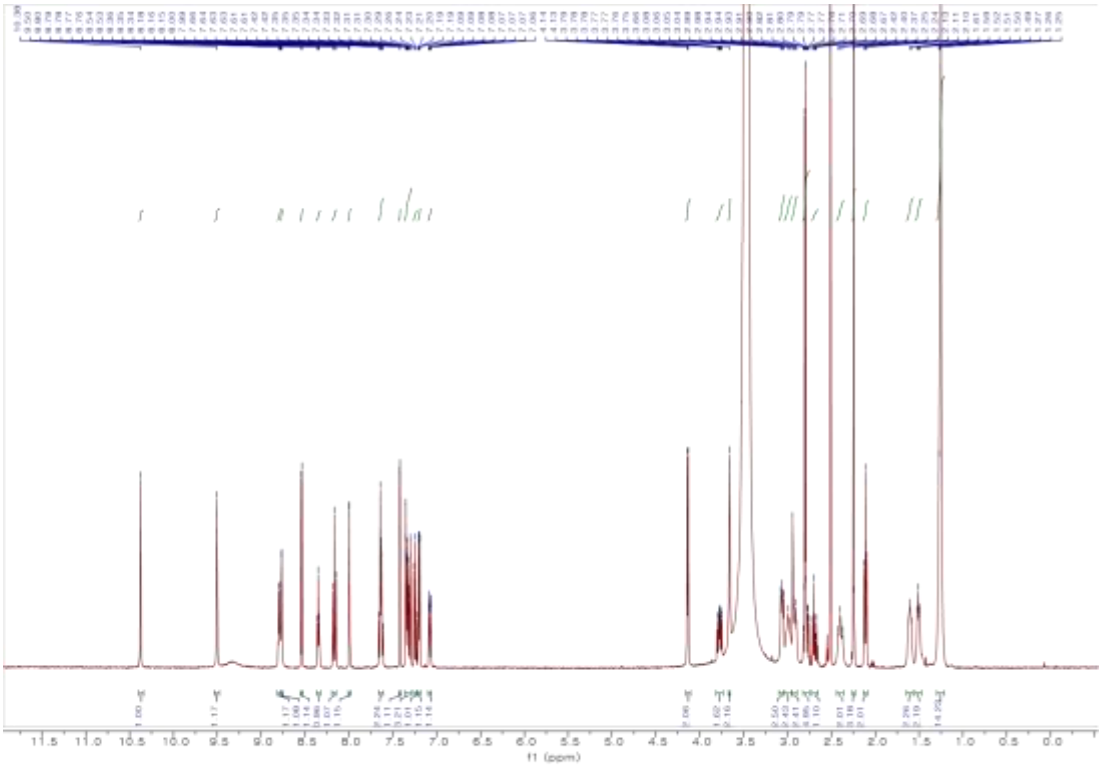

^{1}H NMR spectrum of **JHK-02-063-1** in DMSO-d_6

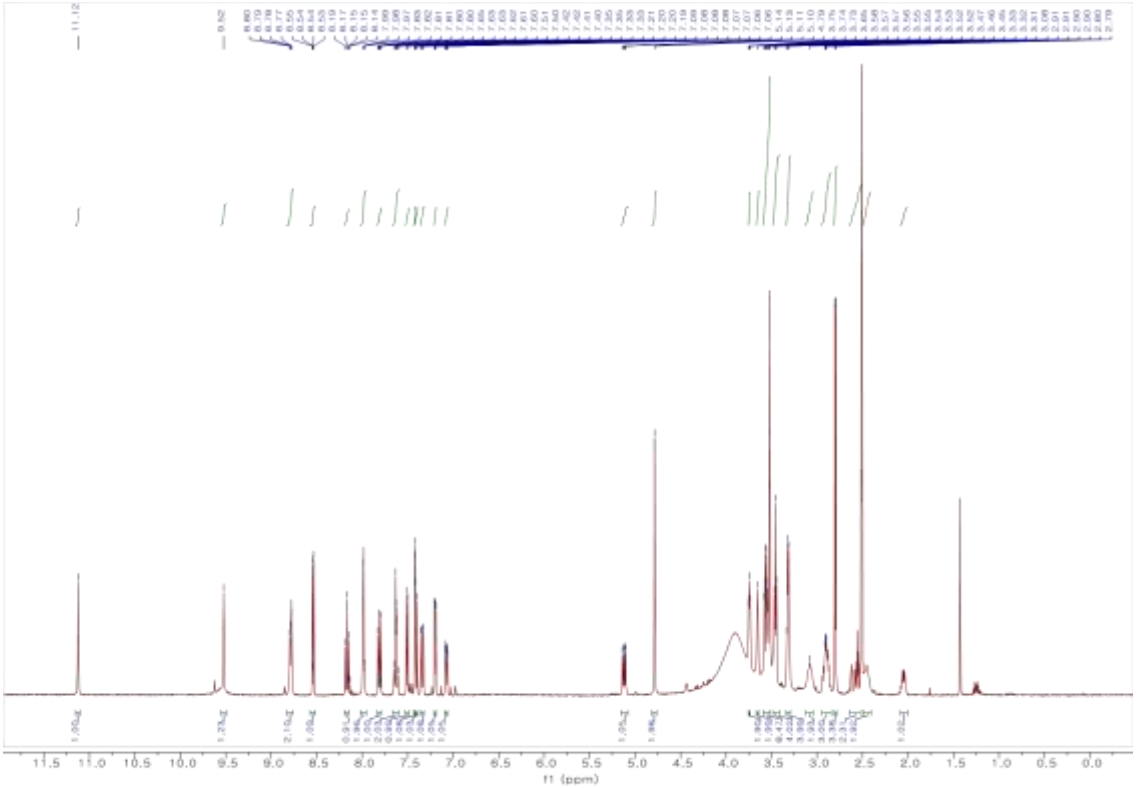

^{1}H NMR spectrum of **JHK-02-065-1** in DMSO-d_6

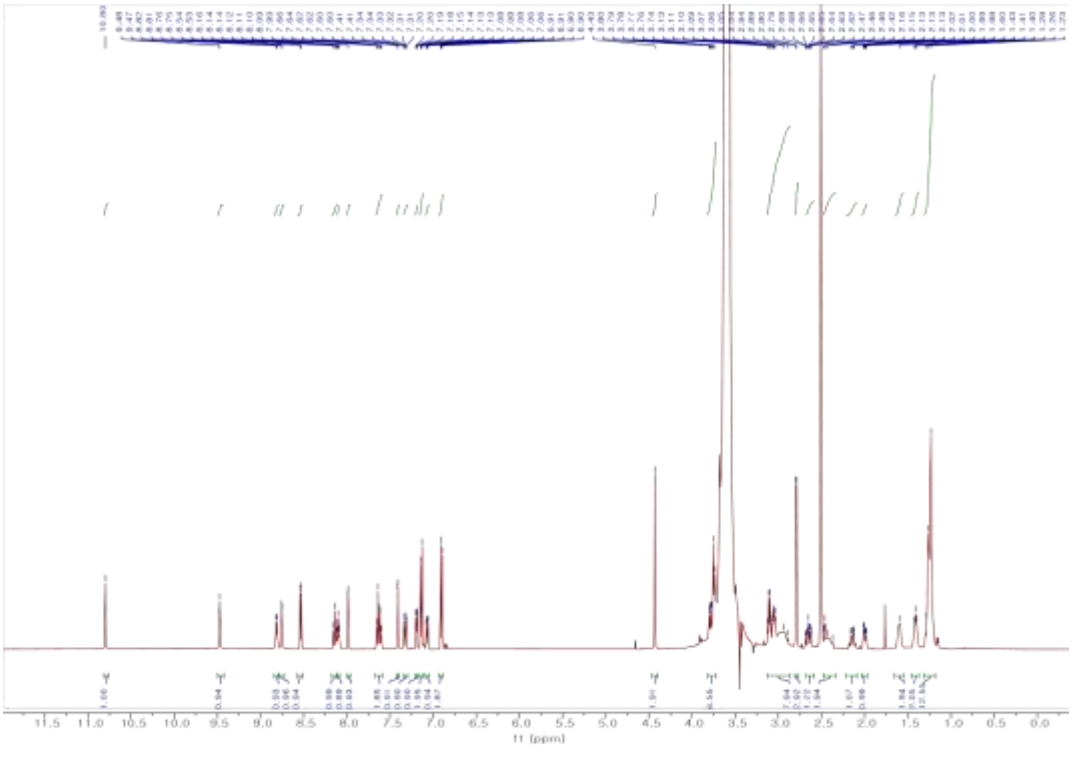

^{1}H NMR spectrum of **JHK-02-078-2** in DMSO-d$_6$

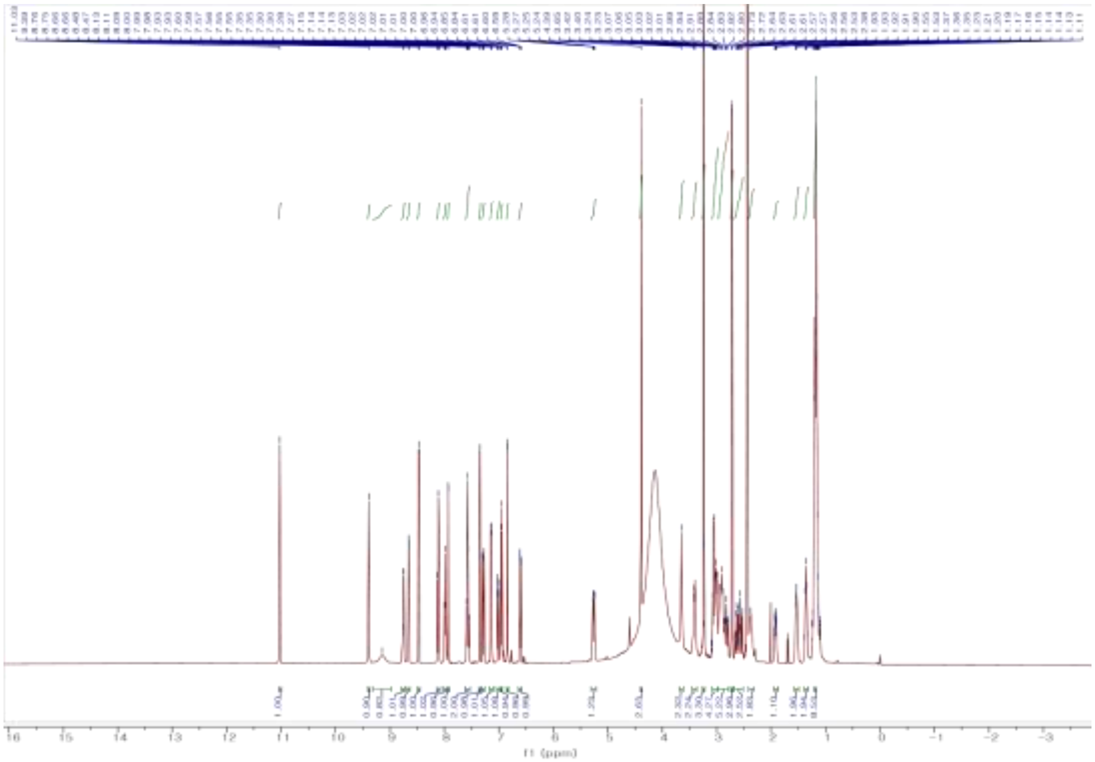

^{1}H NMR spectrum of **JHK-02-078-3** in DMSO-d$_6$

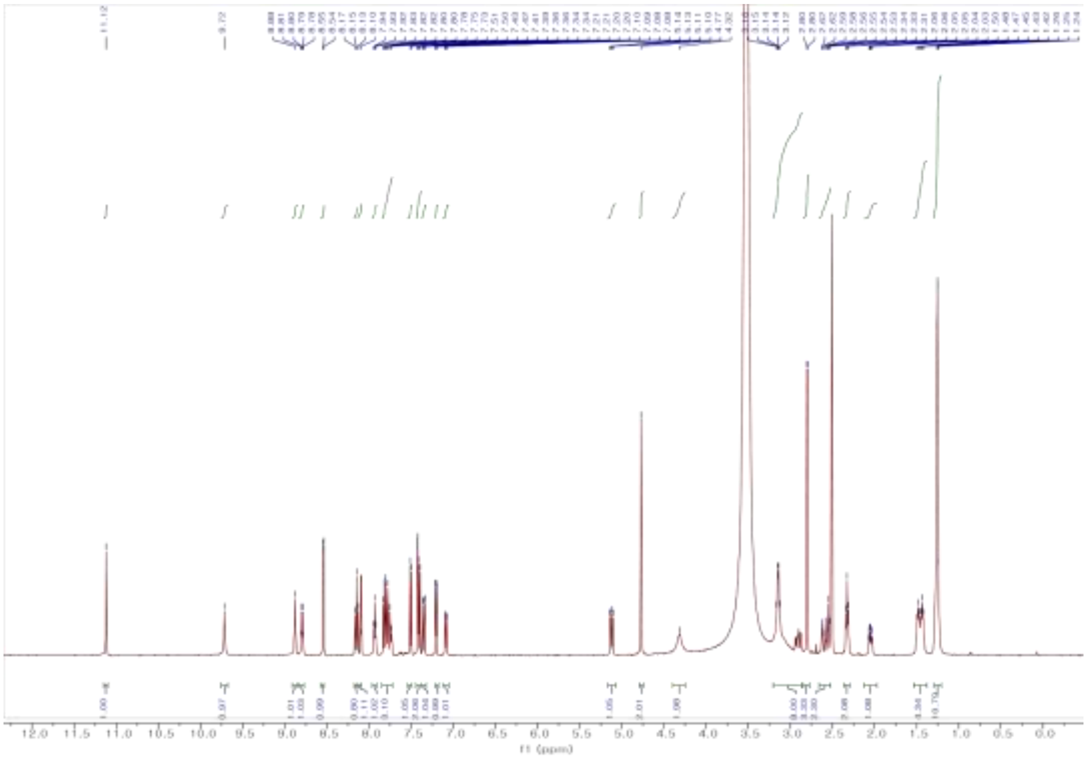

^{1}H NMR spectrum of **JHK-02-080-1** in DMSO-d_6

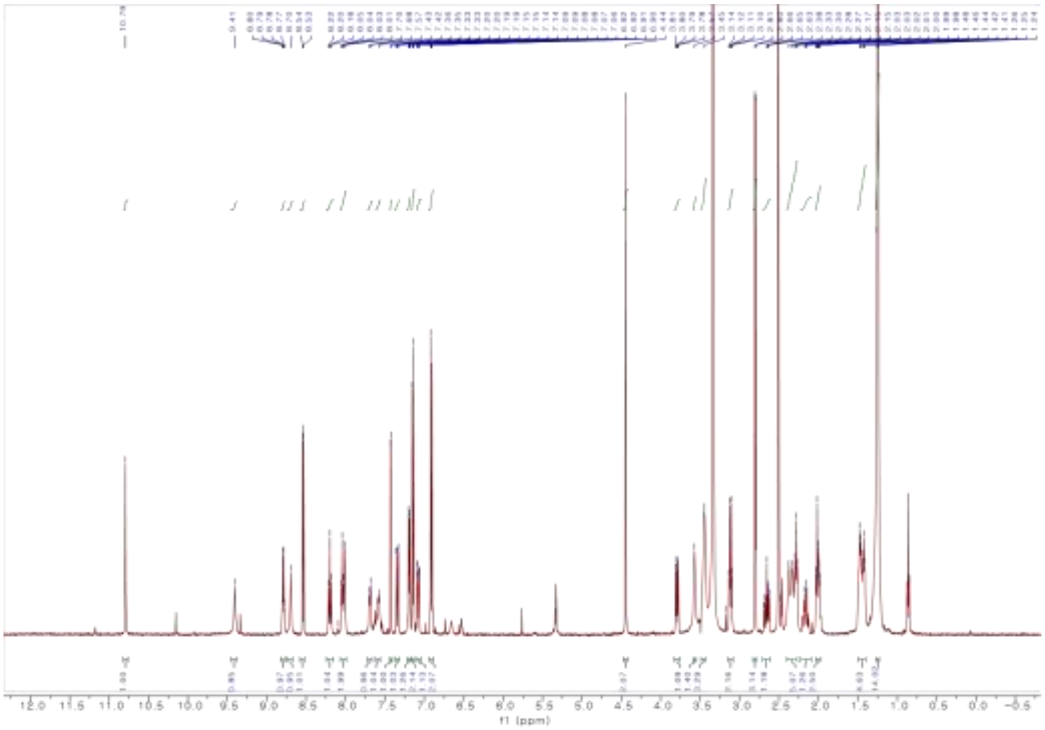

^{1}H NMR spectrum of **JHK-02-080-2** in DMSO-d_6

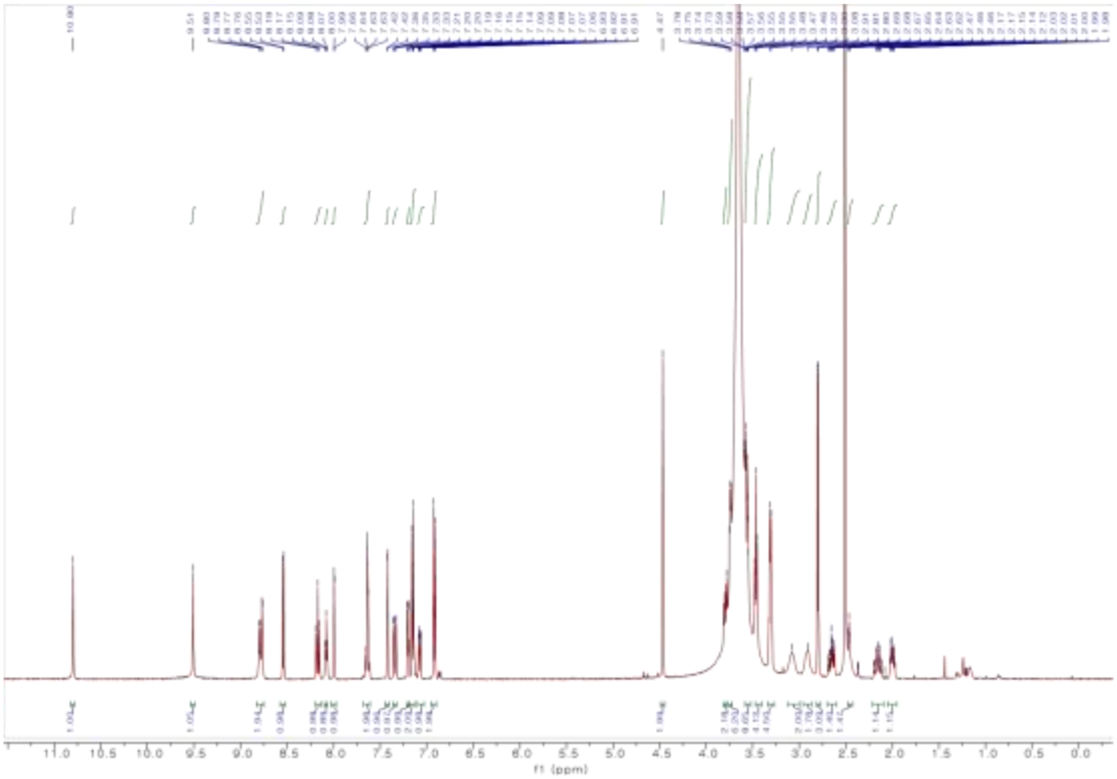

^{1}H NMR spectrum of **JHK-02-102-1** in DMSO-d_6

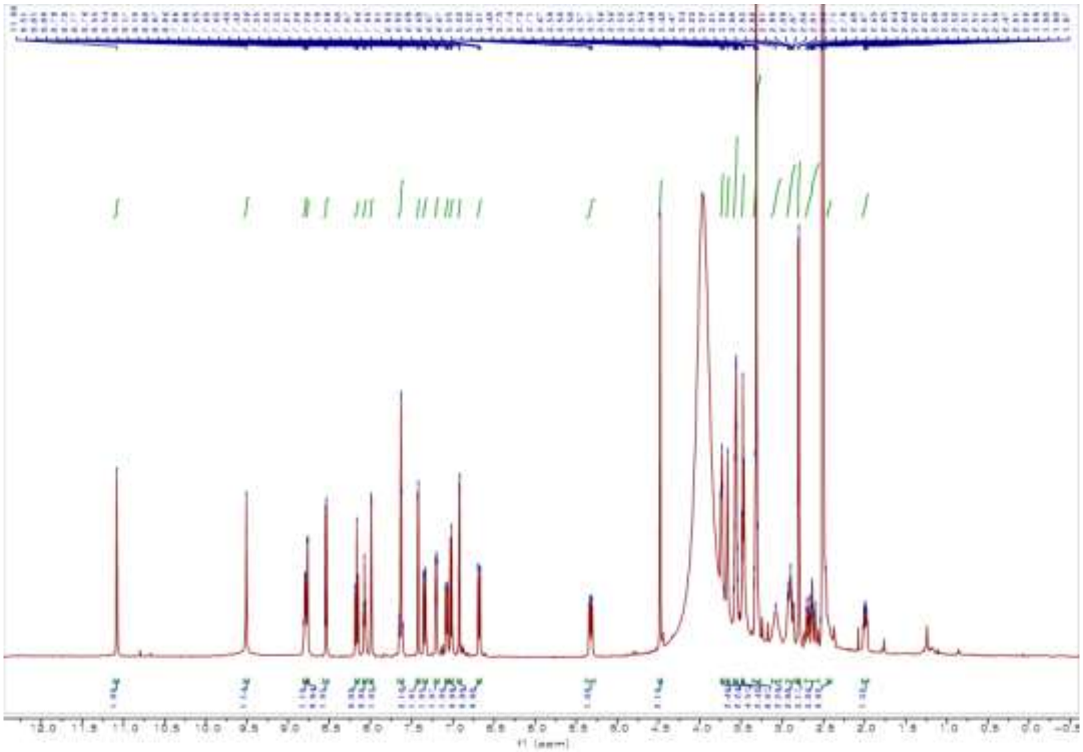

^{1}H NMR spectrum of **JHK-02-102-2** in DMSO-d_6

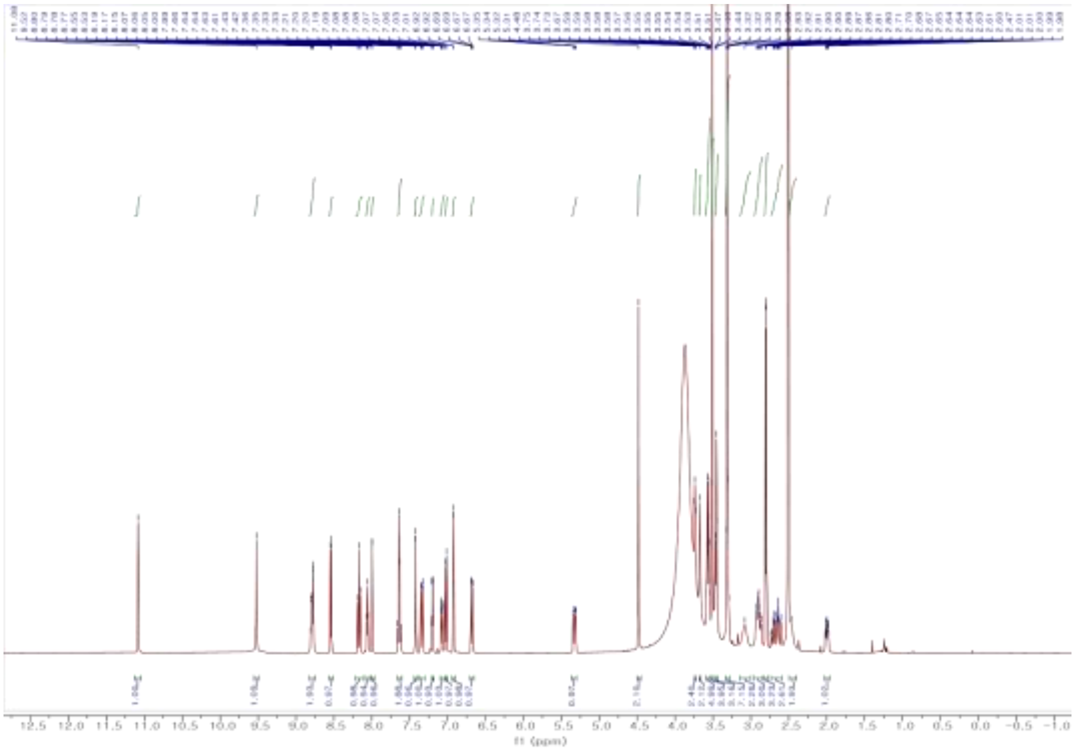

^{1}H NMR spectrum of **JHK-02-103-2** in DMSO-d_6

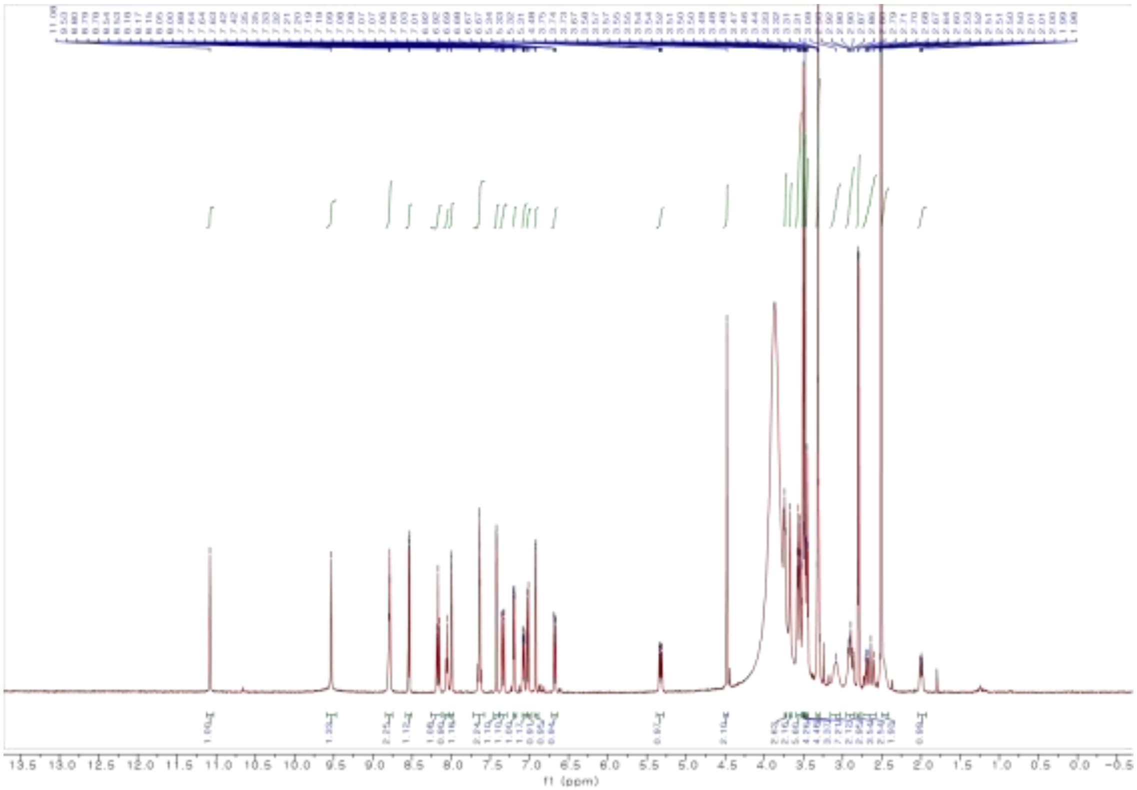

^{1}H NMR spectrum of **JHK-02-104-2** in DMSO-d_6

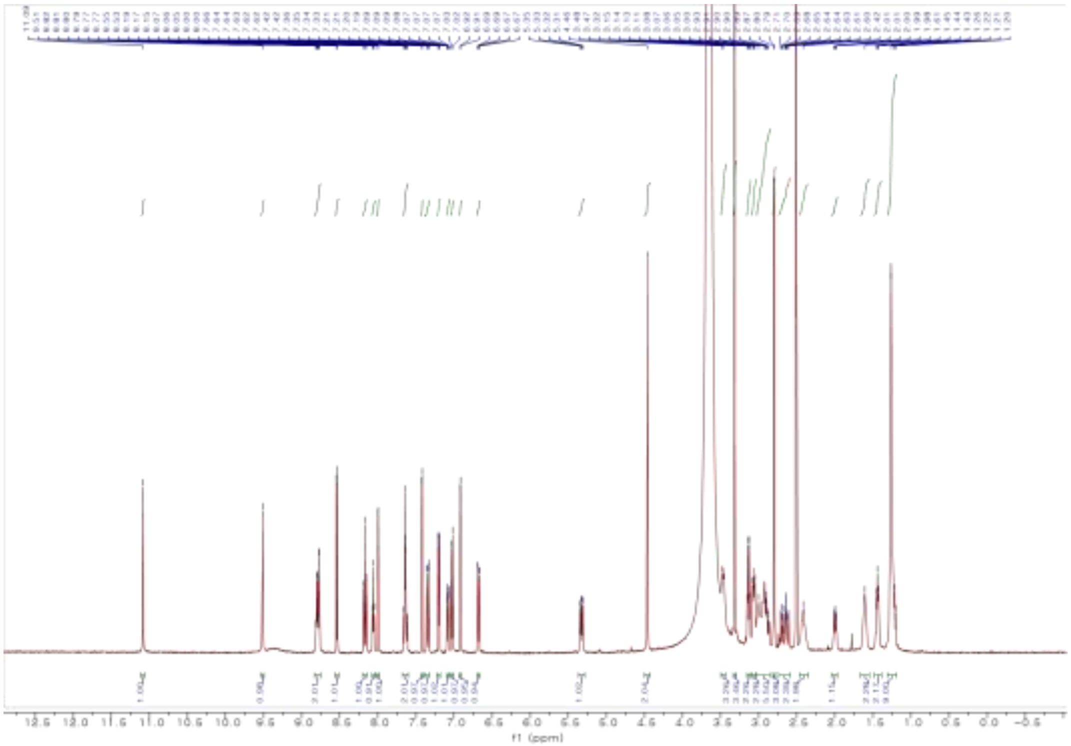

^{1}H NMR spectrum of **JHK-02-108-2** in DMSO-d_6

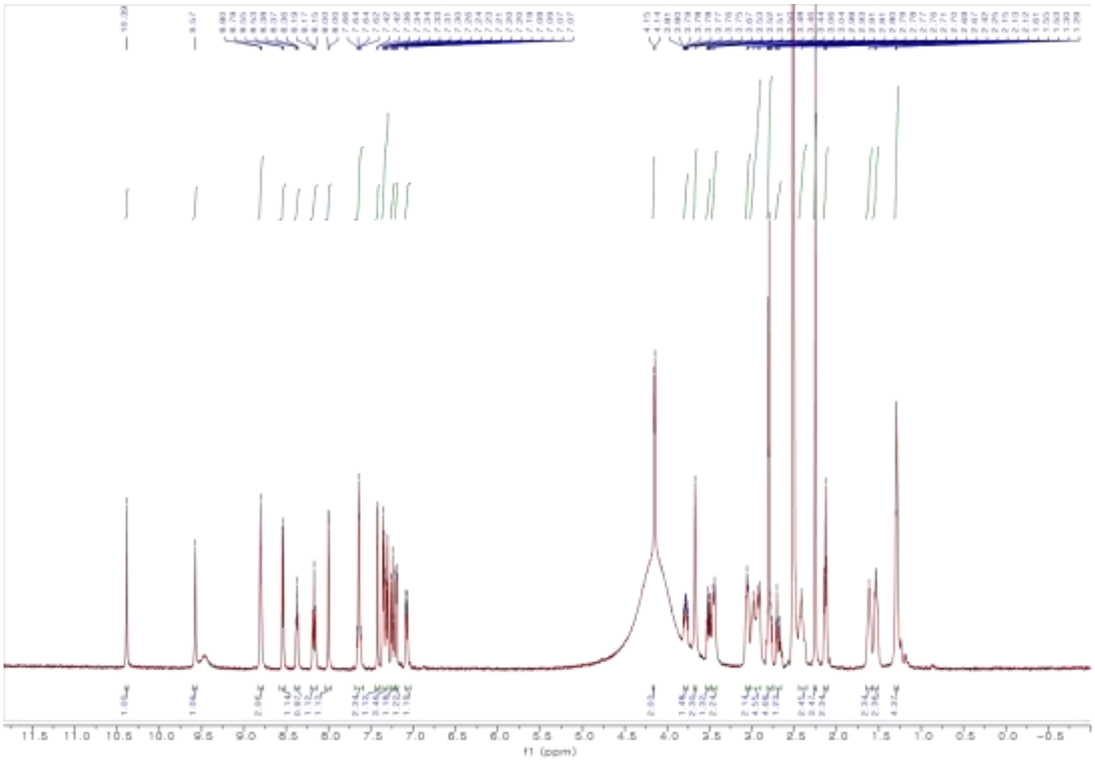

^{1}H NMR spectrum of **JHK-02-115** in DMSO-d_6

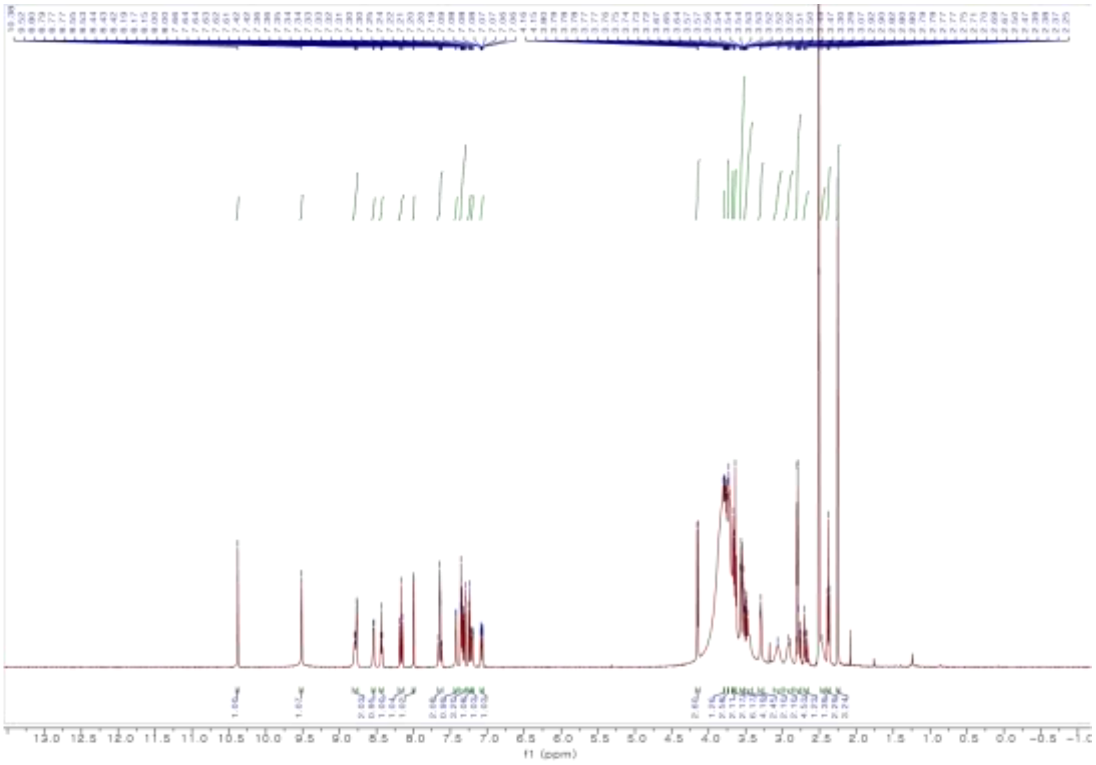

^{1}H NMR spectrum of **JHK-02-117** in DMSO-d_6

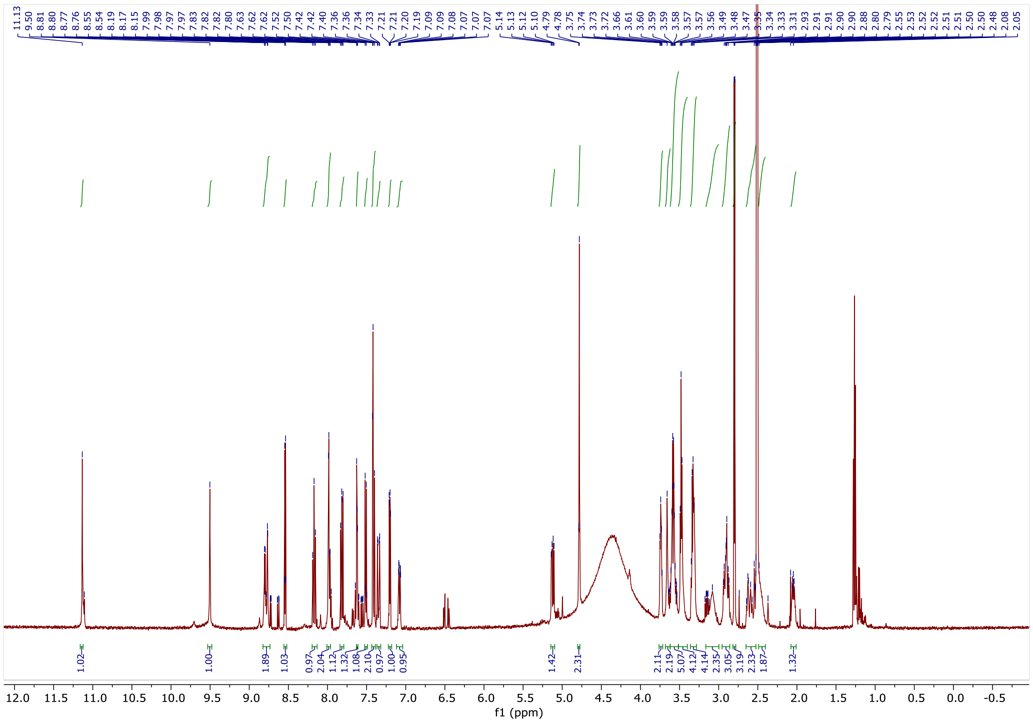

^{1}H NMR spectrum of **JHK-02-065-2** in DMSO-d_6

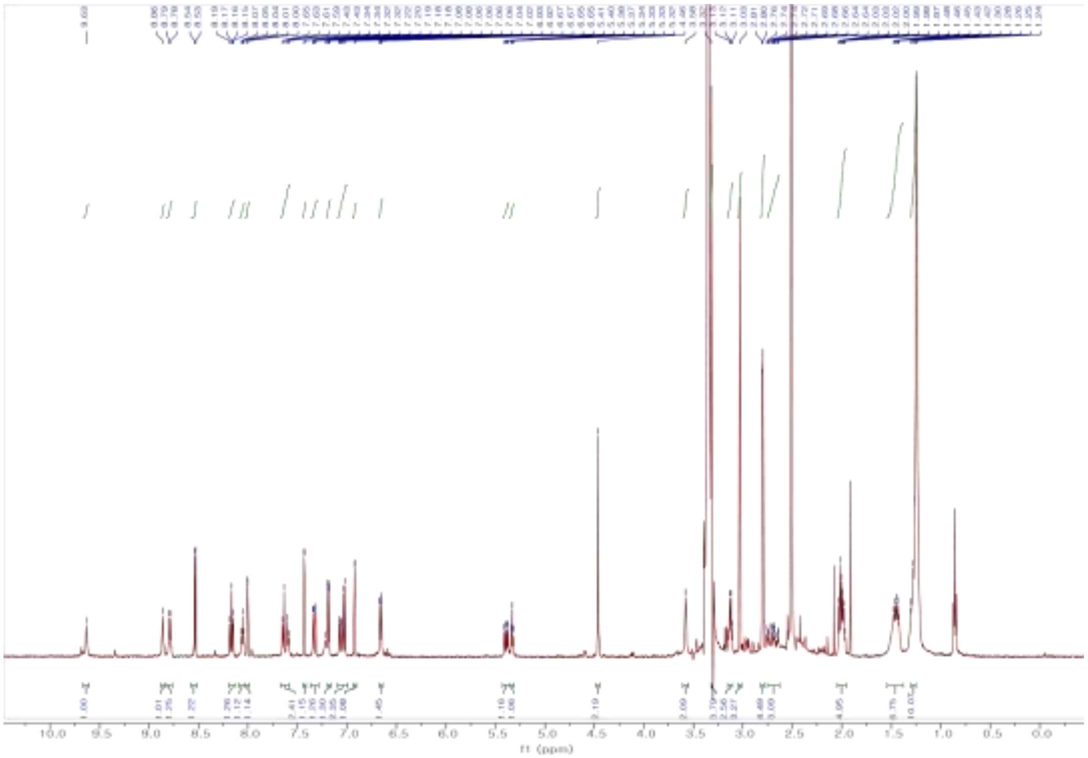

^{1}H NMR spectrum of **JHK-02-137 (JHK-02-108-2 neg)** in DMSO-d$_6$

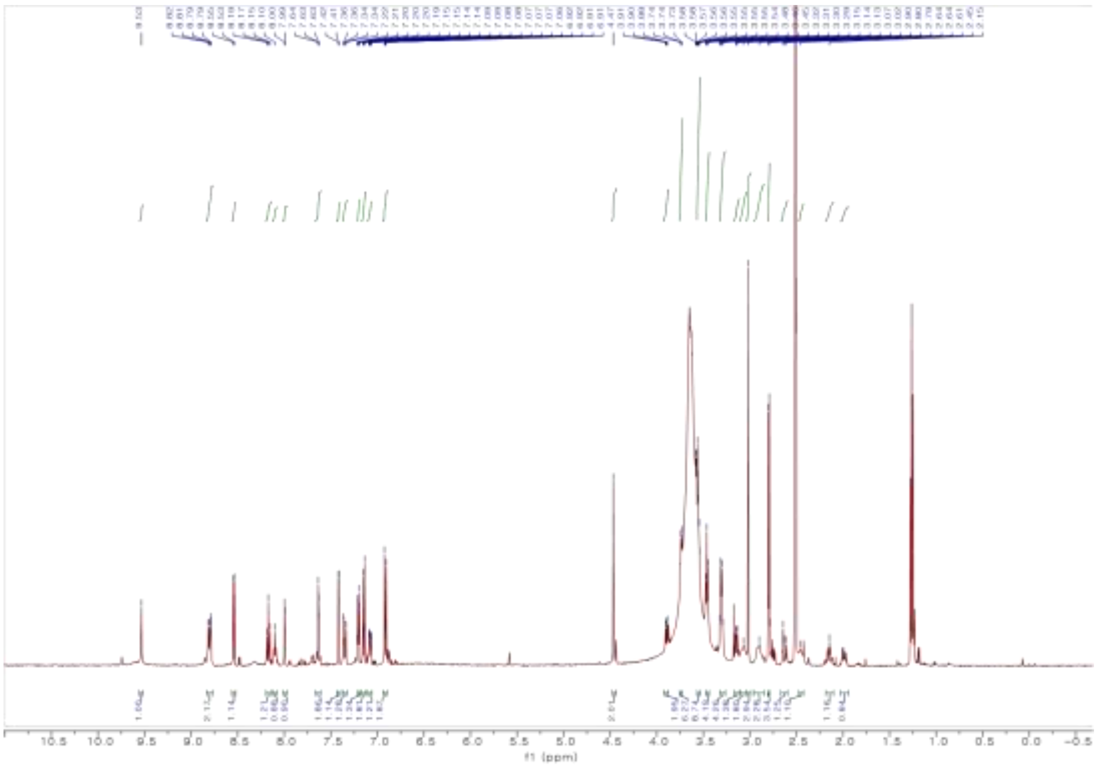

^{1}H NMR spectrum of **JHK-02-138 (JHK-02-102-1 neg)** in DMSO-d$_6$

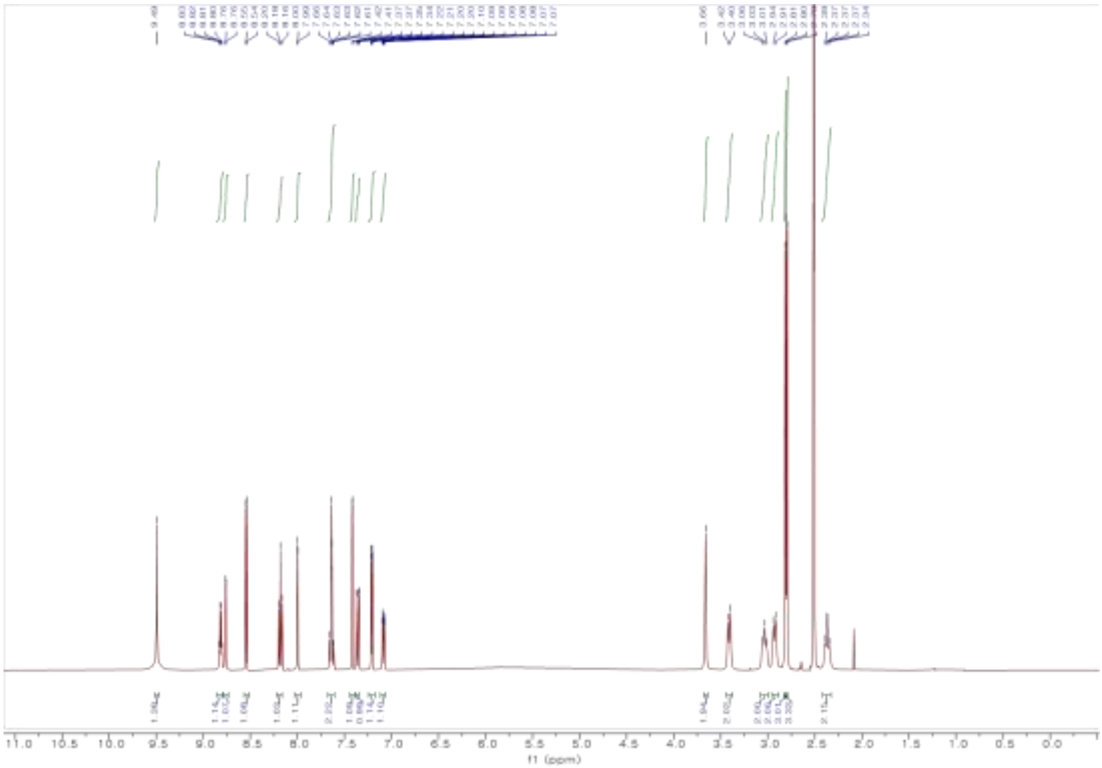

^{1}H NMR spectrum of **JHK-02-145** in DMSO-d$_6$

4. Western blot analysis from additional independent biological replicates

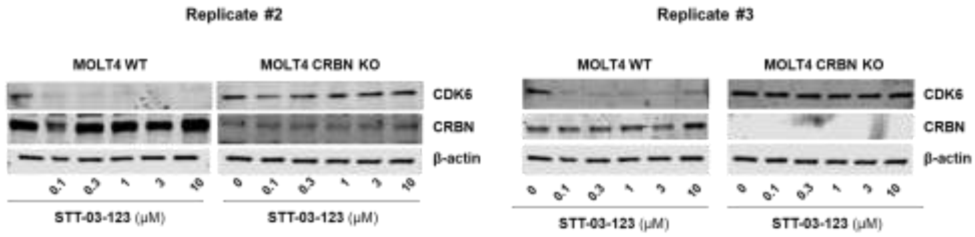

Western blot biological replicates for Figure 2C

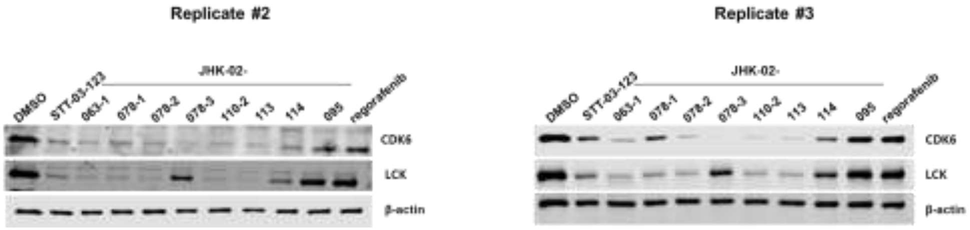

Western blot biological replicates for Figure 3B

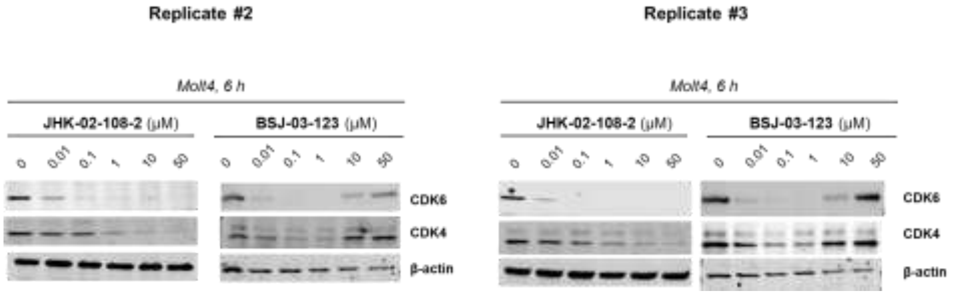

Western blot biological replicates for Figure 5B

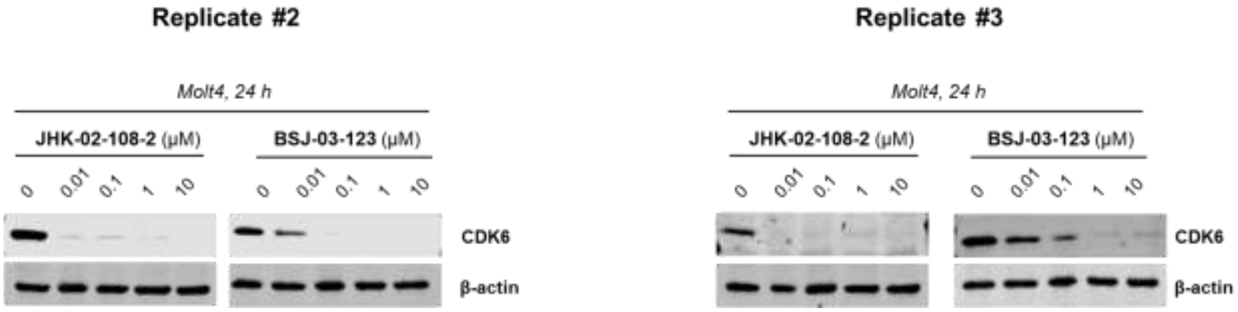

Western blot biological replicates for Figure 5C

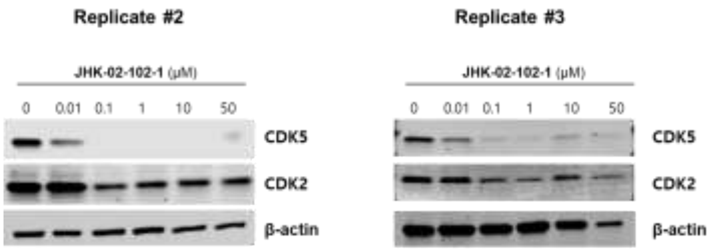

Western blot biological replicates for Figure 7B

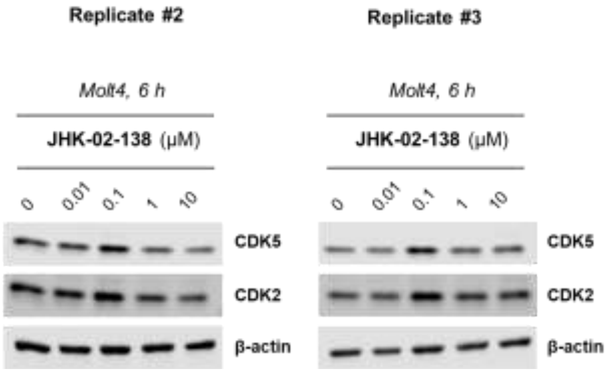

Western blot biological replicates for Figure 7C

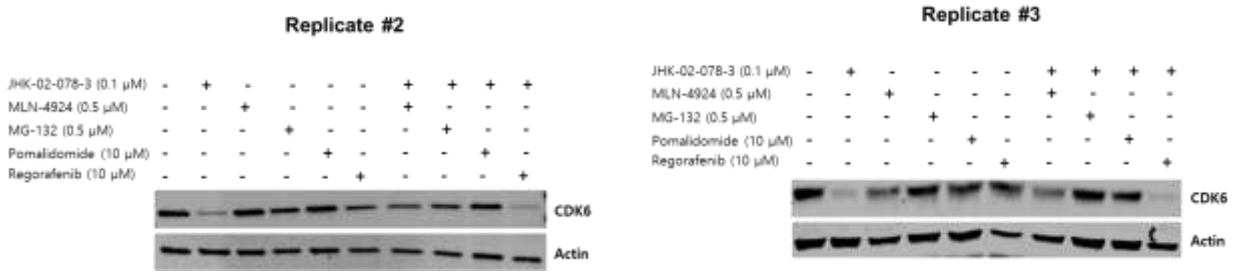

Western blot biological replicates for Figure S17

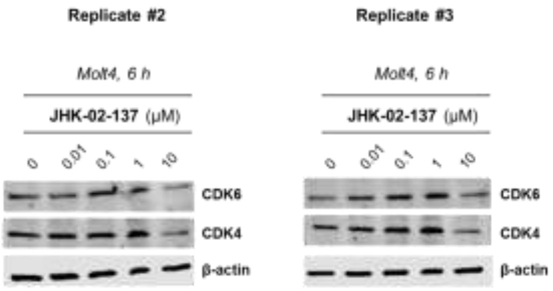

Western blot biological replicates for Figure S18

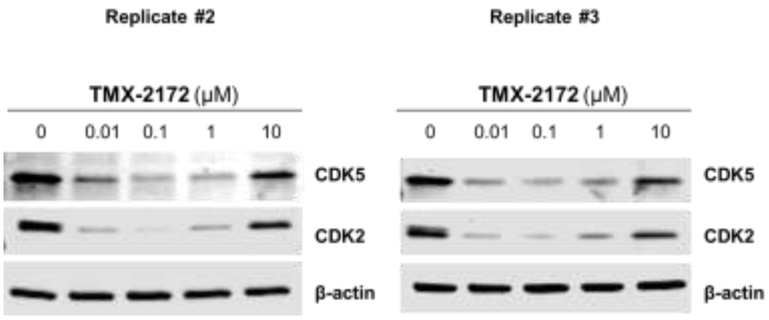

Western blot biological replicates for Figure S27

5. Uncropped blots

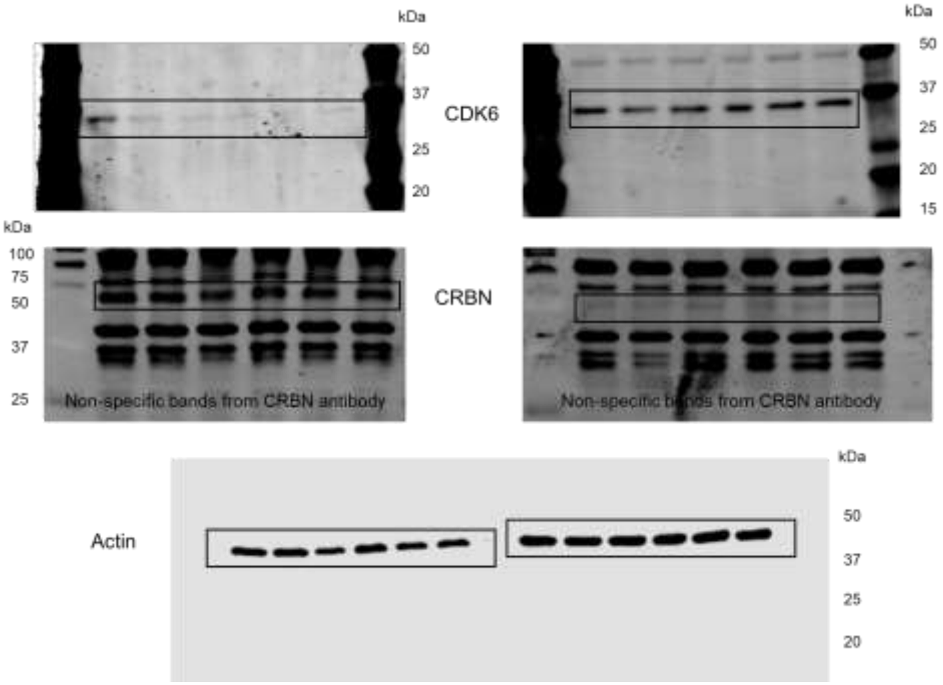

Uncropped blots for Figure 2C

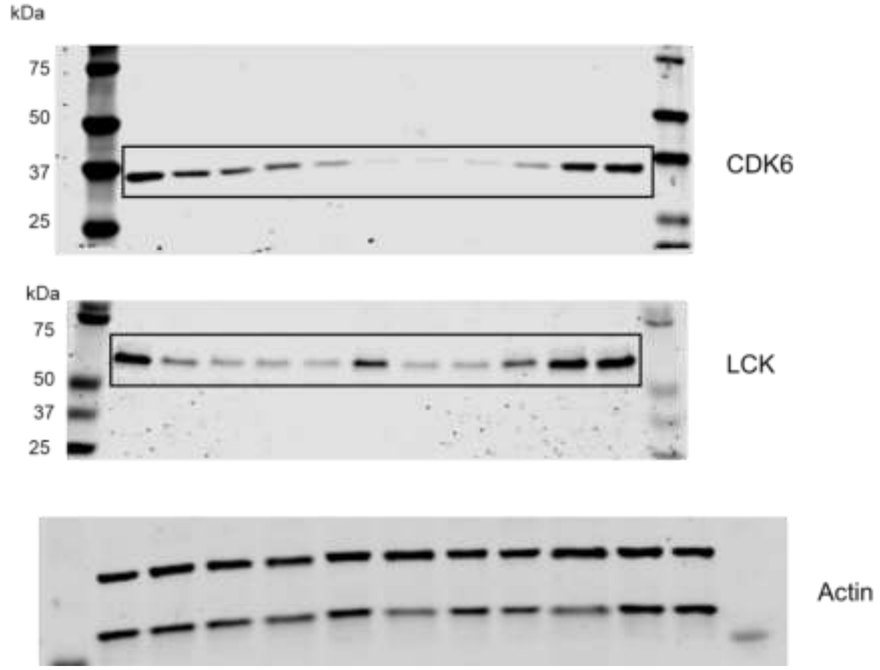

Uncropped blots for Figure 3A

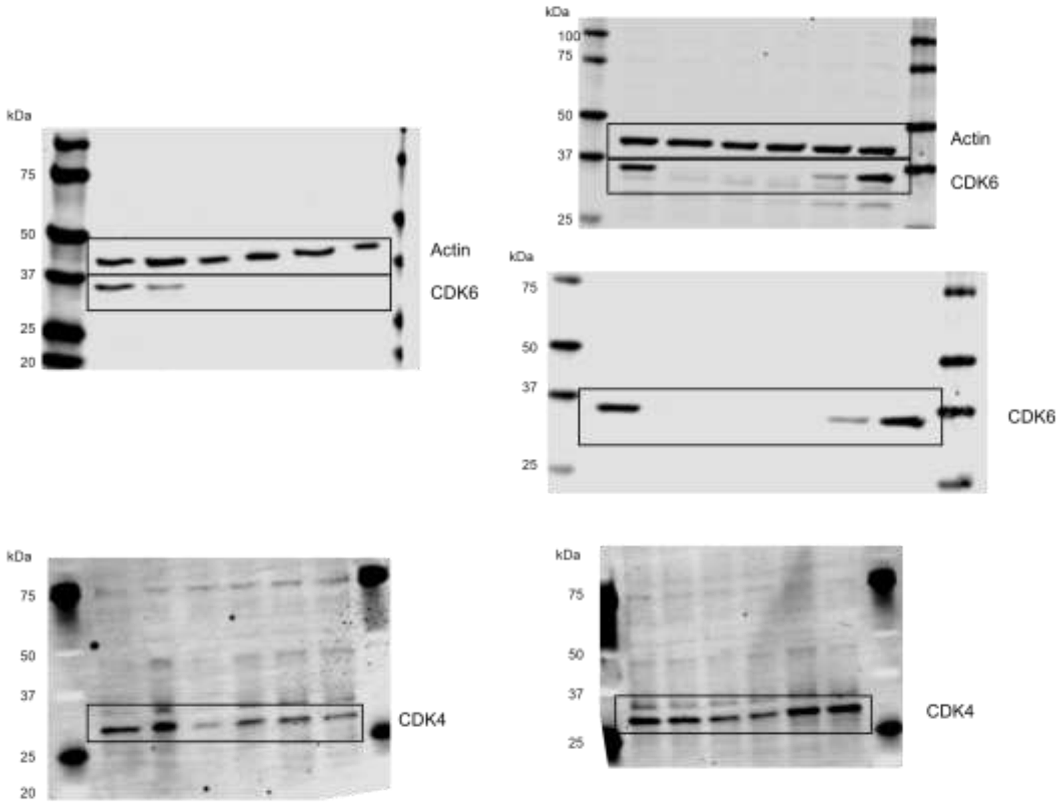

Uncropped blots for Figure 5B

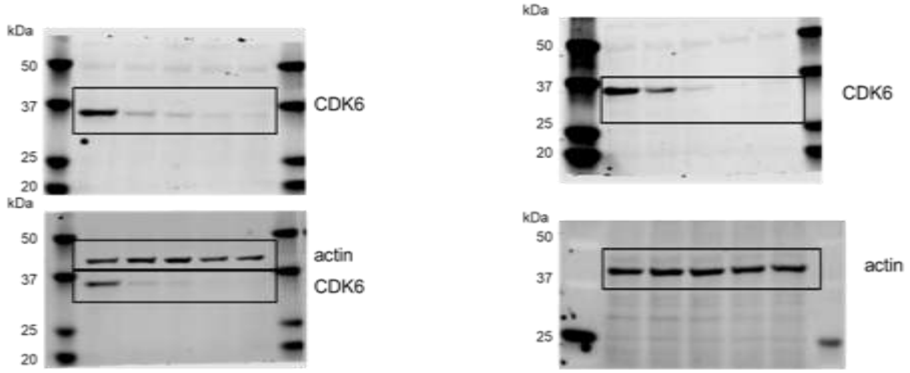

Uncropped blots for Figure 5C

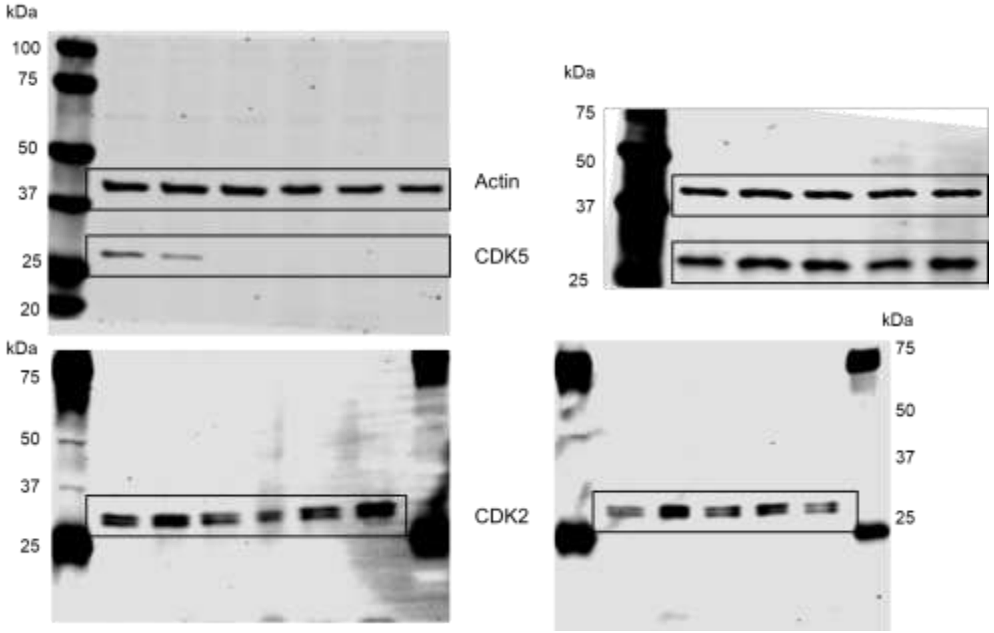

Uncropped blots for Figure 7C

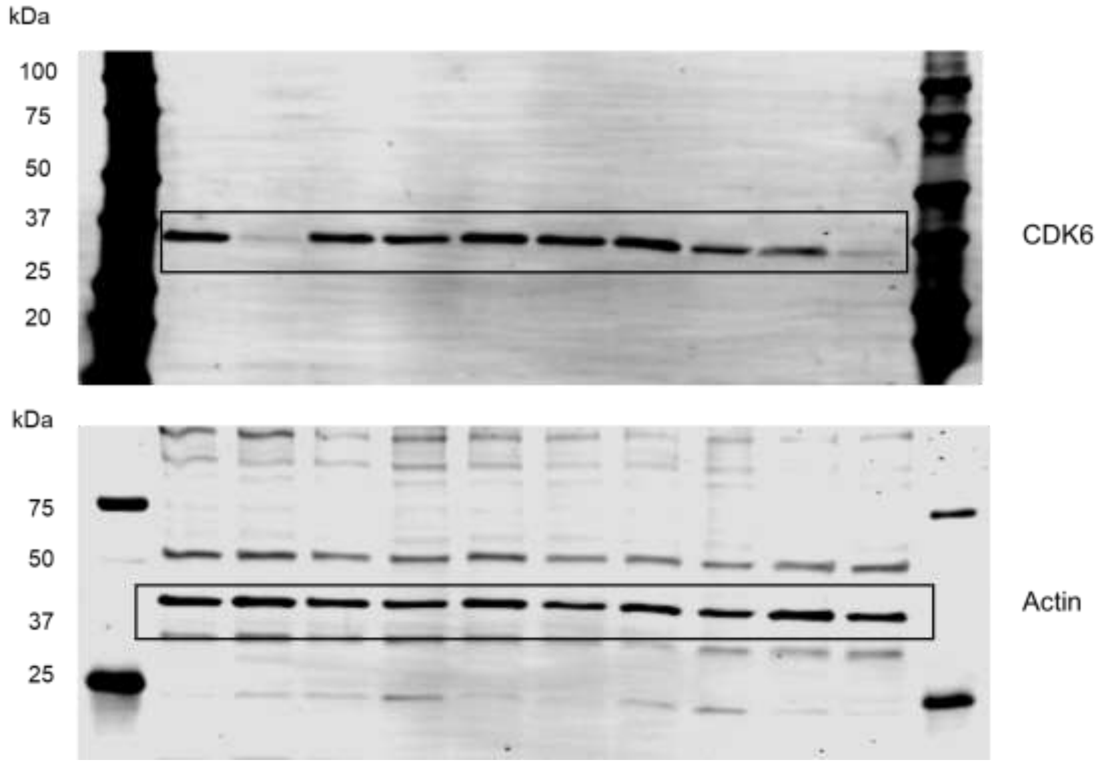

Uncropped blots for Figure S17

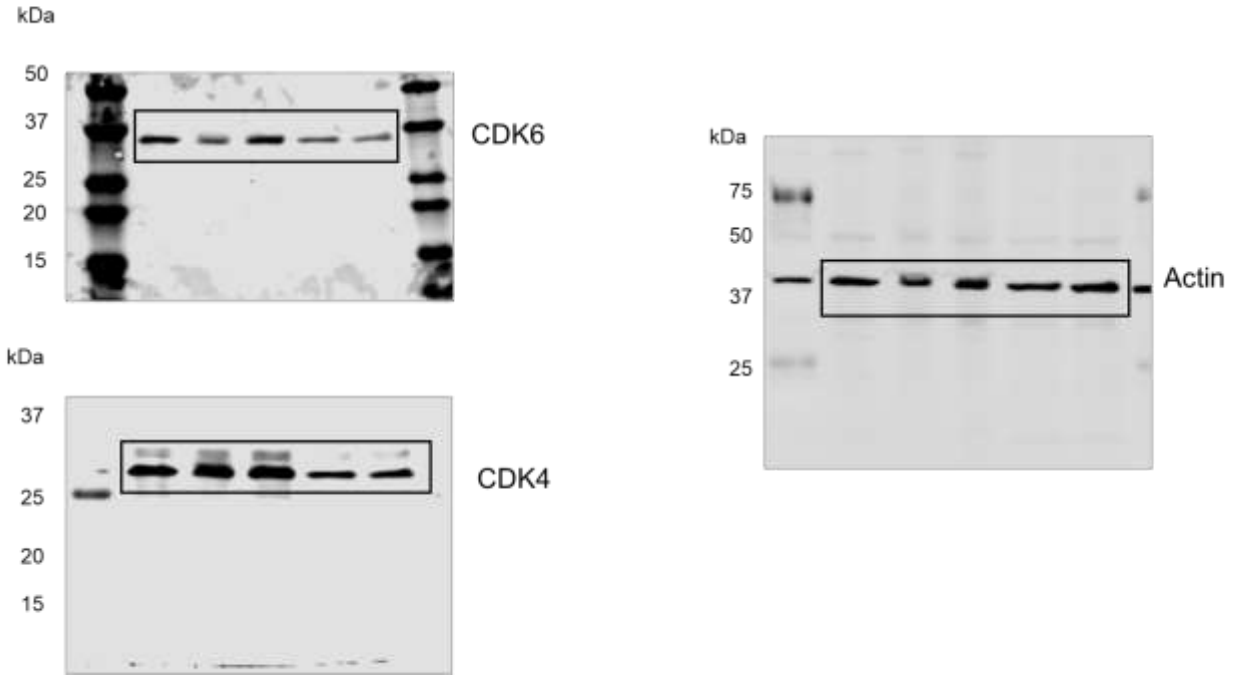

Uncropped blots for Figure S18

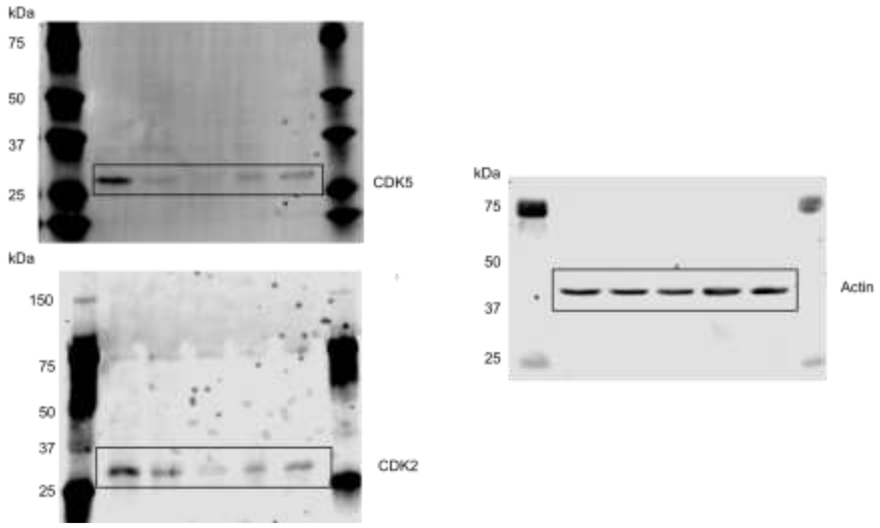

Uncropped blots for Figure S27

6. References

1. Touat, M.; Li, Y. Y.; Boynton, A. N.; Spurr, L. F.; Iorgulescu, J. B.; Bohrson, C. L.; Cortes-Ciriano, I.; Birzu, C.; Geduldig, J. E.; Pelton, K., Mechanisms and therapeutic implications of hypermutation in gliomas. *Nature* **2020,** *580* (7804), 517-523.
2. Mills, C. E.; Subramanian, K.; Hafner, M.; Niepel, M.; Gerosa, L.; Chung, M.; Victor, C.; Gaudio, B.; Yapp, C.; Nirmal, A. J., Multiplexed and reproducible high content screening of live and fixed cells using Dye Drop. *Nat. Commun.* **2022,** *13* (1), 6918.
3. Hafner, M.; Niepel, M.; Chung, M.; Sorger, P. K., Growth rate inhibition metrics correct for confounders in measuring sensitivity to cancer drugs. *Nat. Methods* **2016,** *13* (6), 521-527.
4. Scott, D. C.; Chittori, S.; Purser, N.; King, M. T.; Maiwald, S. A.; Churion, K.; Nourse, A.; Lee, C.; Paulo, J. A.; Miller, D. J., Structural basis for C-degron selectivity across KLHDCX family E3 ubiquitin ligases. *Nat. Commun.* **2024,** *15* (1), 9899.
5. Weissmann, F.; Petzold, G.; VanderLinden, R.; Huis In't Veld, P. J.; Brown, N. G.; Lampert, F.; Westermann, S.; Stark, H.; Schulman, B. A.; Peters, J.-M., biGBac enables rapid gene assembly for the expression of large multisubunit protein complexes. *Proc. Natl. Acad. Sci. U. S. A.* **2016,** *113* (19), E2564-E2569.
6. Scott, D. C.; Sviderskiy, V. O.; Monda, J. K.; Lydeard, J. R.; Cho, S. E.; Harper, J. W.; Schulman, B. A., Structure of a RING E3 trapped in action reveals ligation mechanism for the ubiquitin-like protein NEDD8. *Cell* **2014,** *157* (7), 1671-1684.
7. Donovan, K.A., An, J., Nowak, R.P., Yuan, J.C., Fink, E.C., Berry, B.C., Ebert, B.L., and Fischer, E.S. Thalidomide promotes degradation of SALL4, a transcription factor implicated in Duane Radial Ray syndrome. *Elife* **2018**. *7*. 10.7554/eLife.38430.
8. Skowronek, P.; Thielert, M.; Voytik, E.; Tanzer, M. C.; Hansen, F. M.; Willems, S.; Karayel, O.; Brunner, A.-D.; Meier, F.; Mann, M., Rapid and in-depth coverage of the (phospho-) proteome with deep libraries and optimal window design for dia-PASEF. *Mol. Cell. Proteomics* **2022,** *21* (9).

9. Reichermeier, K.M., Straube, R., Reitsma, J.M., Sweredoski, M.J., Rose, C.M., Moradian, A., den Besten, W., Hinkle, T., Verschueren, E., and Petzold, G. PIKES analysis reveals response to degraders and key regulatory mechanisms of the CRL4 network. *Mol. Cell.* **2020,** *77*, 1092-1106. e1099.
10. Demichev, V., Messner, C.B., Vernardis, S.I., Lilley, K.S., and Ralser, M. DIA-NN: neural networks and interference correction enable deep proteome coverage in high throughput. *Nat. Methods* **2020,** *17*, 41-44.
11. Baek, K., Metivier, R.J., Roy Burman, S.S., Bushman, J.W., Yoon, H., Lumpkin, R.J., Ryan, J.K., Abeja, D.M., Lakshminarayan, M., and Yue, H. Unveiling the hidden interactome of CRBN molecular glues. *Nat. Commun.* **2025,** *16*, 6831.
12. Ritchie, M.E., Phipson, B., Wu, D., Hu, Y., Law, C.W., Shi, W., and Smyth, G.K. limma powers differential expression analyses for RNA-sequencing and microarray studies. *Nucleic Acids* **2015,** Res *43*, e47. 10.1093/nar/gkv007.
